# Global, regional, and national burdens of late-onset epilepsy in adults aged 65 years and older from 1990 to 2021: A population-based study

**DOI:** 10.1371/journal.pone.0336588

**Published:** 2025-11-19

**Authors:** Zhijun Wang, Minheng Zhang, Hongwei Liu, Haixia Fan

**Affiliations:** 1 Department of Neurology, Shanxi Bethune Hospital, Shanxi Academy of Medical Sciences, Third Hospital of Shanxi Medical University, Tongji Shanxi Hospital, Taiyuan, China; 2 Department of Gerontology, The First People’s Hospital of Jinzhong, Yuci, Shanxi, China; 3 Department of Neurology, Taiyuan Central City Hospital, Taiyuan, Shanxi Province, China; 4 Department of Sleep Center, First Hospital of Shanxi Medical University, Taiyuan, Shanxi Province, China; Foundation IRCCS Carlo Besta Neurological Institute: Fondazione IRCCS Istituto Neurologico Carlo Besta, ITALY

## Abstract

**Objective:**

Epilepsy remains one of the most widespread and severe neurological disorders worldwide. This study aims to evaluate the burden of late-onset epilepsy (LOE) and investigate its temporal trends and inequalities among older adults at global, regional, and national scales between 1990 and 2021.

**Methods:**

This analysis utilizes data from the 2021 Global Burden of Diseases, Injuries, and Risk Factors Study (GBD). Temporal trends in the age-standardized prevalence rate (ASPR), incidence rate (ASIR), mortality rate (ASMR), and disability-adjusted life years (DALYs) rate for LOE were quantified through the calculation of average annual percentage change over the study period.

**Results:**

In 2021, the global ASPR and ASIR of LOE in adults aged 65 and older were estimated at 472.74 (95% UI: 332.21 to 654.96) and 33.12 (95% UI: 18.68 to 50.29) per 100,000 population, respectively. The global ASMR was 4.76 per 100,000 population (95% UI: 3.80 to 5.26), while the age-standardized DALYs rate reached 189.08 per 100,000 population (95% UI: 137.47 to 259.90). Among the five sociodemographic index (SDI) regions, high-SDI areas exhibited the greatest ASPR, ASIR, ASMR, and age-standardized DALY rate, whereas high-middle SDI regions reported the lowest. Geospatially, Andean Latin America recorded the highest ASPR, while Western Europe reported the highest ASIR. The highest ASMR and age-standardized DALY rate were observed in Central and Eastern Sub-Saharan Africa, respectively. Among 204 countries, Equatorial Guinea displayed the highest ASPR, while Germany had the highest ASIR. Notably, Zambia exhibited both the highest ASMR and age-standardized DALY rate for LOE. However, the inequalities associated with the SDI across countries gradually diminished over time.

**Conclusions:**

The study suggest that regions with high SDI continued to experience elevated ASPR, ASIR, ASMR, and age-standardized DALY rates. These findings highlight the importance of integrating LOE care into health systems, particularly for adults aged 65 years and older.

## Introduction

Epilepsy is a long-standing neurological disorder, identified by the brain’s persistent tendency to generate repeated seizures, impacting individuals across various geographies, ages, and ethnicities [1,2]. Although there have been therapeutic advancements, the majority of people with active epilepsy, over 75%, remain untreated, with the treatment gap being especially wide in low- and middle-income countries [3]. In the context of global population ageing, late-onset epilepsy (LOE), defined as seizure onset at ≥65 years [4], is gaining prominence as a clinical and public-health priority due to its links with vascular and neurodegenerative comorbidity. Although its pathogenesis remains incompletely resolved, converging evidence implicates cerebrovascular injury, neurodegenerative processes, multimorbidity, and frailty as key contributors to susceptibility and poorer outcomes [3,5]. Current management in older adults remains largely symptomatic, targeting seizure suppression rather than underlying pathophysiology [6,7]. Importantly, LOE is associated with substantially higher mortality than in age-matched populations without epilepsy, with excess deaths frequently linked to comorbid conditions such as stroke and dementia [8]. Thus, premature mortality is a critical issue for LOE patients worldwide, placing a heavy load on healthcare systems and impacting the patients and their families. It is vital for healthcare experts in every discipline to have a thorough understanding of this condition [9–12].

The burden of LOE is largely affected by social determinants of health, aside from clinical mechanisms. Older adults with epilepsy are disproportionately affected by poverty, limited educational attainment, and social isolation, which restrict access to timely diagnosis, sustained treatment, and newer antiseizure medications (ASMs) [3,10,13]. Stigma and discrimination act as barriers to seeking help and maintaining adherence, thereby reducing quality of life and well-being. The economic consequences surpass direct medical expenses, involving caregiver load, productivity losses, and long-term care costs, especially in places with insufficient social protection. The ongoing treatment gap in elderly populations in many low- and middle-income countries is due to a deficit of neurologists, limited diagnostic capabilities, and the unaffordability of vital ASMs, which intensifies cross-country health outcome disparities [14,15]. Considering LOE in the broader societal and public health context reveals the need for holistic strategies that incorporate social, economic, and health system interventions, in addition to seizure control.

Even though it is becoming more common and impactful, LOE is still relatively underexplored in the field of geriatric neurology. Prior investigations are usually limited to specific regions, apply different age criteria, or are missing long-term data, which results in poorly defined global trends.To address these gaps, we leveraged data from the Global Burden of Disease (GBD) 2021 study to quantify the prevalence, incidence, mortality, and disability-adjusted life years (DALYs) attributable to LOE among adults aged 65 years and older, disaggregated by age, sex, and Sociodemographic Index (SDI), across 204 countries and territories from 1990 to 2021. This study, by combining temporal trends with sociodemographic context, offers the first systematic, thirty-year assessment of the global LOE burden, providing insights to inform prevention strategies, optimize clinical management,and guide resource allocation in ageing societies.

## Methods

### Study design and data sources

This analysis, which is population-based and cross-sectional, utilized data from the GBD 2021 study to estimate the LOE burden among those aged 65 and older in 204 countries and territories from 1990 to 2021. The ethical board of Taiyuan Central Hospital granted a waiver of informed consent as the study only involved data analysis and no identifiable personal information.

The Global Health Data Exchange (GHDx) query tool, an online platform created by the GBD Collaborators, was utilized to extract data, offering standardized and comparable estimates for 369 diseases, injuries, and impairments, and 88 risk factors on a global scale. The dataset offers age-, sex-, and location-specific statistics on prevalence, incidence, mortality, and DALYs, with corresponding 95% uncertainty intervals (UIs). This analysis involved extracting data for seven older age ranges: 65–69, 70–74, 75–79, 80–84, 85–89, 90–94, and 95 years and up.

### Case definition and modeling framework

In the context of the GBD 2021 framework, epilepsy was identified through physician-diagnosed cases, which were confirmed using hospital discharge records, clinical registries, or national surveillance databases. According to Josephson and his colleagues, LOE is defined as epilepsy that begins at the age of 65 or later [16,17]. Using DisMod-MR 2.1, a Bayesian meta-regression model, non-fatal outcomes were estimated by synthesizing epidemiological data on incidence, prevalence, DALYs, and mortality to maintain internal consistency. By using spatiotemporal Gaussian process regression, missing or incomplete data were tackled, incorporating information from multiple age groups, locations, and time periods to boost precision and decrease bias. Each estimate signifies the mean of 1,000 posterior draws, with the 95% uncertainty intervals marking the 2.5th and 97.5th percentiles of these draws.

### Sociodemographic index

To evaluate the developmental status of countries or regions, the SDI uses a combination of indicators such as fertility rates, educational attainment, and income per capita [18]. The SDI measures socioeconomic development on a scale from 0 to 1, with higher values denoting more advanced development. Differences in SDI are linked to variations in disease prevalence and mortality rates. For this analysis, countries and regions were organized into five SDI categories (low, low-medium, medium, medium-high, and high) to study the association between socioeconomic development and the burden of LOE in adults.

### Measures of health inequality

Evaluating health inequalities is necessary for evidence-based health planning, facilitating the crafting of policies, programs, and interventions to diminish health disparities [19,20]. The study applied two well-known metrics of absolute and relative gradient inequality, namely the slope index of inequality and the concentration index, to analyze the distributional inequality of LOE burden across different countries. National DALYs rates across all age groups were regressed against a relative position scale linked to sociodemographic development to derive the slope index of inequality. Using the midpoint of the cumulative population range ranked by the SDI, we determined this relative position. To manage heteroskedasticity, we applied a weighted regression model. To calculate the concentration index, the area under the Lorenz concentration curve was integrated, showing the cumulative proportion of DALYs against the cumulative share of the population ranked by SDI [21].

### Trend analysis

Using Joinpoint regression, temporal trends in the LOE burden were evaluated (Joinpoint Regression Program, version 5.0.2; National Cancer Institute, USA).By employing permutation tests with 4,499 resamples (p < 0.05), this method identifies statistically significant changes in linear trends over time, known as ‘joinpoints’. For each indicator, including the age-standardized prevalence rate (ASPR), incidence rate (ASIR), mortality rate (ASMR), and disability-adjusted life years (DALYs) rate, we calculated the average annual percentage change (AAPC) with its 95% confidence interval to summarize long-term temporal trends. Throughout the study period, a positive AAPC denotes an increasing trend, while a negative AAPC denotes a decreasing trend.

### Statistical analysis

The present study aimed to characterize the global, regional, and national burden of LOE in adults aged 65 years and older. This analysis examined variations in age-standardized prevalence, incidence, mortality, and DALY rates per 100,000 population across different demographic and geographic strata. Utilizing data from the GBD Study, we computed age-standardized rates and their 95% confidence intervals (CIs) based on the world standard population outlined in the 2021 GBD Study. This approach facilitated regional comparisons, and joinpoint regression was employed to estimate average annual percentage change (AAPC) and to analyze temporal trends [17,22]. Our estimates were displayed per 100,000 population using the top equation displayed in S1 Fig. AAPC measure the average annual percentage change in a variable over a specified time period. In this study, AAPC represent the annual percentage change derived from the weighted average of slope coefficients in the joinpoint regression model, spanning from 1990 to 2021 [23]. The AAPC value reflects the percentage change per year, indicating whether there is an increase, decrease, or stability in the trend. When both the annual percentage change estimates and their 95% CIs are consistently positive or negative, the trend is interpreted as either rising or declining, respectively. The calculation of AAPC is based on the formula provided in S1 Fig. Our analysis included epidemiologically related measures, such as prevalence, incidence, DALYs (where one DALY denotes the loss of one year of health due to death or disability), and mortality. For example, a reduction in mortality from a chronic condition might result in an increase in both prevalence and DALYs, as more individuals live longer with the condition. Conversely, an increase in new cases or extended duration of illness contributes to a higher prevalence. The observed rise in LOE prevalence among individuals aged 65 years and older may be attributed to advancements in medical care, which extend the duration of life with the disease. The analyses and visualizations were carried out using the Health Equity Assessment Toolkit by the World Health Organization and R software (Version 4.3.3).

## Results

### Global trends

Between 1990 and 2021, the global prevalence of LOE among individuals aged 65 years and older increased significantly by 193.62%, rising from 1.22 million to 3.57 million cases. The ASPR of LOE grew by 21.82%, from 388.06 per 100,000 population in 1990 to 472.74 per 100,000 population in 2021 (Table 1, S2 Fig), with an AAPC of 0.65% (Table 1, S3 Fig). The global incidence of LOE in this age group reached 0.25 million in 2021, reflecting a 212.84% increase since 1990. The ASIR of LOE experienced a modest rise of 30.71%, climbing from 25.04 per 100,000 population in 1990 to 33.12 per 100,000 population in 2021(Table 1, S2 Fig), with an AAPC of 0.84% (Table 1, S3 Fig). Over the past three decades, the GBD, as measured by DALYs attributable to LOE, increased by 142.37%, rising from 0.59 million to 1.43 million. The age-standardized DALY rate for LOE grew by 2.8%, from 183.90 per 100,000 population in 1990 to 189.08 per 100,000 in 2021 (Table 2, S2 Fig), reflecting an AAPC of 0.10% (Table 2, S3 Fig). In 2021, LOE-related mortality was estimated at 35,181.86 deaths, with the ASMR rising by 7.94%, from 4.41 per 100,000 population in 1990 to 4.76 per 100,000 in 2021 (Table 2, S2 Fig), representing an AAPC of of 0.29% (Table 2, S3 Fig). Compared to the trends in ASPR and ASIR, the increases in the age-standardized DALY rate and ASMR for LOE among individuals aged 65 and older were less pronounced over the same period.

**Table 1:** ASPR and ASIR of LOE in individuals aged ≥65 years and Their AAPC from 1990 to 2021 at Global and SDI levels.

	No of people with LOE in 1990	Age-standardised rate in 1990 (per 100 000)	No of people with LOE in 2021	Age-standardised rate in 2021 (per 100 000)	AAPC (95% CI)
	**Prevalence (95% UI)**				
Global	1220770.72 (879585.81 to 1659437.84)	388.06 (279.09 to 527.51)	3573526.69 (2512685.56 to 4957991.65)	472.74 (332.21 to 654.96)	0.65 (0.62 to 0.69)
Sex-female	655769.02 (469468.69 to 890435.93)	360.91 (258.01 to 490.37)	1865550.14 (1299811.43 to 2605140.07)	443.83 (309.4 to 619.4)	0.69 (0.65 to 0.73)
Sex-male	565001.71 (407752.35 to 769115.89)	429.26 (308.85 to 583.31)	1707976.55 (1204126.87 to 2348302.01)	510.79 (359.52 to 700.48)	0.57 (0.52 to 0.62)
High SDI	447779.57 (297928.75 to 623275.05)	433.26 (288.03 to 603.49)	1225612.14 (782980.64 to 1717149.76)	583.71 (373.32 to 819.58)	0.97 (0.89 to 1.04)
High-middle SDI	270066.90 (189815.29 to 373230.03)	332.95 (233.16 to 460.15)	703981.94 (471626.50 to 999966.57)	388.73 (260.11 to 551.58)	0.53 (0.48 to 0.58)
Middle SDI	269615.94 (181255.21 to 376152.61)	370.36 (248.17 to 517.14)	995575.04 (688727.15 to 1400911.00)	449.57 (310.46 to 631.41)	0.64 (0.55 to 0.73)
Low-middle SDI	164888.31 (99383.54 to 245573.11)	390.53 (235.67 to 582.41)	487286.42 (337109.34 to 677049.58)	444.94 (307.77 to 618.33)	0.40 (0.33 to 0.48)
Low SDI	66834.69 (36173.40 to 104248.62)	438.44 (236.82 to 686.58)	157667.94 (102735.66 to 227572.73)	455.18 (295.83 to 655.21)	0.11 (0.07 to 0.15)
	**Incidence (95% UI)**				
Global	81542.47 (46608.09 to 123973.40)	25.04 (14.31 to 38.07)	255096.67 (143886.99 to 387346.56)	33.12 (18.68 to 50.29)	0.84 (0.80 to 0.87)
Sex-female	42235.74 (23781.37 to 64755.13)	23.66 (13.23 to 36.72)	131735.70 (72760.58 to 203281.50)	31.32 (17.31 to 48.28)	0.93 (0.86 to 1.00)
Sex-male	39306.73 (22676.12 to 59220.68)	30.36 (17.38 to 46.66)	123360.97 (71500.10 to 183971.87)	37.14 (21.38 to 55.94)	0.67 (0.62 to 0.72)
High SDI	30975.93 (17514.35 to 48147.36)	30.19 (16.97 to 47.24)	91307.94 (49769.44 to 140225.24)	43.19 (23.71 to 65.84)	1.16 (1.02 to 1.30)
High-middle SDI	15733.10 (8587.95 to 24582.31)	19.68 (10.65 to 31.24)	46328.69 (24473.68 to 72948.75)	25.69 (13.52 to 40.70)	0.91 (0.80 to 1.03)
Middle SDI	17883.68 (9699.75 to 28114.51)	24.95 (13.40 to 40.12)	68216.00 (37385.11 to 106289.86)	31.02 (16.92 to 48.96)	0.76 (0.68 to 0.83)
Low-middle SDI	11967.05 (5977.80 to 20021.93)	28.91 (14.46 to 49.22)	36927.62 (20659.49 to 57876.12)	34.31 (19.14 to 54.50)	0.54 (0.48 to 0.61)
Low SDI	4902.90 (2307.67 to 8568.04)	34.06 (16.19 to 60.81)	12124.90 (6813.28 to 19109.89)	37.25 (20.83 to 59.71)	0.28 (0.26 to 0.31)

**Abbreviations:** ASIR, age-standardized incidence rate; ASPR, age-standardized prevalence rate; AAPC, average annual percent change; CI, confidence interval; P, P value for the significant test of AAPC; LOE, late-onset epilepsy; SDI, sociodemographic index. Numbers in parentheses are 95% uncertainty intervals (Cases and age standardized rate) and 95% confidence interval (AAPC).

**Table 2:** ASMR and age-standardized DALYs rate and AAPC of LOE in individuals aged ≥65 years from 1990 to 2019 at Global and SDI levels.

	No of people with LOE in 1990	Age standardised rate in 1990 (per 100 000)	No of people with LOE in 2021	Age standardised rate in 2021 (per 100 000)	AAPC (95% CI)	P value
	**Mortality (95% UI)**					
Global	13498.39 (11026.29 to 15185.58)	4.41 (3.60 to 4.97)	35181.86 (28042.27 to 38795.29)	4.76 (3.80 to 5.26)	0.29 (0.05 to 0.53)	0.020
Sex-female	5737.73 (4055.77 to 6819.83)	3.24 (2.29 to 3.85)	17248.14 (12112.49 to 19731.64)	4.10 (2.88 to 4.69)	0.82 (0.63 to 1.00)	<0.001
Sex-male	7760.66 (6294.39 to 9277.02)	6.16 (5.01 to 7.36)	17933.72 (14423.7 to 20242.18)	5.61 (4.53 to 6.33)	−0.27 (−0.57 to 0.04)	0.083
High SDI	2395.67 (2203.40 to 2520.43)	2.34 (2.15 to 2.47)	9032.11 (7587.27 to 9991.43)	4.07 (3.45 to 4.48)	1.79 (1.54 to 2.04)	<0.001
High-middle SDI	1606.87 (1437.57 to 1855.35)	2.11 (1.88 to 2.45)	4879.82 (4039.92 to 5404.70)	2.82 (2.33 to 3.12)	0.97 (0.70 to 1.24)	<0.001
Middle SDI	2471.84 (2088.19 to 2948.95)	3.69 (3.09 to 4.39)	6164.23 (5047.94 to 7043.36)	2.94 (2.40 to 3.37)	−0.70 (−0.98 to −0.43)	<0.001
Low-middle SDI	3680.10 (2455.00 to 4501.49)	9.22 (6.15 to 11.36)	9029.33 (6148.30 to 10550.91)	8.74 (5.92 to 10.28)	−0.12 (−0.53 to 0.30)	0.579
Low SDI	3331.21 (2571.39 to 4107.20)	23.06 (17.57 to 28.66)	6044.00 (4650.04 to 7169.16)	18.52 (14.04 to 22.08)	−0.67 (−0.81 to −0.53)	<0.001
	**DALYs (95% UI)**					
Global	590352.84 (437192.49 to 780160.45)	183.90 (135.85 to 243.50)	1437351.57 (1046166.25 to 1974917.62)	189.08 (137.47 to 259.90)	0.10 (0.02 to 0.17)	0.012
Sex-female	284797.86 (202499.33 to 384496.32)	155.15 (110.20 to 209.70)	716462.47 (513374.75 to 990696.14)	170.33 (122.01 to 235.51)	0.32 (0.26 to 0.37)	<0.001
Sex-male	305554.98 (233437.58 to 398070.37)	224.89 (171.27 to 293.17)	720889.10 (525604.84 to 987758.9)	212.54 (154.87 to 291.36)	−0.16 (−0.26 to −0.05)	0.003
High SDI	141892.41 (93703.43 to 215520.15)	137.00 (90.41 to 208.14)	374868.13 (243920.71 to 576838.66)	178.61 (115.97 to 275.36)	0.86 (0.72 to 0.99)	<0.001
High-middle SDI	102642.42 (71140.79 to 145487.24)	125.56 (86.94 to 177.79)	233671.79 (157111.44 to 346595.26)	129.29 (87.05 to 191.28)	0.12 (−0.08 to 0.32)	0.232
Middle SDI	132909.93 (93984.94 to 182302.28)	179.20 (126.49 to 245.6)	364191.82 (250929.48 to 521252.12)	163.03 (112.25 to 233.32)	−0.28 (−0.42 to −0.15)	<0.001
Low-middle SDI	125344.35 (89509.61 to 168150.59)	289.18 (205.74 to 388.96)	302376.6 (225188.09 to 390061.07)	272.25 (202.27 to 351.47)	−0.18 (−0.36 to 0.00)	0.054
Low SDI	86902.64 (66571.17 to 110658.7)	546.69 (415.24 to 699.06)	160901.96 (126220.06 to 199181.43)	450.85 (352.09 to 559.45)	−0.61 (−0.7 to −0.53)	<0.001

**Abbreviations:** ASMR, age-standardized mortality rate; AAPC, average annual percent change; DALYs, disability-adjusted life years; CI, confidence interval; P, P value for the significant test of AAPC; LOE, late-onset epilepsy; SDI, sociodemographic index. Numbers in parentheses are 95% uncertainty intervals (Cases and age standardized rate) and 95% confidence interval (AAPC).

### Global trends by sex

Between 1990 and 2021, the global ASPR and ASIR of LOE among individuals aged 65 years and older exhibited a sustained upward trajectory in both sexes. Specifically, the ASPR in men increased from 429.26 to 510.79 per 100,000 population, while in women it rose from 360.91 to 443.83 per 100,000 population (Table 1). This upward shift was more pronounced in women, with an AAPC of 0.69%, compared to 0.57% in men (Table 1). A similar trend was observed in the ASIR, which climbed from 30.36 to 37.14 per 100,000 population in men and from 23.66 to 31.32 per 100,000 population in women (Table 1). Once again, the increase was more substantial in women, as reflected by an AAPC of 0.93%, relative to 0.67% in men (Table 1).

By contrast, the ASMR for LOE displayed divergent trends between sexes. In men, the ASMR decreased from 4.10 to 3.24 per 100,000 population, corresponding to an AAPC of −0.27% between 1990 and 2021 (Table 2). In women, however, mortality rose from 5.61 to 6.16 per 100,000 population, with an AAPC of 0.82% over the same period. (Table 2). A comparable divergence was noted in the age-standardized DALY rate associated with LOE. Among men, the rate declined from 224.89 to 212.54 per 100,000 population, whereas in women it increased from 155.15 to 170.33 per 100,000 population, with AAPCs of −0.16% and 0.32%, respectively (Table 2). The observed sex disparity remained evident across varying levels of the SDI, with men consistently bearing a higher disease burden than women, especially in nations classified within the low SDI range (S4 and S5 Figs).

### Global trends by age subgroup

Globally, the ASPR of LOE demonstrated a consistent upward trajectory across all age groups from 1990 to 2021, with the most pronounced growth observed among women. The sharpest rise in ASPR occurred in individuals aged 90–94 years, with an AAPC of 0.90% (S1 Table, S6 Fig). Similarly, the global ASIR showed a marked increase over the same period, most notably among those aged 80–89 years, with an AAPC of 1.01% (S1 Table, S6 Fig).

By contrast, the ASMR of LOE declined between 1990 and 2021 in the 65–69 and 70–74 age groups, with AAPCs of −0.37% and −0.35%, respectively. However, the ASMR increased in individuals aged 75 and older, with the most pronounced rise in ASPR notably observed in the 90–94 age group, where the AAPC reached 1.58% (S1 Table, S6 Fig). A similar pattern was observed for age-standardized DALYs rate associated with LOE. Between 1990 and 2021, the age-standardized DALYs rate decreased in individuals aged 65–69 years (AAPC −0.18%) and 70–74 years (AAPC −0.12%). Conversely, the age-standardized DALYs rate increased among those aged 75 and older, with the sharpest rise in the age-standardized DALYs rate occurring in the 90–94 age group, where the AAPC reached 0.85% (S1 Table, S6 Fig).

### Global trends by sociodemographic index

Between 1990 and 2021, the ASPR and ASIR of LOE among individuals aged 65 years and older exhibited an upward trend across all sociodemographic index (SDI) subgroups. This increase was most pronounced in high-SDI countries, where the AAPC reached 1.16% for incidence and 0.97% for prevalence (Table 1). By 2021, the highest ASIR and ASPR were observed in high-SDI regions, with 43.19 per 100,000 population and 583.71 per 100,000 population, respectively (Table 1). In contrast, high-middle SDI countries recorded the lowest corresponding rates, at 25.69 per 100,000 population for ASIR and 388.73 per 100,000 population for ASPR. Notably, the ASPR and ASIR increases among the elderly population consistently exceeded those observed in the general population across all SDI groups (S7 and S8 Figs).

Over this period, the most substantial reduction in the ASMR of LOE occurred in middle-SDI countries, where the AAPC declined by −0.70%, a decline more than 5.83 times greater than the smallest reduction observed in low-middle SDI countries (AAPC −0.12%) (Table 2). In contrast, the largest rise in ASMR was observed in high-SDI countries, with an AAPC of 1.79%, a figure 1.85 times greater than the lowest increase recorded in high-middle SDI countries (AAPC 0.97%) (Table 2). Despite these trends, low-SDI countries exhibited the highest ASMR in 2021, at 18.52 per 100,000 population, a value 6.57 times higher than the lowest ASMR, which was observed in high-SDI countries (2.82 per 100,000 population) (S7 Fig, Table 2). In terms of DALYs, the age-standardized DALY rate for LOE among older adults decreased significantly across all SDI subgroups, except in high and high-middle SDI countries, where the AAPC increased by 0.86% and 0.12%, respectively (Table 2). In 2021, the age-standardized DALYs rate were highest in low-SDI countries (450.85 per 100,000 population) and lowest in high-middle SDI countries (129.29 per 100,000 population) (S7 Fig, Table 2). As the SDI increases, the decline in ASMR and age-standardized DALY rate attributable to LOE becomes increasingly pronounced (S9 Fig).

### Regional trends

Between 1990 and 2021, all regions, with the exception of Central Sub-Saharan Africa and Oceania, showed an increase in the ASPR of LOE among individuals aged 65 years and older. High-income North America exhibited the most rapid increase in ASPR, with an AAPC of 1.52% (S10 Fig). By 2021, the highest ASPR among individuals aged 65 and older were observed in Andean Latin America (778.66 per 100,000 population), Central Latin America (759.94 per 100,000 population), and Western Europe (685.50 per 100,000 population) (S2 Table). No significant differences were observed after gender stratification (S3 Table). Similarly, from 1990 to 2021, all regions, except Central Sub-Saharan Africa, experienced an increase in the ASIR of LOE among older adults. High-income North America again showed the most rapid rise in ASIR, with an AAPC of 1.61% (S10 Fig). In 2021, the highest ASIR among older adults were observed in Western Europe (55.42 per 100,000 population), Southern Sub-Saharan Africa (52.12 per 100,000 population), and Western Sub-Saharan Africa (50.50 per 100,000 population) (S2 Table). As with ASPR, no significant gender-based differences in ASIR were identified (S3 Table).

Between 1990 and 2021, 11 out of 21 regions experienced a reduction in mortality due to LOE among older adults, while 9 regions reported an increase, with varying rates of change. The most pronounced increase in the ASMR occurred in the High-income Asia Pacific region, with AAPC of 3.67% (S10 Fig). By 2021, the highest ASMR for LOE among older adults was observed in Central Sub-Saharan Africa, at 8.02 per 100,000 population (S4 Table). No significant differences were identified when stratified by gender (S3 Table). Over the same period, 12 out of 21 regions reported a reduction in DALYs attributed to LOE among older adults, while 9 regions experienced an increase. The most notable age-standardized DALYs rise was seen in High-income North America, with an AAPC of 1.28% in 2021 (S10 Fig). The highest age-standardized DALYs rise for LOE among older adults in 2021 was recorded in Eastern Sub-Saharan Africa, at 759.52 per 100,000 population (S4 Table). As with ASMR, no significant gender-based differences in age-standardized DALYs rise were identified (S4 Table and S11 Fig).

### National trends

**Fig 1:**
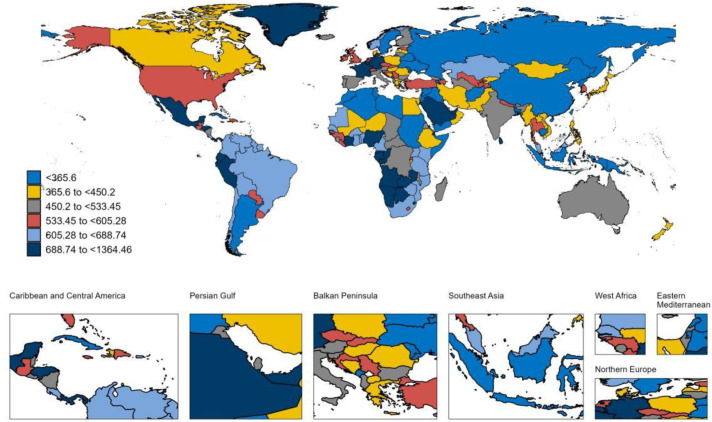
Map showing ASPR of LOE in individuals aged ≥65 years in 2021. **Abbreviations:** ASPR, age-standardized prevalence rate; LOE, late-onset epilepsy. **Note:** Base map from Natural Earth (public domain). Map created by the authors in R.

**Fig 2:**
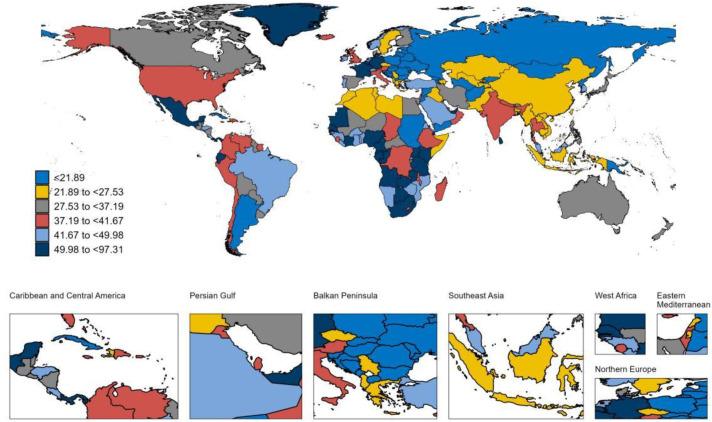
Map showing ASIR of LOE in individuals aged ≥65 years in 2021. **Abbreviations:** ASIR, age-standardized incidence rate; LOE, late-onset epilepsy. **Note:** Base map from Natural Earth (public domain). Map created by the authors in R.

**Fig 3:**
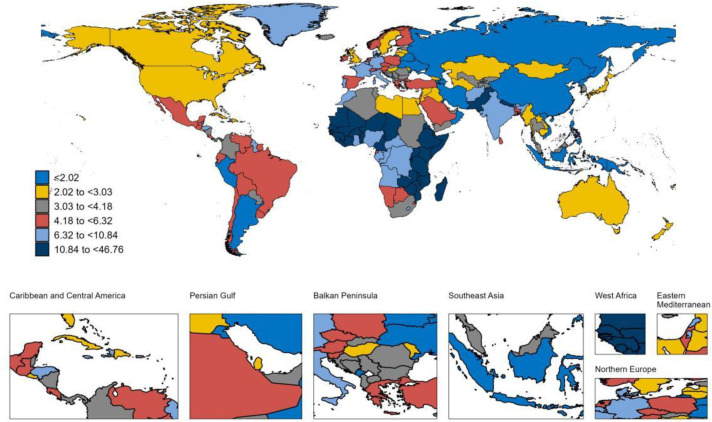
Map showing ASMR of LOE in individuals aged ≥65 years in 2021. **Abbreviations:** ASMR, age-standardized mortality rate; LOE, late-onset epilepsy. **Note:** Base map from Natural Earth (public domain). Map created by the authors in R.

**Fig 4:**
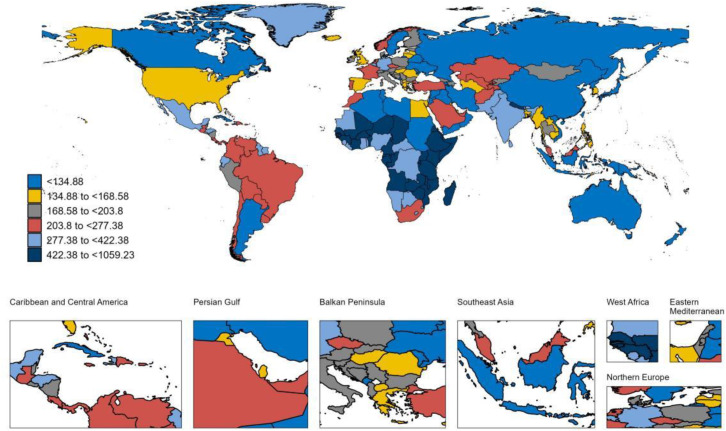
Map showing age-standardized DALYs rate of LOE in individuals aged ≥65 years in 2021. **Abbreviations:** DALYs, disability-adjusted life-years; LOE, late-onset epilepsy. **Note:** Base map from Natural Earth (public domain). Map created by the authors in R.

At the national level, between 1990 and 2021, Equatorial Guinea experienced the largest increase in the ASPR of LOE among individuals aged 65 years and older, with an AAPC (AAPC) of 2.62% (S5 Table) (Fig 1). This was followed by Germany (AAPC 2.06%) and the United States (AAPC 1.67%). Germany also reported the highest increase in the ASIR for LOE in this age group, with an AAPC of 2.68%, followed by Equatorial Guinea (AAPC 2.47%) and Oman (AAPC 2.07%) (S5 Table) (Fig 2). During this period, Japan exhibited the largest increase in the ASMR among older adults with LOE, at an AAPC of 5.34% (S5 Table) (Fig 3). Meanwhile, Germany also recorded the most substantial rise in the age-standardized DALYs rate for older adults with LOE, with an AAPC of 2.41% (S5 Table) (Fig 4). In 2021, Equatorial Guinea had the highest ASPR of LOE among older people (1,364.46 per 100,000 population), and Germany had the highest ASIR of LOE (97.31 per 100,000 population) (S5 Table). However, Zambia had the highest age standardized DALYs rate for LOE (1,059.20 per 100,000 population) and the highest ASMR (46.76 per 100,000 population) (S5 Table).

### Cross-country inequality analysis

Significant absolute and relative disparities in the burden of LOE, correlated with the SDI, were evident, with these inequalities widening substantially over time. The age-standardized DALYs rate was disproportionately concentrated in countries with lower sociodemographic development. The slope index of inequality revealed a gap of 306.12 DALYs per 100,000 population between countries at the lowest and highest ends of the SDI spectrum in 1990, narrowing to 224.06 DALYs per 100,000 population by 2021 (S12 Fig). Additionally, the concentration index, a measure of relative inequality, was estimated at −0.21 (95% CI: −0.26 to −0.16) in 1990, improving modestly to −0.14 (95% CI: −0.19 to −0.01) by 2021 (S13 Fig). The data highlight a persistent imbalance in the distribution of the LOE burden across nations with varying levels of sociodemographic development.

## Discussion

Globally, the past three decades have seen a substantial rise in the ASPR, ASIR, ASMR, and age-standardized DALYs rates of LOE among individuals aged 65 and over. LOE’s increasing medical and societal costs have turned it into a more significant public health problem. Our study investigates the global burden of LOE, with a special emphasis on trends among seniors (65 years and older) across all GBD regions and countries from 1990 to 2021. Our findings shed light on the changing burden of LOE over the last 30 years, focusing on regions and countries with diverse income levels. These results highlight an upward trend in LOE burden across certain regions and countries. A comprehensive understanding of these epidemiological patterns is essential for policymakers and healthcare practitioners to design and implement effective prevention and management strategies.

Between 1990 and 2021, the global ASPR and ASIR of LOE increased by 21.82% and 30.71%, respectively. In contrast, trends in ASMR and age-standardized DALYs rate associated with LOE in individuals aged 65 and older were less pronounced than those for ASPR and ASIR over the same period. With few exceptions, both the ASPR and ASIR of LOE have shown consistent increases across countries and regions with varying SDI levels. However, the ASMR and age-standardized DALY rate displayed more variable patterns, revealing notable disparities between countries and regions with differing sociodemographic profiles. The growing ASPR and ASIR of LOE among older adults are largely driven by potentially modifiable midlife risk factors [24]. Regionally, the highest ASPR, ASIR, ASMR, and age-standardized DALY rate were observed in high-SDI regions, followed by low-SDI regions, with the lowest rates recorded in high-middle SDI regions. This pattern highlights the persistent socioeconomic disparities in the global burden of LOE [25,26]. The highest ASIR among older adults was noted in Western Europe, Southern Sub-Saharan Africa, and Western Sub-Saharan Africa, where in Africa, this disparity is primarily attributed to the elevated incidence of HIV/AIDS and the overwhelming strain on fragile healthcare systems [27]. In wealthier nations, the incidence of epilepsy increases sharply after age 60 or 65, a trend not consistently observed in populations with lower socioeconomic status [28]. Factors such as high smoking rates, excessive alcohol consumption, and poor cardiovascular risk management may contribute to the high incidence rates in Western Europe.

Global disparities were evident among individuals aged 65 and older, with significant variations observed across different age groups. From 1990 to 2021, the ASIR of LOE exhibited a consistent upward trajectory across all age categories, with the most pronounced increase seen in those aged 80–89 years, particularly among women. Although new-onset idiopathic or unexplained (genetic) generalized epilepsy remains rare in older adults, factors such as chronic sleep deprivation can unmask an underlying lifelong predisposition to epilepsy [29]. As life expectancy increases, individuals with a history of idiopathic or unexplained generalized epilepsy, previously in long-term remission, may experience a relapse in later life. Additionally, physiological disturbances common in older adults can precipitate acute symptomatic seizures, which are defined as seizures occurring in close temporal association with a brain insult [30]. Compared to younger populations, research on epilepsy in older adults remains relatively underdeveloped. Key areas, such as drug trials, psychosocial assessments, and the evaluation of relevant comorbidities, require more comprehensive investigation in individuals aged 65 and older, who represent the largest cohort of epilepsy patients in both resource-rich and increasingly resource-poor regions.

When considering sex, women showed a more significant rise in the ASPR and ASIR of LOE than men. Throughout the study period, the disease burden, measured by DALYs, remained consistently higher in men, despite the contrasting trends. Men are generally more prone to episodes of excitability and epileptic seizures [31–33]. Nevertheless, social dynamics may affect this trend. In some places where epilepsy is heavily stigmatized, women might be more likely than men to conceal their epilepsy diagnosis. Sexual differences are apparent in numerous epilepsy and seizure disorders. The existing evidence indicates that differences in brain steroid hormones or neurosteroid levels between males and females may contribute to the sex differences in controlling seizures and experiencing epileptic seizures [31]. Due to the sex-specific disparities in LOE, there is a need for prevention strategies that are specifically designed for elderly women and men.

Evaluating cross-country differences in the LOE burden along the SDI gradient can provide crucial insights into the distribution of disease burden and identify nations where prevention and control strategies need to be improved. High-SDI countries usually offer their populations better healthcare access and more efficient health systems, which might contribute to a lower overall disease burden. In contrast, countries at lower levels of sociodemographic development exhibited a disproportionate concentration of LOE burden. This observation is consistent with prior research, which has shown that nations with lower SDI levels shoulder a disproportionately higher burden of LOE [15]. A study highlighted that older adults in Sub-Saharan Africa bear a greater disease burden and experience higher mortality rates compared to the global population [34]. The disproportionate burden of LOE in high SDI countries can largely be attributed to two primary factors. First, high SDI nations are characterized by aging populations and higher alcohol consumption, both of which are well-established risk factors for LOE, contributing to its rising incidence. Second, despite advancements in healthcare, no cure for LOE exists, even in developed countries, leading to a persistently elevated prevalence. The disproportionate burden of LOE in low SDI countries can be explained by three key factors. First, weaker healthcare systems and a high prevalence of infections that may precipitate epilepsy exacerbate this inequality. Second, there is a critical shortage of healthcare professionals trained to treat epilepsy, and those with adequate training are often unavailable in rural areas. Furthermore, individuals with epilepsy and their families frequently encounter stigma and discrimination, delaying the initiation of effective anti-seizure therapies. The WHO and the United Nations have increasingly emphasized the need for enhanced healthcare support and the development of medical guidelines in these countries, as part of a broader global call to action. Importantly, notable reductions in these disparities have been observed over time.

Cross-country variations in defining LOE could lead to differences in incidence and prevalence estimates. First, the age threshold defining “late onset” is not standardized across health systems: while some studies adopt 60 years, others use 65 or 70 years, which mechanically alters the denominators and shifts the age distribution of identified cases [28]. Second, there is variation in how faithfully ILAE criteria are implemented across different settings and whether acute symptomatic seizures are excluded; misclassification of these seizures can lead to exaggerated burden estimates [28]. Third, unequal access to diagnostic assessments like EEG and neuroimaging, as well as variations in coding standards and clinical evaluation, may additionally affect the identification of cases. These origins of diagnostic diversity have been recorded in numerous populations and could account for ongoing international discrepancies even after GBD unification. This study aims to characterize late-onset epilepsy in older adults; accordingly, we adopted a ≥ 65-year onset threshold, consistent with the clinically informed, data-driven definition proposed by Josephson et al. (2016) [4], which was derived using rigorous statistical methods. We also acknowledge that some studies have used alternative, and at times ambiguously defined, age cutoffs that are often guided by clinical convention or by national and global definitions of old age. Future research should focus on integrating harmonized multicenter registries with standardized ILAE protocols to mitigate these definitional inconsistencies and improve international comparability.

Acute symptomatic seizures and potential misclassification. In older adults, acute symptomatic seizures (ASyS) account for a large share of first-ever seizures and often occur in the context of acute brain insults such as stroke, metabolic derangements, or systemic illness [28,35,36]. Mistakenly categorizing ASyS as new epilepsy cases would cause an increase in the reported rates of LOE. Recent reports indicate that ASyS can account for up to 50% of new-onset seizures in older adults, with some patients progressing to epilepsy, complicating attribution in surveillance data [28,35,37]. These challenges persist even with harmonization efforts, and they underscore the need to adhere strictly to ILAE distinctions between unprovoked epileptic seizures and ASyS when defining LOE. This principle was followed in our study during the definition stage; nonetheless, we recognize the residual risk of misclassification due to variability in coding, diagnostic procedures, and data sources.

The study is also restricted by its lack of consideration for access to newer-generation antiseizure medications (ASMs). The GBD framework does not provide country-level information on ASM utilization, regulatory approval, or affordability. In regions with significant treatment gaps, such as low- and middle-income countries, these factors may greatly shape outcomes for older adults with LOE. Recent real-world studies have highlighted a rise in the use and effectiveness of third-generation ASMs, including lacosamide, brivaracetam, eslicarbazepine acetate, and perampanel, but access is still inconsistent across various health systems [38–40]. Reports from resource-limited areas repeatedly show that barriers in availability and affordability restrict adherence and clinical benefits [14,41]. Thus, part of the variation in LOE burden between countries may be explained by differences in drug availability rather than only by disease biology. Further studies should blend population-level modeling with registry or survey data on ASM access to more directly evaluate its effect on LOE outcomes.

This study faces limitations that are inherent to the GBD modeling framework. GBD incorporates a variety of sources, such as surveys, hospital records, and claims data, yet it does not provide direct clinical validation through multicenter epilepsy registries. Thus, our estimates should be viewed as indicators modeled at the population level, rather than outcomes confirmed by clinical assessment. The lack of direct validation may introduce risk of bias and constrain the applicability of our findings in specific clinical settings. To enhance diagnostic accuracy, validate case ascertainment, and strengthen the external validity of these estimates, future efforts must integrate multicenter clinical registries with GBD-based models.

An additional limitation involves the likelihood of epilepsy being underreported in regions with limited access to neurological care. The accurate diagnosis and monitoring in many low- and middle-income countries are hampered by a shortage of neurologists, restricted access to EEG and neuroimaging, and deficient health information systems. The underestimation of LOE in our study is probably influenced by these limitations. Despite the GBD framework’s use of modeling strategies to adjust for incomplete data, there may still be some residual diagnostic under-ascertainment. Recent evidence suggests that in resource-limited areas, there are still major gaps in epilepsy treatment and diagnosis, with as many as three-quarters of epilepsy sufferers possibly not receiving appropriate care or timely diagnosis [42,43]. Understanding this context helps explain some of the regional discrepancies we found and points to the need for strengthening health system capacity and registry infrastructure to secure more reliable burden estimates.

These sources of variation as a whole demonstrate the broader methodological constraints inherent to the GBD estimation framework. First, the analysis was exclusively based on modeled data from the GBD database, which gathers information from diverse sources but lacks direct clinical validation. Hence, our findings are modeled estimates for the population, rather than outcomes verified through assessments at the patient level. Second, the large 95% uncertainty intervals around some estimates highlight substantial statistical imprecision, which may impede the detection of significant cross-country differences, particularly in stable DALY trends. Third, the comorbidity adjustments used in the GBD framework assume independence between diseases and sequelae, whereas epilepsy frequently coexists with both somatic and psychiatric conditions [11,44] and is intertwined with social determinants such as stigma and poverty [13]. By simplifying, this assumption could lead to an underestimation of the real interactive effects of comorbidities. Fourth, dependence on administrative and claims data could lead to a bias in identifying cases, favoring treated populations and possibly overlooking those without formal healthcare or health insurance. These considerations highlight the necessity of integrating GBD modeling with clinical registries and health system data to boost diagnostic accuracy, refine comorbidity adjustments, and strengthen the external validity of future estimates.

## Conclusion

From 1990 to 2021, the global burden of LOE has shown a continuous upward trend, largely due to the increase in population and its aging. While countries with elevated SDI values continue to experience a disproportionate burden, the differences between nations have slowly reduced. These trends point to the dual challenge of overseeing a rising senior population and addressing continuous disparities in diagnostic and treatment capacities. Strengthening surveillance systems, improving access to antiseizure medications, and integrating epilepsy care into broader ageing and non-communicable disease frameworks will be essential for mitigating the future burden of LOE and promoting equity in neurological health worldwide.

## Supporting information

S1 Table APCC of ASIR, ASPR, ASMR, and age-standardized DALYs rate of LOE in individuals aged ≥65 years from 1990 to 2021 at Global by age subgroup. **Abbreviations:** ASIR, age-standardized incidence rate; ASPR, age-standardized prevalence rate; ASMR, age-standardized mortality rate; DALYs, disability-adjusted life-years; SDI, sociodemographic index; LOE, late-onset epilepsy; AAPC, average annual percent changes; CI, confidence interval. Numbers in parentheses are 95% uncertainty intervals (Cases and age standardized rate) and 95% confidence interval (AAPC).
(DOCX)

S2 Table ASPR and ASIR of LOE in individuals aged ≥65 years and their AAPCs from 1990 to 2021 at regional levels. **Abbreviations:** ASIR, age-standardized incidence rate; ASPR, age-standardized prevalence rate; ASMR, age-standardized mortality rate; DALYs, disability-adjusted life-years; SDI, sociodemographic index; AAPC, average annual percent changes; CI, confidence interval; P, P value for the significant test of AAPCs; LOE, late-onset epilepsy. Numbers in parentheses are 95% uncertainty intervals (Cases and age standardized rate) and 95% confidence interval (AAPC).
(DOCX)

S3 Table ASIR, ASPR, ASMR, and age-standardized DALYs rate of LOE in individuals aged ≥65 years in 2021 at regional levels by sex. **Abbreviations:** ASIR, age-standardized incidence rate; ASPR, age-standardized prevalence rate; ASMR, age-standardized mortality rate; AAPC, average annual percent changes; DALYs, disability-adjusted life years; CI, confidence interval; P, P value for the significant test of AAPC; LOE, late-onset epilepsy. Numbers in parentheses are 95% uncertainty intervals.
(DOCX)

S4 Table ASMR and age-standardized DALYs rate of LOE in individuals aged ≥65 years and their AAPCs from 1990 to 2021 at the regional levels. **Abbreviations:** ASIR, age-standardized incidence rate; ASPR, age-standardized prevalence rate; ASMR, age-standardized mortality rate; AAPC, average annual percent changes; DALYs, disability-adjusted life years; CI, confidence interval; P, P value for the significant test of AAPC; LOE, late-onset epilepsy. Numbers in parentheses are 95% uncertainty intervals.
(DOCX)

S5 Table ASIR, ASPR, ASMR, and age-standardized DALYs rate of LOE in individuals aged ≥65 years in 2021 and their AAPC between 1990–2021 in 204 countries and territories. **Abbreviations:** ASIR, age-standardized incidence rate; ASPR, age-standardized prevalence rate; ASMR, age-standardized mortality rate; AAPC, average annual percent changes; DALYs, disability-adjusted life years; CI, confidence interval; P, P value for the significant test of AAPC; LOE, late-onset epilepsy. Numbers in parentheses are 95% uncertainty intervals.
(DOCX)

S1 Fig Equations used to calculate age standardised rate and AAPC. **Abbreviations:** AAPC, average annual percentage change.
(PDF)

S2 Fig The changes in ASIR, ASPR, ASMR and age-standardized DALYs rate of LOE in individuals aged ≥65 years from 1990 to 2021. **Abbreviations:** ASIR, age-standardized incidence rate; ASPR, age-standardized prevalence rate; ASMR, age-standardized mortality rate; DALYs, disability-adjusted life-years; LOE, late-onset epilepsy.
(PDF)

S3 Fig Temporal trends in average annual percent change of ASIR, ASPR, ASMR, and age-standardized DALYs rate of aged 65 years and older and overall LOE patients from 1990 to 2021. **Abbreviations:** ASIR, age-standardized incidence rate; ASPR, age-standardized prevalence rate; ASMR, age-standardized mortality rate; DALYs, disability-adjusted life-years; SDI, sociodemographic index; LOE, late-onset epilepsy.
(PDF)

S4 Fig Temporal trend of ASIR, ASPR, ASMR, and age-standardized DALYs rate of LOE in individuals aged 65 years and older from 1990 to 2021 at global and SDI levels by sex. **Abbreviations:** ASIR, age-standardized incidence rate; ASPR, age-standardized prevalence rate; ASMR, age-standardized mortality rate; DALYs, disability-adjusted life-years; SDI, sociodemographic index; LOE, late-onset epilepsy.
(PDF)

S5 Fig Average annual percent change of ASIR, ASPR, ASMR, and age-standardized DALYs rate of LOE in individuals aged 65 years and older from 1990 to 2021 at SDI levels by sex. **Abbreviations:** ASIR, age-standardized incidence rate; ASPR, age-standardized prevalence rate; ASMR, age-standardized mortality rate; DALYs, disability-adjusted life-years; SDI, sociodemographic index; LOE, late-onset epilepsy.
(PDF)

S6 Fig Average annual percent change of ASIR, ASPR, ASMR, and age-standardized DALYs rate of LOE in individuals aged 65 years and older from 1990 to 2021 by sex and age. **Abbreviations:** ASIR, age-standardized incidence rate; ASPR, age-standardized prevalence rate; ASMR, age-standardized mortality rate; DALYs, disability-adjusted life-years; LOE, late-onset epilepsy.
(PDF)

S7 Fig Temporal trend of ASIR, ASPR, ASMR, and age-standardized DALYs rate of aged over 65 years and overall LOE patients from 1990 to 2021 at global and SDI levels. **Abbreviations:** ASIR, age-standardized incidence rate; ASPR, age-standardized prevalence rate; ASMR, age-standardized mortality rate; DALYs, disability-adjusted life-years; SDI, sociodemographic index; LOE, late-onset epilepsy.
(PDF)

S8 Fig Average annual percent change of ASIR, ASPR, ASMR, and age-standardized DALYs rate of aged over 65 years and overall LOE patients from 1990 to 2021 at global and SDI levels. **Abbreviations:** ASIR, age-standardized incidence rate; ASPR, age-standardized prevalence rate; ASMR, age-standardized mortality rate; DALYs, disability-adjusted life-years; SDI, sociodemographic index; LOE, late-onset epilepsy.
(PDF)

S9 Fig ASIR, ASPR, ASMR, and age-standardized DALYs rate of LOE in individuals of LOE aged 65 years and older from 204 countries according to the SDI in 2021. **Abbreviations:** ASIR, age-standardized incidence rate; ASPR, age-standardized prevalence rate; ASMR, age-standardized mortality rate; DALYs, disability-adjusted life-years; SDI, sociodemographic index; LOE, late-onset epilepsy.
(PDF)

S10 Fig Average annual percent change of ASIR, ASPR, ASMR, and age-standardized DALYs rate of LOE in individuals aged 65 years and older from 1990 to 2021 at regions levels. **Abbreviations:** ASIR, age-standardized incidence rate; ASPR, age-standardized prevalence rate; ASMR, age-standardized mortality rate; DALYs, disability-adjusted life-years; LOE, late-onset epilepsy.
(PDF)

S11 Fig Average annual percent change of ASIR, ASPR, ASMR, and age-standardized DALYs rate of LOE in individuals aged 65 years and older from 1990 to 2021 at regions levels by sex. **Abbreviations:** ASIR, age-standardized incidence rate; ASPR, age-standardized prevalence rate; ASMR, age-standardized mortality rate; DALYs, disability-adjusted life-years; LOE, late-onset epilepsy.
(PDF)

S12 Fig SDI-related health inequality regression curves for the age-standardized DALYs rate of LOE worldwide, 1990 and 2021. **Abbreviations:** SDI, sociodemographic index; DALYs, disability-adjusted life-years; LOE, late-onset epilepsy.
(PDF)

S13 Fig SDI-related health inequality concentration curves for the age-standardized DALYs rate of LOE worldwide, 1990 and 2021. **Abbreviations:** SDI, sociodemographic index; DALYs, disability-adjusted life-years; LOE, late-onset epilepsy.
(PDF)
