# Supplementary material for: Global, regional, and national burdens of late-onset epilepsy in adults aged 65 years and older from 1990 to 2021: A population-based study
Source: PLoS One. 2025 Nov 19;20(11):e0336588. doi: 10.1371/journal.pone.0336588 (PMC12629476; doi:10.1371/journal.pone.0336588)
Supplement: S1 Table — Abbreviations: ASIR, age-standardized incidence rate; ASPR, age-standardized prevalence rate; ASMR, age-standardized mortality rate; DALYs, disability-adjusted life-years; SDI, sociodemographic index; LOE, late-onset epilepsy; AAPC, average annual percent changes; CI, confidence interval. Numbers in parentheses are 95% uncertainty intervals (Cases and age standardized rate) and 95% confidence interval (AAPC). (DOCX) [file pone.0336588.s001.docx]

**S1 Table** APCC of ASIR, ASPR, ASMR, and age-standardized DALYs rate of LOE in individuals aged ≥65 years from 1990 to 2021 at Global by age subgroup

**Abbreviations:** ASIR, age-standardized incidence rate; ASPR, age-standardized prevalence rate; ASMR, age-standardized mortality rate; DALYs, disability-adjusted life-years; SDI, sociodemographic index; LOE, late-onset epilepsy; AAPC, average annual percent changes; CI, confidence interval. Numbers in parentheses are 95% uncertainty intervals (Cases and age standardized rate) and 95% confidence interval (AAPC).

| **Age group (years)** | **AAPC (95% CI) 1990-2021 (%/year)** | | | |
| --- | --- | --- | --- | --- |
|  | **Age-standardized incidence rate** | **Age-standardized prevalence rate** | **Age-standardized mortality rate** | **Age-standardized DALYs rate** |
|  | **Both** | | | |
| 65 to 69 | 0.65 (0.58 to 0.71) | 0.46 (0.40 to 0.52) | -0.37 (-0.58 to -0.16) | -0.18 (-0.25 to -0.10) |
| 70 to 74 | 0.79 (0.74 to 0.84) | 0.59 (0.53 to 0.65) | -0.35 (-0.58 to -0.11) | -0.12 (-0.28 to 0.03) |
| 75 to 79 | 0.95 (0.91 to 0.99) | 0.68 (0.65 to 0.72) | 0.17 (-0.04 to 0.38) | 0.19 (0.14 to 0.23) |
| 80 to 84 | 1.01 (0.91 to 1.10) | 0.80 (0.76 to 0.84) | 0.72 (0.23 to 1.21) | 0.41 (0.27 to 0.56) |
| 85 to 89 | 1.01 (0.93 to 1.08) | 0.88 (0.78 to 0.98) | 1.04 (0.64 to 1.44) | 0.59 (0.48 to 0.70) |
| 90 to 94 | 0.79 (0.72 to 0.86) | 0.90 (0.76 to 1.04) | 1.58 (1.06 to 2.10) | 0.85 (0.74 to 0.96) |
| ≥95 | 0.64(0.6 to 0.69) | 0.87 (0.76 to 0.97) | 1.38 (0.94 to 1.83) | 0.78 (0.62 to 0.94) |
|  | **Male** | | | |
| 65 to 69 | 0.57 (0.51 to 0.64) | 0.43 (0.38 to 0.49) | -0.58 (-0.83 to -0.33) | -0.31 (-0.34 to -0.28) |
| 70 to 74 | 0.69 (0.60 to 0.78) | 0.56 (0.49 to 0.62) | -0.81 (-1.15 to -0.46) | -0.42 (-0.59 to -0.25) |
| 75 to 79 | 0.71 (0.63 to 0.79) | 0.57 (0.54 to 0.60) | -0.43 (-0.6 to -0.26) | -0.15 (-0.24 to -0.06) |
| 80 to 84 | 0.73 (0.65 to 0.82) | 0.66 (0.61 to 0.72) | -0.02 (-0.56 to 0.52) | 0.09 (-0.07 to 0.25) |
| 85 to 89 | 0.72 (0.66 to 0.78) | 0.71 (0.64 to 0.79) | 0.34 (0.09 to 0.59) | 0.21 (0.08 to 0.34) |
| 90 to 94 | 0.62 (0.57 to 0.66) | 0.72 (0.56 to 0.89) | 0.65 (0.32 to 0.97) | 0.35 (0.19 to 0.51) |
| ≥95 | 0.59 (0.53 to 0.64) | 0.75 (0.67 to 0.82) | 0.26 (-0.11 to 0.63) | 0.21 (-0.02 to 0.43) |
|  | **Female** | | | |
| 65 to 69 | 0.71 (0.59 to 0.83) | 0.48 (0.44 to 0.51) | -0.09 (-0.33 to 0.16) | -0.05 (-0.13 to 0.03) |
| 70 to 74 | 0.87 (0.83 to 0.92) | 0.61 (0.56 to 0.65) | 0.15 (-0.10 to 0.41) | 0.06 (-0.13 to 0.25) |
| 75 to 79 | 1.11 (1.06 to 1.16) | 0.74 (0.67 to 0.81) | 0.80 (0.53 to 1.07) | 0.45 (0.35 to 0.55) |
| 80 to 84 | 1.16 (1.00 to 1.32) | 0.85 (0.81 to 0.90) | 1.32 (0.79 to 1.85) | 0.68 (0.55 to 0.81) |
| 85 to 89 | 1.14 (1.06 to 1.22) | 0.95 (0.85 to 1.05) | 1.57 (1.11 to 2.04) | 0.82 (0.68 to 0.96) |
| 90 to 94 | 0.86 (0.77 to 0.94) | 0.96 (0.85 to 1.08) | 2.19 (1.8 to 2.59) | 1.10 (0.97 to 1.23) |
| ≥95 | 0.66 (0.61 to 0.71) | 0.92 (0.85 to 1.00) | 1.92 (1.47 to 2.38) | 1.01 (0.83 to 1.18) |
