# Supplementary material for: Global, regional, and national burdens of late-onset epilepsy in adults aged 65 years and older from 1990 to 2021: A population-based study
Source: PLoS One. 2025 Nov 19;20(11):e0336588. doi: 10.1371/journal.pone.0336588 (PMC12629476; doi:10.1371/journal.pone.0336588)
Supplement: S2 Table — Abbreviations: ASIR, age-standardized incidence rate; ASPR, age-standardized prevalence rate; ASMR, age-standardized mortality rate; DALYs, disability-adjusted life-years; SDI, sociodemographic index; AAPC, average annual percent changes; CI, confidence interval; P, P value for the significant test of AAPCs; LOE, late-onset epilepsy. Numbers in parentheses are 95% uncertainty intervals (Cases and age standardized rate) and 95% confidence interval (AAPC). (DOCX) [file pone.0336588.s002.docx]

**S2 Table** ASPR and ASIR of LOE in individuals aged ≥65 years and their AAPCs from 1990 to 2021 at regional levels

**Abbreviations: Abbreviations:** ASIR, age-standardized incidence rate; ASPR, age-standardized prevalence rate; ASMR, age-standardized mortality rate; DALYs, disability-adjusted life-years; SDI, sociodemographic index; AAPC, average annual percent changes; CI, confidence interval; P, P value for the significant test of AAPCs; LOE, late-onset epilepsy. Numbers in parentheses are 95% uncertainty intervals (Cases and age standardized rate) and 95% confidence interval (AAPC).

| **Regions** | **No of people with**  **LOE in 1990** | **Age standardised rate in 1990 (per 100 000)** | **No of people with**  **LOE in 2021** | **Age-standardized rate in 2021 (per 100,000)** | **AAPCs in rate,**  **1990- 2021 (%/year)** | **P value** |
| --- | --- | --- | --- | --- | --- | --- |
|  | **Prevalence (95% UI)** | | | | | |
| Andean Latin America | 11387.92 (5282.49 to 17707.88) | 718.49 (332.81 to 1119.60) | 38883.13 (20145.29 to 60732.14) | 778.66 (403.04 to 1215.63) | 0.22 (0.09 to 0.35) | 0.001 |
| Australasia | 9240.05 (3401.27 to 15312.41) | 420.38 (154.85 to 696.07) | 23581.64 (8803.79 to 39050.23) | 446.00 (166.66 to 738.83) | 0.19 (0.17 to 0.21) | <0.001 |
| Caribbean | 10376.68 (6183.22 to 15054.11) | 468.86 (279.11 to 680.21) | 22991.00 (13862.13 to 33533.15) | 480.59 (290.23 to 700.32) | 0.09 (0.07 to 0.10) | <0.001 |
| Central Asia | 14732.99 (8692.72 to 21554.69) | 421.95 (248.78 to 617.81) | 32441.02 (18401.56 to 46421.76) | 537.51 (306.18 to 766.77) | 0.77 (0.73 to 0.81) | <0.001 |
| Central Europe | 52696.88 (34829.99 to 74085.60) | 398.27 (263.50 to 558.39) | 102916.06 (66985.51 to 144173.00) | 460.82 (299.89 to 645.45) | 0.47 (0.43 to 0.5) | <0.001 |
| Central Latin America | 46414.85 (30323.39 to 66262.24) | 735.66 (480.52 to 1049.82) | 160234.04 (108389.82 to 229532.71) | 759.94 (514.07 to 1086.71) | 0.11 (0.08 to 0.14) | <0.001 |
| Central Sub-Saharan Africa | 9511.87 (3414.47 to 16847.08) | 670.25 (240.81 to 1186.95) | 21502.94 (9470.01 to 35195.18) | 648.91 (283.79 to 1064.25) | -0.13 (-0.22 to -0.04) | 0.004 |
| East Asia | 176556.72 (115032.96 to 258689.11) | 279.93 (182.22 to 409.79) | 656959.20 (435225.15 to 934762.36) | 336.51 (222.53 to 478.00) | 0.65 (0.37 to 0.92) | <0.001 |
| Eastern Europe | 67951.49 (45606.78 to 96242.18) | 291.77 (195.37 to 413.62) | 103665.56 (67645.58 to 149528.55) | 311.08 (202.91 to 448.81) | 0.20 (0.13 to 0.26) | <0.001 |
| Eastern Sub-Saharan Africa | 24741.85 (14078.65 to 39167.28) | 497.61 (281.89 to 786.98) | 60307.19 (38511.79 to 86409.93) | 547.63 (347.58 to 784.59) | 0.31 (0.19 to 0.43) | <0.001 |
| High-income Asia Pacific | 61191.53 (39227.05 to 88851.27) | 361.45 (231.24 to 525.04) | 222835.31 (132630.44 to 323607.56) | 451.02 (267.58 to 657.03) | 0.72 (0.65 to 0.79) | <0.001 |
| High-income North America | 114697.35 (76379.31 to 163314.87) | 334.89 (222.77 to 477.30) | 344773.03 (209241.62 to 498219.26) | 534.28 (324.79 to 771.47) | 1.52 (1.29 to 1.75) | <0.001 |
| North Africa and Middle East | 37588.68 (23246.14 to 55621.78) | 322.09 (197.92 to 474.56) | 134059.93 (81763.62 to 193189.00) | 413.93 (251.81 to 595.56) | 0.82 (0.77 to 0.87) | <0.001 |
| Oceania | 624.19 (296.45 to 1047.14) | 341.8 (165.09 to 566.57) | 1565.48 (771.52 to 2500.88) | 338.74 (169.75 to 537.94) | -0.03 (-0.07 to 0.00) | 0.081 |
| South Asia | 146935.25 (86250.2 to 227888.4) | 387.09 (227.32 to 600.04) | 490468.82 (337634.27 to 692901.55) | 434.22 (298.54 to 612.52) | 0.34 (0.22 to 0.47) | <0.001 |
| Southeast Asia | 54270.48 (35679.41 to 80237.78) | 307.71 (201.86 to 454.77) | 213937.75 (133614.6 to 307819.35) | 440.83 (273.70 to 634.76) | 1.15 (1.11 to 1.19) | <0.001 |
| Southern Latin America | 13298.56 (6333.07 to 21179.47) | 330.14 (157.19 to 525.40) | 34638.16 (17376.18 to 54826.06) | 425.41 (213.35 to 673.62) | 0.83 (0.81 to 0.85) | <0.001 |
| Southern Sub-Saharan Africa | 12401.84 (7586.16 to 18168.38) | 608.67 (370.85 to 888.39) | 28972.23 (18479.84 to 42484.91) | 683.36 (434.66 to 1001.85) | 0.36 (0.28 to 0.43) | <0.001 |
| Tropical Latin America | 41796.57 (25986.65 to 60497.27) | 599.79 (372.83 to 867.07) | 145014.75 (88200.97 to 213539.23) | 657.2 (399.94 to 966.38) | 0.30 (0.27 to 0.33) | <0.001 |
| Western Europe | 280162.47 (177612.57 to 395144.17) | 502.37 (318.26 to 709.55) | 650440.62 (387498.82 to 914889.15) | 685.50 (408.97 to 968.74) | 1.01 (0.97 to 1.05) | <0.001 |
| Western Sub-Saharan Africa | 34192.51 (20392.28 to 52423.88) | 543.01 (321.94 to 834.16) | 83338.82 (56659.95 to 118104) | 646.49 (437.81 to 916.22) | 0.57 (0.55 to 0.60) | <0.001 |
|  | **Incidence (95% UI)** | | | | | |
| Andean Latin America | 624.29 (251.93 to 1154.26) | 39.74 (15.98 to 73.84) | 2331.13 (1073.70 to 3961.30) | 46.80 (21.55 to 79.62) | 0.50 (0.40 to 0.59) | <0.001 |
| Australasia | 612.80 (205.02 to 1111.45) | 28.17 (9.40 to 51.40) | 1652.16 (540.03 to 3086.34) | 31.06 (10.19 to 57.94) | 0.30 (0.22 to 0.38) | <0.001 |
| Caribbean | 633.74 (317.15 to 1022.22) | 29.11 (14.48 to 47.43) | 1479.44 (757.59 to 2468.76) | 30.82 (15.87 to 51.12) | 0.18 (0.17 to 0.19) | <0.001 |
| Central Asia | 601.53 (299.33 to 1028.04) | 17.34 (8.58 to 29.80) | 1367.47 (677.01 to 2256.90) | 22.22 (10.84 to 37.07) | 0.80 (0.78 to 0.83) | <0.001 |
| Central Europe | 1923.88 (1007.11 to 3010.01) | 14.49 (7.49 to 22.95) | 3851.03 (1973.48 to 6140.49) | 17.25 (8.84 to 27.51) | 0.56 (0.49 to 0.64) | <0.001 |
| Central Latin America | 2763.98 (1511.57 to 4353.95) | 44.11 (23.91 to 70.37) | 9739.20 (5347.10 to 15527.18) | 46.12 (25.34 to 73.50) | 0.15 (0.11 to 0.19) | 0.686 |
| Central Sub-Saharan Africa | 636.25 (192.02 to 1247.42) | 45.50 (14.00 to 89.45) | 1482.55 (548.51 to 2715.52) | 45.58 (16.90 to 84.02) | -0.01 (-0.08 to 0.05) | <0.001 |
| East Asia | 11838.60 (6239.43 to 19155.98) | 18.69 (9.71 to 30.88) | 43722.40 (23112.86 to 69971.02) | 22.49 (11.78 to 36.46) | 0.75 (0.54 to 0.97) | <0.001 |
| Eastern Europe | 3086.08 (1563.33 to 5024.28) | 13.45 (6.77 to 22.17) | 5468.28 (2847.98 to 8809.97) | 16.35 (8.49 to 26.46) | 0.62 (0.56 to 0.67) | <0.001 |
| Eastern Sub-Saharan Africa | 1821.54 (898.50 to 3175.39) | 40.61 (19.93 to 71.71) | 4670.72 (2600.98 to 7359.29) | 46.66 (25.85 to 74.49) | 0.43 (0.40 to 0.46) | <0.001 |
| High-income Asia Pacific | 4291.29 (2258.05 to 6953.02) | 25.75 (13.42 to 42.30) | 17429.54 (8653.56 to 29196.88) | 34.91 (17.56 to 57.27) | 0.98 (0.90 to 1.05) | <0.001 |
| High-income North America | 7757.73 (4149.34 to 12673.43) | 22.73 (12.12 to 37.26) | 24033.94 (11890.45 to 39410.14) | 37.13 (18.44 to 60.62) | 1.61 (1.48 to 1.75) | <0.001 |
| North Africa and Middle East | 2607.40 (1339.70 to 4224.37) | 22.96 (11.74 to 37.97) | 9846.66 (5094.19 to 15775.89) | 30.98 (15.97 to 50.26) | 0.95 (0.88 to 1.03) | <0.001 |
| Oceania | 38.60 (15.43 to 71.34) | 21.35 (8.72 to 39.45) | 98.97 (43.60 to 174.83) | 21.53 (9.58 to 38.17) | 0.03 (0.00 to 0.05) | 0.052 |
| South Asia | 11745.47 (5869.80 to 20180.48) | 31.37 (15.68 to 54.68) | 40629.07 (22863.13 to 62334.40) | 36.47 (20.37 to 56.80) | 0.47 (0.38 to 0.56) | <0.001 |
| Southeast Asia | 3646.97 (1970.78 to 5990.44) | 20.83 (11.16 to 34.86) | 14777.73 (7781.09 to 23822.45) | 30.51 (15.91 to 49.91) | 1.23 (1.18 to 1.28) | <0.001 |
| Southern Latin America | 818.40 (330.07 to 1457.89) | 20.59 (8.29 to 36.94) | 2225.99 (970.38 to 3875.35) | 27.32 (11.90 to 47.50) | 0.92 (0.89 to 0.94) | <0.001 |
| Southern Sub-Saharan Africa | 853.75 (450.04 to 1389.84) | 42.48 (22.13 to 70.29) | 2123.94 (1093.15 to 3385.99) | 51.12 (26.06 to 83.49) | 0.58 (0.51 to 0.64) | <0.001 |
| Tropical Latin America | 2320.31 (1140.19 to 3832.21) | 33.77 (16.41 to 57.04) | 9188.34 (4524.19 to 15270.62) | 41.64 (20.52 to 69.29) | 0.68 (0.65 to 0.71) | <0.001 |
| Western Europe | 20465.48 (11471.55 to 31849.87) | 36.94 (20.55 to 57.84) | 52846.74 (28304.99 to 79267.23) | 55.42 (29.93 to 82.84) | 1.32 (1.27 to 1.38) | <0.001 |
| Western Sub-Saharan Africa | 2454.39 (1221.99 to 4193.76) | 41.67 (20.57 to 73.33) | 6131.37 (3374.22 to 9609.20) | 50.50 (27.56 to 80.80) | 0.63 (0.58 to 0.67) | <0.001 |
