# Supplementary material for: Global, regional, and national burdens of late-onset epilepsy in adults aged 65 years and older from 1990 to 2021: A population-based study
Source: PLoS One. 2025 Nov 19;20(11):e0336588. doi: 10.1371/journal.pone.0336588 (PMC12629476; doi:10.1371/journal.pone.0336588)
Supplement: S3 Table — Abbreviations: ASIR, age-standardized incidence rate; ASPR, age-standardized prevalence rate; ASMR, age-standardized mortality rate; AAPC, average annual percent changes; DALYs, disability-adjusted life years; CI, confidence interval; P, P value for the significant test of AAPC; LOE, late-onset epilepsy. Numbers in parentheses are 95% uncertainty intervals. (DOCX) [file pone.0336588.s003.docx]

**S3 Table**. ASIR, ASPR, ASMR, and age-standardized DALYs rate of LOE in individuals aged ≥65 years in 2021 at regional levels by sex

**Abbreviations:** ASIR, age-standardized incidence rate; ASPR, age-standardized prevalence rate; ASMR, age-standardized mortality rate; AAPC, average annual percent changes; DALYs, disability-adjusted life years; CI, confidence interval; P, P value for the significant test of AAPC; LOE, late-onset epilepsy. Numbers in parentheses are 95% uncertainty intervals.

| **Regions** | **Age-standardized rate in 2021 (per 100,000)** | | | | | | | |
| --- | --- | --- | --- | --- | --- | --- | --- | --- |
|  | **Male** | | | | **Female** | | | |
|  | **Incidence**  **(95% UI)** | **Prevalence**  **(95% UI)** | **Mortality**  **(95% UI)** | **DALYs**  **(95% UI)** | **Incidence**  **(95% UI)** | **Prevalence**  **(95% UI)** | **Mortality**  **(95% UI)** | **DALYs**  **(95% UI)** |
| Andean Latin America | 46.27 (20.52 to 80.29) | 743.98 (382.46 to 1169.43) | 3.45 (2.57 to 4.43) | 256.16 (144.76 to 424.00) | 47.25 (21.81 to 81.27) | 809.56 (415.66 to 1265.36) | 2.41 (1.68 to 3.24) | 253.86 (133.22 to 428.69) |
| Australasia | 34.98 (11.77 to 65.54) | 486.03 (180.63 to 816.80) | 2.73 (2.25 to 3.21) | 135.75 (64.27 to 264.60) | 27.71 (8.85 to 52.41) | 412.40 (154.33 to 681.88) | 2.10 (1.64 to 2.56) | 111.41 (50.68 to 225.44) |
| Caribbean | 33.55 (17.65 to 55.15) | 507.65 (307.17 to 741.47) | 5.30 (4.29 to 6.52) | 225.91 (154.92 to 316.84) | 28.56 (14.51 to 47.89) | 458.24 (274.63 to 671.61) | 2.88 (2.30 to 3.54) | 167.72 (106.40 to 253.77) |
| Central Asia | 27.12 (13.21 to 45.31) | 622.80 (357.15 to 894.59) | 3.80 (3.26 to 4.38) | 237.55 (152.48 to 353.19) | 19.20 (9.01 to 32.10) | 481.66 (271.52 to 695.88) | 1.89 (1.58 to 2.19) | 162.48 (98.04 to 250.36) |
| Central Europe | 19.62 (10.04 to 31.51) | 537.42 (348.44 to 750.50) | 5.49 (4.66 to 6.16) | 218.19 (159.61 to 304.43) | 15.67 (7.94 to 25.19) | 406.99 (263.91 to 574.52) | 3.23 (2.59 to 3.71) | 141.61 (96.87 to 209.63) |
| Central Latin America | 49.16 (27.12 to 78.53) | 763.31 (517.50 to 1092.66) | 5.22 (4.55 to 5.92) | 289.56 (201.24 to 413.95) | 43.72 (23.70 to 70.65) | 758.42 (509.25 to 1085.78) | 3.57 (3.05 to 4.09) | 257.73 (171.11 to 385.86) |
| Central Sub-Saharan Africa | 62.31 (23.61 to 113.35) | 840.92 (369.25 to 1372.84) | 14.74 (9.63 to 22.26) | 527.79 (325.44 to 795.23) | 34.78 (12.40 to 65.40) | 521.51 (223.92 to 864.21) | 3.75 (1.22 to 7.29) | 236.37 (109.52 to 393.05) |
| East Asia | 23.90 (12.58 to 38.29) | 356.18 (235.70 to 502.22) | 1.79 (1.31 to 2.37) | 113.09 (71.67 to 172.82) | 21.14 (10.98 to 34.66) | 320.32 (212.12 to 458.21) | 1.02 (0.72 to 1.46) | 92.01 (55.11 to 144.49) |
| Eastern Europe | 20.17 (10.56 to 32.50) | 380.14 (249.35 to 545.44) | 1.64 (1.41 to 1.92) | 114.25 (72.00 to 175.53) | 14.40 (7.44 to 23.49) | 276.48 (179.33 to 400.22) | 1.29 (1.09 to 1.45) | 80.26 (49.34 to 125.75) |
| Eastern Sub-Saharan Africa | 55.18 (29.92 to 88.46) | 630.16 (394.94 to 908.73) | 61.32 (46.46 to 77.63) | 1141.31 (885.41 to 1422.69) | 38.96 (21.26 to 63.47) | 477.64 (302.26 to 695.43) | 16.05 (11.74 to 22.97) | 457.69 (346.54 to 611.82) |
| High-income Asia Pacific | 41.27 (20.90 to 67.46) | 521.69 (307.75 to 758.21) | 3.56 (3.04 to 3.98) | 157.64 (98.39 to 250.26) | 29.74 (14.80 to 49.60) | 396.51 (236.07 to 580.64) | 1.80 (1.31 to 2.20) | 104.26 (59.52 to 176.29) |
| High-income North America | 39.71 (19.89 to 64.91) | 567.05 (345.07 to 816.61) | 2.53 (2.25 to 2.73) | 154.51 (93.48 to 243.67) | 34.87 (17.14 to 57.31) | 508.32 (309.56 to 734.90) | 2.48 (2.09 to 2.74) | 141.17 (87.12 to 221.49) |
| North Africa and Middle East | 31.50 (16.66 to 50.48) | 422.43 (260.49 to 609.06) | 4.92 (3.64 to 7.12) | 183.28 (127.86 to 263.24) | 30.43 (15.33 to 50.27) | 405.23 (242.43 to 589.26) | 3.30 (2.16 to 4.51) | 149.94 (97.56 to 222.51) |
| Oceania | 20.04 (8.58 to 36.12) | 337.52 (164.48 to 547.50) | 2.52 (1.59 to 4.05) | 149.86 (76.15 to 251.31) | 22.77 (10.06 to 40.61) | 337.00 (172.24 to 531.13) | 1.08 (0.57 to 1.88) | 122.68 (59.36 to 213.65) |
| South Asia | 35.81 (20.01 to 55.49) | 432.95 (297.98 to 609.29) | 8.79 (6.26 to 10.85) | 261.67 (193.75 to 341.47) | 36.96 (20.57 to 57.88) | 435.18 (298.39 to 616.73) | 11.14 (5.22 to 14.10) | 298.68 (190.76 to 385.49) |
| Southeast Asia | 30.32 (15.84 to 49.51) | 428.61 (265.58 to 614.04) | 3.29 (2.30 to 4.31) | 174.21 (114.40 to 256.84) | 30.80 (15.98 to 50.66) | 450.16 (279.28 to 650.47) | 1.41 (0.95 to 2.04) | 147.18 (85.64 to 233.24) |
| Southern Latin America | 32.55 (14.42 to 56.72) | 488.70 (240.73 to 775.89) | 3.42 (2.96 to 3.91) | 172.22 (99.45 to 272.37) | 23.47 (9.98 to 41.57) | 380.96 (190.95 to 602.16) | 2.38 (1.98 to 2.76) | 127.00 (71.64 to 209.91) |
| Southern Sub-Saharan Africa | 53.39 (27.78 to 86.57) | 722.56 (457.80 to 1054.68) | 6.92 (4.82 to 8.60) | 334.86 (228.22 to 468.47) | 49.85 (25.10 to 81.91) | 659.12 (418.30 to 967.85) | 3.61 (2.23 to 4.70) | 249.14 (159.29 to 370.39) |
| Tropical Latin America | 46.92 (23.29 to 78.14) | 723.17 (440.61 to 1061.02) | 5.61 (5.00 to 6.13) | 283.11 (190.14 to 419.25) | 37.84 (18.54 to 63.25) | 608.60 (370.43 to 900.68) | 3.59 (3.02 to 4.01) | 215.23 (137.50 to 328.29) |
| Western Europe | 65.64 (36.54 to 96.39) | 795.46 (472.08 to 1104.02) | 7.20 (6.23 to 7.93) | 266.31 (179.00 to 409.56) | 47.14 (24.34 to 72.35) | 599.71 (359.21 to 868.93) | 6.32 (5.05 to 7.25) | 207.41 (141.06 to 317.52) |
| Western Sub-Saharan Africa | 48.01 (26.67 to 76.42) | 660.56 (443.99 to 931.66) | 17.98 (11.23 to 22.45) | 590.33 (406.66 to 758.07) | 51.20 (27.03 to 83.87) | 629.49 (424.41 to 900.49) | 5.87 (3.52 to 7.59) | 276.94 (182.05 to 395.50) |
