# Supplementary material for: Global, regional, and national burdens of late-onset epilepsy in adults aged 65 years and older from 1990 to 2021: A population-based study
Source: PLoS One. 2025 Nov 19;20(11):e0336588. doi: 10.1371/journal.pone.0336588 (PMC12629476; doi:10.1371/journal.pone.0336588)
Supplement: S4 Table — Abbreviations: ASIR, age-standardized incidence rate; ASPR, age-standardized prevalence rate; ASMR, age-standardized mortality rate; AAPC, average annual percent changes; DALYs, disability-adjusted life years; CI, confidence interval; P, P value for the significant test of AAPC; LOE, late-onset epilepsy. Numbers in parentheses are 95% uncertainty intervals. (DOCX) [file pone.0336588.s004.docx]

**S4 Table.** ASMR and age-standardized DALYs rate of LOE in individuals aged ≥65 years and their AAPCs from 1990 to 2021 at the regional levels

**Abbreviations:** ASIR, age-standardized incidence rate; ASPR, age-standardized prevalence rate; ASMR, age-standardized mortality rate; AAPC, average annual percent changes; DALYs, disability-adjusted life years; CI, confidence interval; P, P value for the significant test of AAPC; LOE, late-onset epilepsy. Numbers in parentheses are 95% uncertainty intervals.

| **Regions** | **No of people with**  **LOE in 1990** | **Age standardised rate in 1990 (per 100 000)** | **No of people with**  **LOE in 2021** | **Age-standardized rate in 2021 (per 100,000)** | **AAPCs in rate,**  **1990- 2021 (%/year)** | **P value** |
| --- | --- | --- | --- | --- | --- | --- |
|  | **Mortality (95% UI)** | | | | | |
| Andean Latin America | 70.77 (57.27 to 84.79) | 4.54 (3.68 to 5.45) | 143.78 (112.46 to 178.30) | 2.90 (2.27 to 3.59) | -1.47 (-2.23 to -0.71) | <0.001 |
| Australasia | 57.18 (50.18 to 63.94) | 2.67 (2.33 to 3.00) | 130.69 (108.44 to 150.21) | 2.38 (1.98 to 2.73) | -0.34 (-0.70 to 0.02) | 0.062 |
| Caribbean | 114.77 (101.18 to 131.69) | 5.34 (4.70 to 6.11) | 190.65 (159.35 to 226.10) | 3.98 (3.33 to 4.72) | -0.83 (-1.14 to -0.52) | <0.001 |
| Central Asia | 64.14 (57.95 to 73.91) | 1.87 (1.69 to 2.16) | 152.79 (131.53 to 172.13) | 2.62 (2.25 to 2.96) | 0.92 (0.38 to 1.47) | 0.001 |
| Central Europe | 290.38 (267.23 to 318.03) | 2.33 (2.13 to 2.57) | 933.32 (779.95 to 1046.64) | 4.20 (3.50 to 4.71) | 1.96 (1.38 to 2.55) | <0.001 |
| Central Latin America | 312.96 (293.19 to 329.86) | 5.23 (4.87 to 5.52) | 897.23 (793.49 to 998.14) | 4.31 (3.81 to 4.79) | -0.69 (-1.15 to -0.23) | 0.003 |
| Central Sub-Saharan Africa | 131.42 (89.28 to 189.30) | 10.39 (7.01 to 15.12) | 250.18 (163.42 to 369.01) | 8.02 (5.19 to 11.91) | -0.82 (-0.89 to -0.76) | <0.001 |
| East Asia | 1403.51 (1163.16 to 1902.97) | 2.36 (1.95 to 3.23) | 2478.73 (1956.71 to 3156.12) | 1.34 (1.05 to 1.70) | -1.81 (-2.06 to -1.56) | <0.001 |
| Eastern Europe | 222.72 (211.32 to 231.79) | 1.07 (1.01 to 1.12) | 451.82 (390.55 to 505.15) | 1.44 (1.25 to 1.61) | 1.22 (0.37 to 2.09) | 0.005 |
| Eastern Sub-Saharan Africa | 2265.11 (1766.79 to 2776.17) | 48.29 (37.32 to 59.93) | 3741.34 (3032.51 to 4609.25) | 35.61 (28.62 to 44.06) | -0.97 (-1.03 to -0.91) | <0.001 |
| High-income Asia Pacific | 139.51 (117.49 to 182.17) | 0.86 (0.72 to 1.11) | 1467.12 (1154.40 to 1694.82) | 2.55 (2.06 to 2.92) | 3.67 (2.69 to 4.66) | <0.001 |
| High-income North America | 560.21 (508.12 to 593.01) | 1.63 (1.48 to 1.73) | 1623.26 (1410.15 to 1763.85) | 2.50 (2.18 to 2.72) | 1.40 (0.99 to 1.82) | <0.001 |
| North Africa and Middle East | 573.21 (448.13 to 835.16) | 5.46 (4.19 to 8.12) | 1221.31 (960.65 to 1569.94) | 4.09 (3.20 to 5.37) | -0.90 (-1.13 to -0.67) | <0.001 |
| Oceania | 2.56 (1.52 to 4.42) | 1.82 (1.07 to 3.13) | 7.26 (4.46 to 11.74) | 1.82 (1.11 to 2.94) | 0.00 (-0.04 to 0.04) | 0.941 |
| South Asia | 4044.43 (2412.21 to 5067.77) | 11.41 (6.73 to 14.47) | 10480.30 (6785.06 to 12386.40) | 10.03 (6.41 to 11.95) | -0.32 (-0.85 to 0.21) | 0.233 |
| Southeast Asia | 423.47 (322.22 to 532.20) | 2.60 (1.98 to 3.30) | 975.51 (732.02 to 1239.56) | 2.20 (1.66 to 2.82) | -0.53 (-0.72 to -0.34) | <0.001 |
| Southern Latin America | 83.85 (74.50 to 93.70) | 2.16 (1.91 to 2.42) | 231.17 (199.21 to 261.43) | 2.82 (2.43 to 3.19) | 0.92 (0.16 to 1.68) | 0.018 |
| Southern Sub-Saharan Africa | 113.84 (83.66 to 149.78) | 5.74 (4.20 to 7.67) | 204.65 (150.13 to 246.73) | 4.88 (3.55 to 5.91) | -0.51 (-0.68 to -0.34) | <0.001 |
| Tropical Latin America | 144.81 (133.19 to 154.24) | 2.24 (2.03 to 2.40) | 967.71 (852.14 to 1053.41) | 4.45 (3.91 to 4.85) | 2.26 (1.69 to 2.83) | <0.001 |
| Western Europe | 1719.55 (1578.25 to 1821.38) | 3.11 (2.84 to 3.29) | 7043.13 (5811.46 to 7901.73) | 6.74 (5.63 to 7.52) | 2.49 (2.12 to 2.86) | <0.001 |
| Western Sub-Saharan Africa | 759.99 (550.86 to 1088.97) | 11.51 (8.19 to 16.61) | 1589.91 (1022.22 to 1967.17) | 11.78 (7.61 to 14.57) | 0.10 (-0.02 to 0.23) | 0.107 |
|  | **DALYs (95% UI)** | | | | | |
| Andean Latin America | 5136.38 (2763.86 to 8261.13) | 322.28 (172.87 to 518.97) | 12757.46 (7036.99 to 21407.79) | 254.95 (140.57 to 427.73) | -0.76 (-0.87 to -0.66) | <0.001 |
| Australasia | 2999.48 (1529.86 to 5560.78) | 135.82 (69.16 to 251.47) | 6480.63 (3049.35 to 12771.73) | 122.51 (57.40 to 242.16) | -0.29 (-0.39 to -0.20) | <0.001 |
| Caribbean | 5147.82 (3594.48 to 7295.23) | 230.70 (160.67 to 326.99) | 9269.51 (6181.59 to 13354.19) | 194.42 (129.82 to 279.80) | -0.55 (-0.63 to -0.47) | <0.001 |
| Central Asia | 5626.01 (3350.06 to 8745.54) | 160.97 (95.81 to 250.13) | 11728.27 (7338.30 to 17672.97) | 192.37 (120.51 to 289.22) | 0.53 (0.34 to 0.71) | <0.001 |
| Central Europe | 19312.39 (13043.98 to 27890.10) | 145.69 (98.63 to 209.61) | 38912.78 (27728.75 to 55610.99) | 174.24 (124.16 to 249.01) | 0.60 (0.39 to 0.81) | <0.001 |
| Central Latin America | 20076.85 (13804.46 to 28489.27) | 316.83 (217.98 to 449.88) | 57490.44 (39369.96 to 83936.87) | 271.98 (186.17 to 396.80) | -0.48 (-0.64 to -0.33) | <0.001 |
| Central Sub-Saharan Africa | 6148.04 (3475.68 to 9716.68) | 421.19 (235.66 to 668.80) | 12059.38 (7167.17 to 18627.68) | 353.73 (207.47 to 546.90) | -0.58 (-0.65 to -0.52) | <0.001 |
| East Asia | 83612.34 (56155.17 to 120758.37) | 129.00 (86.31 to 186.50) | 199783.95 (123705.44 to 310992.54) | 101.35 (62.66 to 157.32) | -0.71 (-0.89 to -0.53) | <0.001 |
| Eastern Europe | 21872.18 (14169.73 to 34246.89) | 94.42 (61.31 to 147.32) | 30433.74 (18919.08 to 47221.00) | 92.11 (57.58 to 142.38) | -0.10 (-0.23 to 0.04) | 0.176 |
| Eastern Sub-Saharan Africa | 49927.82 (39123.08 to 61777.04) | 965.97 (752.85 to 1199.87) | 85775.23 (69083.92 to 105144.76) | 759.52 (609.85 to 932.33) | -0.77 (-0.83 to -0.71) | <0.001 |
| High-income Asia Pacific | 16456.85 (9396.26 to 27231.93) | 96.59 (55.19 to 159.72) | 63488.27 (38648.61 to 102776.51) | 127.80 (76.82 to 208.73) | 0.88 (0.60 to 1.16) | <0.001 |
| High-income North America | 34409.60 (21965.37 to 52894.43) | 100.32 (63.99 to 154.34) | 94798.00 (58030.14 to 148615.18) | 147.14 (90.16 to 230.58) | 1.28 (0.97 to 1.59) | <0.001 |
| North Africa and Middle East | 22134.63 (15584.96 to 31232.49) | 188.81 (132.70 to 266.26) | 54163.45 (37213.43 to 78371.60) | 166.47 (114.29 to 240.67) | -0.41 (-0.54 to -0.28) | <0.001 |
| Oceania | 258.32 (124.35 to 458.31) | 143.57 (72.35 to 247.11) | 630.09 (312.76 to 1071.06) | 136.98 (69.96 to 229.76) | -0.16 (-0.24 to -0.09) | <0.001 |
| South Asia | 125837.03 (84621.50 to 170194.27) | 321.88 (215.43 to 436.54) | 321170.57 (232580.44 to 410287.94) | 280.92 (202.35 to 359.24) | -0.41 (-0.67 to -0.15) | 0.002 |
| Southeast Asia | 25782.26 (17210.57 to 37556.87) | 144.58 (96.30 to 210.47) | 77680.53 (48027.65 to 117748.54) | 158.91 (98.29 to 241.13) | 0.31 (0.25 to 0.37) | <0.001 |
| Southern Latin America | 5331.72 (2947.23 to 8605.03) | 131.61 (72.62 to 212.05) | 11887.49 (6850.36 to 19012.42) | 146.13 (84.18 to 233.76) | 0.39 (0.12 to 0.65) | 0.005 |
| Southern Sub-Saharan Africa | 6099.76 (4088.95 to 8718.67) | 294.16 (196.15 to 420.66) | 12312.33 (8234.56 to 17633.68) | 282.60 (187.60 to 406.65) | -0.12 (-0.18 to -0.07) | <0.001 |
| Tropical Latin America | 15733.83 (9915.71 to 24710.93) | 224.14 (141.28 to 351.21) | 54076.87 (35666.78 to 80874.53) | 244.52 (161.20 to 365.56) | 0.28 (0.19 to 0.37) | <0.001 |
| Western Europe | 90547.45 (60144.00 to 138454.34) | 162.41 (107.90 to 248.19) | 223214.61 (152044.17 to 340728.98) | 233.90 (158.54 to 358.87) | 1.18 (1.02 to 1.34) | <0.001 |
| Western Sub-Saharan Africa | 27902.09 (19638.79 to 39361.74) | 410.06 (284.25 to 582.45) | 59237.97 (42343.36 to 77211.23) | 427.63 (304.09 to 562.52) | 0.17 (0.13 to 0.20) | <0.001 |
