# Supplementary material for: Global, regional, and national burdens of late-onset epilepsy in adults aged 65 years and older from 1990 to 2021: A population-based study
Source: PLoS One. 2025 Nov 19;20(11):e0336588. doi: 10.1371/journal.pone.0336588 (PMC12629476; doi:10.1371/journal.pone.0336588)
Supplement: S5 Table — Abbreviations: ASIR, age-standardized incidence rate; ASPR, age-standardized prevalence rate; ASMR, age-standardized mortality rate; AAPC, average annual percent changes; DALYs, disability-adjusted life years; CI, confidence interval; P, P value for the significant test of AAPC; LOE, late-onset epilepsy. Numbers in parentheses are 95% uncertainty intervals. (DOCX) [file pone.0336588.s005.docx]

**S5 Table.** ASIR, ASPR, ASMR, and age-standardized DALYs rate of LOE in individuals aged ≥65 years in 2021 and their AAPC between 1990-2021 in 204 countries and territories

**Abbreviations:** ASIR, age-standardized incidence rate; ASPR, age-standardized prevalence rate; ASMR, age-standardized mortality rate; AAPC, average annual percent changes; DALYs, disability-adjusted life years; CI, confidence interval; P, P value for the significant test of AAPC; LOE, late-onset epilepsy. Numbers in parentheses are 95% uncertainty intervals.

| **Countries and territories** | **Age-standardized rate in 2021 (per 100,000)** | | | | **AAPC 1990-2021 (%/year)** | | | |
| --- | --- | --- | --- | --- | --- | --- | --- | --- |
|  | **Incidence**  **(95% UI)** | **Prevalence**  **(95% UI)** | **Mortality**  **(95% UI)** | **DALYs**  **(95% UI)** | **Incidence** | **Prevalence** | **Mortality** | **DALYs** |
| Afghanistan | 19.34 (3.94 to 41.99) | 285.09 (65.51 to 554.99) | 7.52 (4.80 to 12.41) | 225.33 (129.14 to 357.11) | 0.03 (-0.05 to 0.10) | -0.08 (-0.18 to 0.02) | -0.16 (-0.22 to -0.09) | -0.24 (-0.26 to -0.22) |
| Albania | 22.63 (5.46 to 44.06) | 492.97 (129.27 to 853.93) | 3.65 (2.30 to 5.71) | 174.75 (78.33 to 325.96) | 0.71 (0.54 to 0.89) | 0.49 (0.29 to 0.69) | -1.21 (-1.51 to -0.91) | -0.62 (-0.81 to -0.43) |
| Algeria | 25.56 (6.39 to 49.99) | 338.05 (86.90 to 590.57) | 3.22 (2.01 to 5.35) | 132.70 (56.82 to 244.94) | -0.09 (-0.15 to -0.03) | -0.06 (-0.12 to 0.00) | -1.44 (-1.60 to -1.28) | -1.05 (-1.10 to -1.01) |
| American Samoa | 33.94 (8.55 to 62.86) | 521.82 (137.98 to 874.38) | 1.67 (1.04 to 2.50) | 177.91 (58.38 to 338.27) | 0.11 (-0.13 to 0.35) | -0.10 (-0.28 to 0.07) | 0.63 (0.15 to 1.12) | -0.25 (-0.45 to -0.05) |
| Andorra | 39.96 (10.78 to 73.66) | 528.35 (149.25 to 868.72) | 2.20 (1.36 to 3.29) | 136.87 (53.99 to 279.77) | 0.25 (0.20 to 0.31) | 0.05 (0.00 to 0.10) | -1.23 (-1.60 to -0.86) | -0.55 (-0.82 to -0.27) |
| Angola | 62.46 (14.04 to 126.54) | 933.52 (231.18 to 1664.13) | 8.12 (5.15 to 12.02) | 448.41 (195.99 to 802.05) | 0.55 (0.48 to 0.63) | 0.54 (0.49 to 0.59) | -0.91 (-1.02 to -0.8) | -0.23 (-0.29 to -0.18) |
| Antigua and Barbuda | 59.08 (15.66 to 115.44) | 979.96 (284.05 to 1628.88) | 10.81 (9.02 to 12.90) | 423.44 (229.09 to 697.22) | 0.65 (0.58 to 0.71) | 0.34 (0.24 to 0.44) | -0.55 (-2.38 to 1.32) | -0.37 (-1.22 to 0.49) |
| Argentina | 20.55 (4.97 to 38.64) | 310.44 (81.99 to 525.61) | 1.99 (1.68 to 2.33) | 109.50 (48.35 to 201.12) | 0.52 (0.49 to 0.55) | 0.42 (0.39 to 0.45) | 0.95 (0.28 to 1.62) | 0.16 (-0.09 to 0.40) |
| Armenia | 17.00 (4.94 to 34.11) | 359.15 (112.90 to 643.37) | 1.48 (1.18 to 1.77) | 113.00 (45.35 to 218.82) | 0.33 (0.31 to 0.36) | 0.18 (0.03 to 0.33) | 0.47 (-1.08 to 2.04) | -0.28 (-0.43 to -0.13) |
| Australia | 31.61 (8.66 to 60.87) | 455.50 (132.76 to 782.24) | 2.41 (1.98 to 2.80) | 124.62 (53.17 to 254.88) | 0.33 (0.24 to 0.42) | 0.23 (0.20 to 0.26) | -0.35 (-1.21 to 0.51) | -0.22 (-0.34 to -0.11) |
| Austria | 37.76 (10.24 to 68.53) | 512.32 (151.05 to 802.99) | 4.93 (3.98 to 5.87) | 175.28 (91.94 to 319.43) | 0.92 (0.88 to 0.96) | 0.72 (0.69 to 0.76) | 2.58 (1.69 to 3.49) | 1.08 (0.76 to 1.41) |
| Azerbaijan | 25.20 (6.48 to 50.16) | 550.71 (159.57 to 953.29) | 2.01 (1.39 to 2.84) | 184.48 (69.49 to 356.67) | 1.31 (1.12 to 1.50) | 1.11 (1.03 to 1.18) | -0.43 (-0.72 to -0.14) | 0.38 (0.22 to 0.54) |
| Bahamas | 38.40 (10.81 to 74.65) | 636.48 (197.82 to 1062.58) | 3.41 (2.66 to 4.29) | 232.60 (98.47 to 424.28) | -0.07 (-0.17 to 0.03) | -0.47 (-0.51 to -0.42) | -0.65 (-1.87 to 0.58) | -0.78 (-1.15 to -0.41) |
| Bahrain | 58.50 (15.41 to 111.03) | 716.36 (197.51 to 1227.87) | 6.84 (4.19 to 9.82) | 264.61 (127.46 to 467.45) | 0.39 (0.35 to 0.44) | -0.03 (-0.08 to 0.02) | -1.42 (-1.89 to -0.94) | -1.17 (-1.34 to -0.99) |
| Bangladesh | 22.36 (5.80 to 44.61) | 288.66 (80.18 to 534.46) | 4.48 (2.25 to 7.23) | 149.51 (72.82 to 257.39) | 0.20 (0.17 to 0.23) | 0.13 (0.09 to 0.18) | -1.20 (-1.56 to -0.84) | -1.00 (-1.20 to -0.80) |
| Barbados | 41.31 (11.37 to 79.47) | 637.56 (195.84 to 1071.69) | 5.09 (3.87 to 6.40) | 242.03 (122.70 to 427.43) | -0.10 (-0.18 to -0.01) | -0.32 (-0.35 to -0.29) | -0.50 (-1.37 to 0.37) | -0.70 (-1.03 to -0.36) |
| Belarus | 14.49 (3.58 to 29.09) | 300.99 (82.68 to 512.33) | 1.94 (1.49 to 2.44) | 97.48 (42.78 to 193.84) | 0.69 (0.61 to 0.77) | 0.06 (-0.02 to 0.14) | 0.35 (-0.01 to 0.71) | -0.24 (-0.46 to -0.02) |
| Belgium | 61.79 (16.71 to 117.23) | 828.19 (240.34 to 1406.25) | 8.76 (7.05 to 10.34) | 297.01 (165.47 to 531.38) | 1.12 (1.06 to 1.18) | 1.08 (1.03 to 1.13) | 2.33 (1.51 to 3.15) | 1.17 (0.92 to 1.42) |
| Belize | 30.68 (7.68 to 61.08) | 490.10 (135.06 to 854.49) | 3.76 (3.10 to 4.43) | 202.52 (91.98 to 363.81) | 0.33 (0.25 to 0.41) | 0.40 (0.32 to 0.48) | -0.83 (-2.36 to 0.72) | -0.25 (-0.88 to 0.38) |
| Benin | 48.46 (11.07 to 100.79) | 624.19 (148.72 to 1193.87) | 12.82 (7.80 to 19.94) | 452.32 (238.18 to 744.05) | 0.49 (0.30 to 0.68) | 0.38 (0.16 to 0.60) | 0.09 (-0.03 to 0.22) | 0.08 (-0.05 to 0.21) |
| Bermuda | 44.98 (12.46 to 84.14) | 677.25 (203.54 to 1127.11) | 2.98 (2.38 to 3.78) | 188.60 (78.50 to 372.31) | -0.26 (-0.29 to -0.24) | -0.66 (-0.74 to -0.59) | -1.82 (-2.66 to -0.98) | -1.61 (-1.73 to -1.48) |
| Bhutan | 36.55 (9.05 to 74.82) | 494.43 (131.96 to 915.95) | 10.23 (6.26 to 15.45) | 299.44 (163.46 to 499.34) | 0.90 (0.84 to 0.96) | 0.71 (0.65 to 0.76) | -0.92 (-0.99 to -0.86) | -0.61 (-0.68 to -0.53) |
| Bolivia (Plurinational State of) | 35.21 (8.22 to 71.10) | 646.89 (162.28 to 1122.54) | 4.21 (2.67 to 6.34) | 267.92 (105.62 to 486.19) | 0.64 (0.54 to 0.73) | 0.25 (0.17 to 0.32) | -1.35 (-1.43 to -1.27) | -0.69 (-0.75 to -0.64) |
| Bosnia and Herzegovina | 18.39 (4.59 to 36.41) | 449.19 (124.44 to 753.54) | 4.08 (2.17 to 6.09) | 171.24 (82.39 to 307.90) | 1.14 (1.11 to 1.18) | 1.25 (1.18 to 1.31) | 0.31 (0.08 to 0.55) | 0.41 (0.35 to 0.47) |
| Botswana | 56.02 (14.41 to 109.57) | 832.20 (231.37 to 1426.72) | 5.81 (3.82 to 8.40) | 352.83 (148.79 to 627.17) | 1.22 (1.11 to 1.34) | 1.17 (1.01 to 1.33) | -0.52 (-0.99 to -0.04) | 0.33 (0.24 to 0.42) |
| Brazil | 41.80 (20.54 to 69.67) | 659.33 (399.40 to 969.51) | 4.47 (3.92 to 4.87) | 245.17 (161.64 to 366.68) | 0.68 (0.65 to 0.70) | 0.29 (0.27 to 0.32) | 2.32 (1.72 to 2.92) | 0.28 (0.19 to 0.37) |
| Brunei Darussalam | 49.98 (14.76 to 95.37) | 727.05 (229.03 to 1197.46) | 3.45 (2.45 to 4.70) | 234.82 (95.00 to 437.92) | 0.07 (-0.06 to 0.19) | -0.47 (-0.52 to -0.41) | -1.34 (-1.72 to -0.96) | -0.99 (-1.07 to -0.91) |
| Bulgaria | 16.45 (3.93 to 32.38) | 451.10 (124.53 to 749.78) | 3.82 (2.96 to 4.80) | 171.70 (85.82 to 304.57) | 0.40 (0.32 to 0.48) | 0.42 (0.34 to 0.50) | 1.51 (0.46 to 2.57) | 0.60 (0.27 to 0.93) |
| Burkina Faso | 48.01 (8.01 to 107.10) | 609.01 (112.26 to 1244.29) | 15.87 (9.40 to 25.37) | 513.12 (262.84 to 830.80) | 0.31 (0.24 to 0.39) | 0.33 (0.19 to 0.46) | 0.39 (0.19 to 0.6) | 0.27 (0.12 to 0.41) |
| Burundi | 35.92 (5.95 to 77.05) | 430.33 (75.33 to 871.87) | 46.18 (29.08 to 70.21) | 892.61 (570.25 to 1336.27) | -0.27 (-0.39 to -0.15) | -0.67 (-0.74 to -0.61) | 0.31 (0.24 to 0.38) | 0.01 (-0.05 to 0.08) |
| Cabo Verde | 64.31 (14.90 to 128.70) | 876.22 (213.60 to 1589.59) | 9.83 (6.12 to 14.75) | 423.30 (207.26 to 725.44) | 1.48 (1.44 to 1.52) | 1.55 (1.38 to 1.71) | -1.12 (-1.6 to -0.64) | -0.23 (-0.49 to 0.03) |
| Cambodia | 24.32 (6.85 to 50.27) | 355.17 (110.28 to 661.13) | 2.09 (1.21 to 3.96) | 146.49 (59.33 to 265.81) | 0.75 (0.59 to 0.91) | 0.71 (0.49 to 0.93) | -1.01 (-1.05 to -0.96) | -0.16 (-0.26 to -0.05) |
| Cameroon | 50.92 (11.73 to 104.75) | 650.04 (167.99 to 1217.59) | 12.01 (6.83 to 19.10) | 443.00 (235.51 to 704.63) | 0.19 (0.04 to 0.34) | 0.12 (-0.10 to 0.34) | -0.14 (-0.24 to -0.03) | -0.12 (-0.25 to 0.01) |
| Canada | 34.21 (9.23 to 63.93) | 446.92 (124.47 to 733.97) | 2.41 (2.02 to 2.79) | 123.23 (54.64 to 243.48) | 0.15 (-0.12 to 0.42) | 0.21 (0.04 to 0.37) | -0.62 (-1.50 to 0.27) | -0.35 (-0.84 to 0.15) |
| Central African Republic | 33.97 (6.70 to 71.46) | 475.31 (97.89 to 901.48) | 10.38 (6.56 to 15.65) | 360.57 (191.44 to 582.51) | -0.26 (-0.29 to -0.24) | -0.47 (-0.52 to -0.42) | -0.23 (-0.28 to -0.17) | -0.40 (-0.43 to -0.36) |
| Chad | 40.75 (6.81 to 89.29) | 529.89 (90.12 to 1069.73) | 17.16 (10.08 to 28.20) | 531.99 (283.45 to 856.30) | 0.55 (0.52 to 0.58) | 0.65 (0.63 to 0.67) | 1.06 (0.87 to 1.26) | 0.91 (0.80 to 1.02) |
| Chile | 40.86 (9.73 to 80.42) | 656.98 (188.30 to 1145.58) | 4.26 (3.55 to 4.96) | 215.53 (99.46 to 410.67) | 1.09 (1.04 to 1.13) | 0.84 (0.80 to 0.87) | 0.53 (-0.67 to 1.75) | 0.15 (-0.17 to 0.48) |
| China | 22.20 (11.49 to 36.18) | 332.15 (218.89 to 474.95) | 1.31 (1.01 to 1.67) | 99.82 (61.01 to 156.55) | 0.80 (0.56 to 1.03) | 0.70 (0.40 to 0.99) | -1.94 (-2.19 to -1.68) | -0.72 (-0.95 to -0.50) |
| Colombia | 38.61 (8.86 to 74.93) | 633.59 (166.37 to 1122.44) | 3.42 (2.64 to 4.18) | 213.00 (91.35 to 415.90) | 0.36 (0.32 to 0.39) | 0.16 (0.12 to 0.19) | -0.23 (-1.23 to 0.78) | -0.50 (-0.63 to -0.36) |
| Comoros | 49.70 (13.40 to 97.56) | 605.08 (166.57 to 1085.76) | 34.76 (21.08 to 53.99) | 749.91 (450.77 to 1129.52) | 0.37 (0.31 to 0.42) | 0.05 (-0.04 to 0.14) | -0.40 (-0.55 to -0.25) | -0.45 (-0.53 to -0.37) |
| Congo | 62.84 (15.93 to 128.54) | 936.69 (253.02 to 1700.47) | 7.55 (4.94 to 10.96) | 429.89 (189.05 to 753.55) | 0.58 (0.53 to 0.63) | 0.35 (0.30 to 0.40) | -0.80 (-0.92 to -0.67) | -0.33 (-0.36 to -0.29) |
| Cook Islands | 36.03 (10.27 to 67.22) | 564.40 (182.44 to 931.87) | 0.72 (0.43 to 1.12) | 148.54 (48.54 to 295.76) | 0.23 (0.10 to 0.37) | 0.09 (-0.02 to 0.20) | -1.92 (-2.07 to -1.78) | -0.68 (-0.73 to -0.62) |
| Costa Rica | 41.94 (10.96 to 84.66) | 686.27 (193.88 to 1177.26) | 4.57 (3.73 to 5.45) | 236.07 (111.95 to 428.85) | 0.16 (0.09 to 0.22) | 0.04 (-0.04 to 0.11) | 0.01 (-0.46 to 0.47) | -0.33 (-0.4 to -0.26) |
| Coted'Ivoire | 54.35 (12.06 to 111.95) | 729.14 (175.28 to 1348.48) | 12.73 (7.63 to 19.58) | 483.99 (255.97 to 789.02) | 0.39 (0.29 to 0.49) | 0.35 (0.27 to 0.44) | 0.06 (-0.11 to 0.23) | 0.08 (0.04 to 0.13) |
| Croatia | 21.51 (5.87 to 41.77) | 572.25 (161.32 to 940.34) | 4.10 (3.17 to 5.04) | 188.04 (89.80 to 357.31) | -0.64 (-0.77 to -0.51) | -0.74 (-0.82 to -0.66) | 0.83 (-0.49 to 2.16) | -0.55 (-0.86 to -0.24) |
| Cuba | 19.35 (4.93 to 37.25) | 292.94 (82.85 to 502.62) | 2.39 (1.96 to 2.87) | 108.27 (51.66 to 197.97) | 0.02 (-0.05 to 0.08) | -0.07 (-0.12 to -0.01) | -0.05 (-0.76 to 0.66) | -0.33 (-0.84 to 0.18) |
| Cyprus | 37.69 (9.24 to 73.60) | 452.76 (118.34 to 779.02) | 3.54 (2.01 to 5.08) | 139.48 (63.22 to 266.44) | 0.13 (0.07 to 0.20) | -0.26 (-0.33 to -0.19) | -2.41 (-3.00 to -1.82) | -1.53 (-1.81 to -1.26) |
| Czechia | 22.41 (5.90 to 42.33) | 588.16 (167.73 to 934.70) | 4.95 (3.84 to 6.09) | 206.70 (107.80 to 368.67) | 1.05 (0.97 to 1.12) | 0.81 (0.75 to 0.86) | 2.43 (1.55 to 3.32) | 0.97 (0.75 to 1.19) |
| Democratic People's Republic of Korea | 12.97 (3.27 to 26.05) | 207.07 (55.72 to 383.22) | 1.36 (0.82 to 2.41) | 83.31 (33.79 to 165.82) | -0.93 (-1.04 to -0.81) | -1.22 (-1.41 to -1.04) | -0.93 (-0.99 to -0.87) | -1.42 (-1.53 to -1.31) |
| Democratic Republic of the Congo | 38.01 (7.82 to 78.76) | 517.68 (109.21 to 962.94) | 7.99 (4.55 to 13.02) | 311.97 (148.04 to 517.73) | -0.42 (-0.50 to -0.35) | -0.60 (-0.75 to -0.45) | -0.80 (-0.86 to -0.74) | -0.82 (-0.94 to -0.71) |
| Denmark | 28.55 (7.74 to 53.04) | 435.53 (124.66 to 703.64) | 6.71 (5.46 to 7.98) | 189.84 (117.68 to 321.40) | 0.61 (0.49 to 0.73) | 0.63 (0.56 to 0.69) | 2.93 (1.97 to 3.9) | 1.31 (0.87 to 1.75) |
| Djibouti | 56.02 (13.49 to 110.13) | 689.08 (184.08 to 1234.31) | 33.88 (20.99 to 50.40) | 768.03 (484.79 to 1125.04) | 0.44 (0.32 to 0.57) | 0.23 (0.18 to 0.27) | -0.20 (-0.28 to -0.11) | -0.18 (-0.23 to -0.12) |
| Dominica | 49.71 (14.24 to 96.95) | 796.87 (239.70 to 1310.41) | 9.05 (6.08 to 13.02) | 385.24 (202.55 to 626.08) | 0.53 (0.48 to 0.57) | 0.25 (0.23 to 0.28) | -0.87 (-1.11 to -0.63) | -0.41 (-0.58 to -0.25) |
| Dominican Republic | 39.20 (10.35 to 80.15) | 597.64 (176.62 to 1040.19) | 2.90 (1.81 to 4.49) | 218.69 (82.88 to 408.67) | 1.03 (0.97 to 1.10) | 1.30 (1.21 to 1.4) | -1.13 (-1.45 to -0.81) | 0.28 (0.18 to 0.38) |
| Ecuador | 65.61 (17.63 to 123.11) | 1019.76 (302.71 to 1740.27) | 4.93 (3.47 to 6.62) | 353.04 (150.62 to 655.72) | 0.47 (0.06 to 0.88) | 0.27 (0.03 to 0.52) | -0.56 (-2.88 to 1.81) | -0.55 (-1.06 to -0.05) |
| Egypt | 29.10 (7.46 to 57.60) | 402.63 (110.00 to 703.30) | 2.62 (1.80 to 3.68) | 150.36 (64.37 to 288.73) | 0.89 (0.75 to 1.03) | 0.84 (0.66 to 1.02) | 0.35 (-0.02 to 0.72) | 0.16 (-0.19 to 0.52) |
| El Salvador | 33.02 (7.47 to 63.50) | 528.74 (131.23 to 939.29) | 2.08 (1.42 to 2.90) | 170.37 (63.72 to 332.00) | 0.72 (0.64 to 0.81) | 0.71 (0.59 to 0.82) | -0.94 (-1.56 to -0.31) | -0.33 (-0.46 to -0.19) |
| Equatorial Guinea | 87.03 (19.32 to 168.26) | 1364.46 (320.42 to 2370.71) | 5.77 (3.66 to 8.57) | 491.89 (180.07 to 921.07) | 2.47 (2.31 to 2.64) | 2.62 (2.51 to 2.74) | -1.89 (-2.03 to -1.75) | 0.60 (0.48 to 0.73) |
| Eritrea | 41.98 (9.51 to 89.20) | 529.41 (120.53 to 1016.10) | 40.59 (26.58 to 59.35) | 863.50 (561.14 to 1246.74) | 0.09 (-0.08 to 0.25) | 0.13 (0.02 to 0.23) | 1.35 (1.28 to 1.42) | 0.79 (0.74 to 0.84) |
| Estonia | 20.22 (5.27 to 39.70) | 557.95 (166.77 to 909.00) | 3.18 (2.50 to 3.86) | 171.69 (75.89 to 324.24) | 0.75 (0.71 to 0.79) | 1.14 (0.95 to 1.34) | -0.21 (-1.14 to 0.74) | 0.33 (-0.05 to 0.71) |
| Eswatini | 49.99 (10.93 to 102.34) | 708.69 (175.01 to 1256.64) | 6.05 (3.78 to 9.34) | 334.59 (151.74 to 580.29) | 0.86 (0.82 to 0.91) | 0.71 (0.60 to 0.82) | -0.64 (-0.71 to -0.56) | 0.09 (0.03 to 0.15) |
| Ethiopia | 39.41 (17.37 to 70.97) | 431.17 (226.77 to 696.10) | 40.63 (29.55 to 53.00) | 782.82 (588.14 to 1010.59) | 0.71 (0.70 to 0.72) | 0.52 (0.49 to 0.54) | -1.43 (-1.54 to -1.32) | -1.36 (-1.44 to -1.28) |
| Fiji | 31.38 (8.61 to 60.52) | 486.66 (136.96 to 834.20) | 2.11 (1.26 to 3.28) | 182.29 (65.26 to 334.01) | 0.40 (0.30 to 0.50) | 0.18 (0.04 to 0.32) | -0.46 (-0.59 to -0.33) | -0.13 (-0.28 to 0.02) |
| Finland | 30.04 (8.29 to 56.56) | 482.32 (144.27 to 803.18) | 5.40 (4.17 to 6.53) | 178.30 (100.35 to 319.66) | 0.77 (0.67 to 0.87) | 0.73 (0.67 to 0.79) | 2.02 (1.55 to 2.49) | 0.88 (0.75 to 1.00) |
| France | 66.00 (22.29 to 123.77) | 798.75 (285.84 to 1341.87) | 7.89 (6.30 to 9.42) | 269.72 (144.44 to 510.96) | 0.93 (0.82 to 1.03) | 0.91 (0.85 to 0.97) | 1.21 (0.90 to 1.53) | 0.63 (0.42 to 0.84) |
| Gabon | 79.41 (17.88 to 160.71) | 1261.56 (273.19 to 2254.83) | 6.73 (4.48 to 9.88) | 487.44 (186.30 to 878.65) | 0.63 (0.57 to 0.69) | 0.36 (0.34 to 0.38) | -1.54 (-1.64 to -1.44) | -0.54 (-0.58 to -0.5) |
| Gambia | 44.28 (10.31 to 88.81) | 543.38 (126.88 to 994.29) | 13.64 (8.35 to 20.50) | 436.52 (248.28 to 672.24) | 0.36 (0.29 to 0.43) | 0.37 (0.31 to 0.43) | 0.32 (-0.28 to 0.92) | 0.20 (-0.20 to 0.59) |
| Georgia | 21.34 (5.39 to 41.48) | 490.82 (145.77 to 840.20) | 2.40 (1.86 to 2.93) | 170.65 (71.42 to 316.50) | -0.12 (-0.24 to 0.01) | -0.21 (-0.33 to -0.08) | 1.59 (-0.42 to 3.63) | -0.10 (-0.47 to 0.27) |
| Germany | 97.31 (30.35 to 153.28) | 1149.36 (371.91 to 1785.57) | 10.38 (8.35 to 12.12) | 379.69 (208.69 to 701.32) | 2.68 (2.54 to 2.81) | 2.06 (1.97 to 2.14) | 4.63 (3.81 to 5.47) | 2.41 (2.09 to 2.74) |
| Ghana | 46.11 (10.89 to 89.35) | 621.68 (157.85 to 1080.45) | 7.29 (4.31 to 11.13) | 332.17 (155.31 to 564.59) | 0.87 (0.8 to 0.94) | 0.94 (0.85 to 1.04) | 0.37 (0.27 to 0.47) | 0.50 (0.4 to 0.6) |
| Greece | 27.04 (6.85 to 50.42) | 368.46 (92.17 to 602.80) | 4.28 (3.55 to 4.97) | 140.92 (79.82 to 243.58) | 0.12 (-0.03 to 0.28) | 0.03 (-0.07 to 0.14) | 3.13 (2.37 to 3.9) | 1.00 (0.62 to 1.39) |
| Greenland | 63.77 (16.65 to 129.35) | 1030.03 (314.50 to 1764.38) | 7.22 (2.78 to 11.78) | 386.41 (148.67 to 703.81) | 0.46 (0.39 to 0.54) | 0.17 (0.05 to 0.29) | -0.74 (-1.1 to -0.38) | -0.51 (-0.76 to -0.27) |
| Grenada | 46.38 (11.00 to 91.16) | 678.76 (173.32 to 1184.51) | 5.87 (4.74 to 7.13) | 286.46 (142.68 to 491.32) | 1.12 (1.03 to 1.22) | 0.67 (0.62 to 0.73) | -0.62 (-2.21 to 1.00) | -0.07 (-0.58 to 0.44) |
| Guam | 35.34 (10.76 to 66.62) | 551.90 (180.02 to 927.96) | 0.21 (0.12 to 0.32) | 152.90 (43.83 to 319.95) | 0.03 (-0.05 to 0.10) | -0.13 (-0.24 to -0.01) | -1.40 (-3.45 to 0.70) | -0.38 (-0.48 to -0.28) |
| Guatemala | 37.16 (9.05 to 73.38) | 590.41 (159.47 to 1022.98) | 4.21 (3.38 to 5.14) | 241.22 (106.96 to 421.53) | 0.75 (0.66 to 0.84) | 0.70 (0.66 to 0.75) | -1.94 (-3.12 to -0.74) | -0.70 (-1.14 to -0.25) |
| Guinea | 44.31 (9.16 to 96.97) | 571.68 (126.58 to 1139.90) | 15.25 (9.26 to 23.83) | 496.84 (280.90 to 799.93) | 0.46 (0.37 to 0.55) | 0.37 (0.3 to 0.45) | 0.73 (0.60 to 0.86) | 0.51 (0.44 to 0.59) |
| Guinea-Bissau | 44.43 (8.07 to 93.92) | 592.51 (106.65 to 1110.09) | 15.73 (10.00 to 23.36) | 523.39 (287.09 to 825.83) | 0.22 (0.09 to 0.35) | 0.07 (-0.04 to 0.18) | 0.01 (-0.07 to 0.10) | -0.09 (-0.14 to -0.03) |
| Guyana | 36.69 (9.42 to 73.80) | 618.38 (166.86 to 1080.21) | 7.06 (5.21 to 9.22) | 315.26 (162.95 to 513.27) | 0.48 (0.45 to 0.51) | 0.49 (0.45 to 0.53) | -0.68 (-1.67 to 0.31) | -0.26 (-0.75 to 0.23) |
| Haiti | 25.54 (5.17 to 53.04) | 378.36 (88.20 to 700.38) | 10.00 (6.46 to 14.88) | 308.01 (176.71 to 468.06) | 0.02 (-0.07 to 0.10) | -0.32 (-0.44 to -0.19) | -0.93 (-1.03 to -0.83) | -0.8 (-0.88 to -0.71) |
| Honduras | 46.57 (9.18 to 94.50) | 715.54 (147.89 to 1309.30) | 7.27 (4.67 to 10.68) | 342.07 (157.78 to 593.19) | 0.68 (0.6 to 0.76) | 0.8 (0.74 to 0.86) | -0.17 (-0.38 to 0.04) | 0.15 (0.05 to 0.24) |
| Hungary | 16.31 (4.52 to 30.60) | 426.50 (132.98 to 703.40) | 2.88 (2.31 to 3.52) | 143.22 (67.22 to 268.55) | 0.24 (0.18 to 0.30) | 0.00 (-0.02 to 0.03) | 1.37 (0.35 to 2.40) | -0.03 (-0.28 to 0.21) |
| Iceland | 40.78 (10.88 to 75.67) | 525.42 (146.83 to 861.87) | 4.16 (3.33 to 4.97) | 163.31 (80.59 to 316.80) | 0.85 (0.81 to 0.89) | 0.50 (0.46 to 0.53) | 1.62 (1.06 to 2.17) | 0.62 (0.45 to 0.80) |
| India | 39.09 (21.49 to 61.08) | 451.61 (305.34 to 640.74) | 9.52 (6.33 to 11.44) | 278.10 (201.67 to 357.08) | 0.43 (0.34 to 0.51) | 0.38 (0.23 to 0.52) | -0.23 (-0.91 to 0.45) | -0.35 (-0.67 to -0.04) |
| Indonesia | 24.09 (12.20 to 40.25) | 340.39 (209.61 to 498.96) | 1.40 (0.37 to 2.34) | 127.91 (74.88 to 195.69) | 1.09 (1.01 to 1.17) | 0.94 (0.86 to 1.02) | -0.03 (-0.16 to 0.09) | 0.40 (0.25 to 0.55) |
| Iran (Islamic Republic of) | 30.15 (15.63 to 49.12) | 390.87 (249.26 to 558.13) | 1.92 (1.43 to 3.29) | 122.72 (74.61 to 190.27) | 0.62 (0.60 to 0.65) | 0.52 (0.49 to 0.55) | -1.84 (-2.04 to -1.64) | -0.71 (-0.79 to -0.64) |
| Iraq | 23.90 (6.68 to 45.59) | 330.45 (96.20 to 552.16) | 2.23 (1.38 to 3.72) | 119.21 (52.80 to 209.28) | 0.85 (0.64 to 1.07) | 0.66 (0.54 to 0.78) | -1.18 (-1.30 to -1.05) | -0.48 (-0.53 to -0.43) |
| Ireland | 43.29 (11.73 to 78.86) | 582.21 (174.32 to 965.97) | 5.55 (4.42 to 6.67) | 193.97 (102.77 to 352.73) | 0.96 (0.85 to 1.07) | 0.86 (0.76 to 0.97) | 0.66 (-0.14 to 1.46) | 0.45 (0.28 to 0.61) |
| Israel | 39.20 (11.41 to 72.53) | 506.40 (164.15 to 825.60) | 4.92 (3.89 to 5.88) | 179.84 (94.62 to 331.35) | 0.88 (0.84 to 0.92) | 0.77 (0.74 to 0.79) | 1.76 (1.35 to 2.17) | 0.75 (0.37 to 1.13) |
| Italy | 37.80 (18.83 to 62.51) | 461.80 (284.55 to 672.43) | 6.58 (5.19 to 7.55) | 185.67 (131.51 to 272.21) | 0.87 (0.56 to 1.17) | 0.41 (0.31 to 0.51) | 4.33 (3.60 to 5.05) | 1.51 (1.10 to 1.92) |
| Jamaica | 37.75 (9.99 to 72.07) | 577.34 (157.94 to 980.56) | 6.81 (5.02 to 8.90) | 270.29 (141.55 to 453.73) | 0.07 (-0.03 to 0.18) | -0.08 (-0.15 to -0.01) | 0.56 (-0.57 to 1.70) | 0.00 (-0.49 to 0.48) |
| Japan | 31.89 (16.07 to 52.37) | 416.15 (259.06 to 602.60) | 2.46 (2.00 to 2.75) | 119.06 (74.57 to 191.65) | 0.83 (0.73 to 0.93) | 0.58 (0.38 to 0.77) | 5.34 (4.83 to 5.85) | 1.05 (0.87 to 1.24) |
| Jordan | 21.48 (5.83 to 40.57) | 290.40 (82.57 to 500.73) | 2.07 (1.37 to 3.00) | 100.31 (46.67 to 183.56) | 0.27 (0.18 to 0.36) | 0.12 (0.07 to 0.17) | -2.19 (-2.80 to -1.58) | -1.29 (-1.55 to -1.03) |
| Kazakhstan | 23.91 (5.46 to 47.81) | 627.21 (172.12 to 1054.55) | 2.82 (2.36 to 3.39) | 207.33 (81.29 to 401.06) | 0.89 (0.84 to 0.94) | 1.02 (0.99 to 1.04) | 2.57 (1.50 to 3.64) | 0.83 (0.7 to 0.97) |
| Kenya | 60.64 (32.59 to 98.02) | 678.38 (462.31 to 960.06) | 29.75 (21.72 to 38.44) | 704.26 (538.58 to 885.86) | 0.65 (0.59 to 0.71) | 0.41 (0.33 to 0.49) | -0.64 (-0.72 to -0.56) | -0.30 (-0.34 to -0.27) |
| Kiribati | 21.95 (5.07 to 45.07) | 349.96 (90.94 to 626.06) | 2.73 (1.58 to 4.73) | 161.65 (61.61 to 292.80) | 0.14 (0.10 to 0.17) | -0.20 (-0.24 to -0.16) | -0.12 (-0.19 to -0.06) | -0.31 (-0.34 to -0.28) |
| Kuwait | 37.48 (9.58 to 68.86) | 492.04 (132.00 to 817.03) | 2.02 (1.48 to 2.74) | 137.29 (50.64 to 272.55) | 0.30 (0.25 to 0.35) | 0.06 (-0.02 to 0.15) | -0.79 (-2.71 to 1.16) | -0.77 (-1.17 to -0.37) |
| Kyrgyzstan | 16.49 (3.90 to 31.76) | 420.10 (114.15 to 689.85) | 3.22 (2.43 to 4.04) | 170.44 (78.41 to 298.26) | 0.28 (0.17 to 0.39) | 0.11 (-0.01 to 0.24) | 0.43 (-0.86 to 1.74) | -0.31 (-0.81 to 0.19) |
| Lao People's Democratic Republic | 26.39 (6.01 to 53.76) | 382.84 (93.62 to 684.30) | 2.02 (1.12 to 4.19) | 159.00 (57.03 to 292.21) | 1.05 (0.92 to 1.17) | 0.94 (0.82 to 1.06) | -1.29 (-1.31 to -1.26) | -0.09 (-0.26 to 0.08) |
| Latvia | 15.44 (3.99 to 29.10) | 419.19 (126.43 to 693.08) | 2.27 (1.77 to 2.82) | 135.44 (58.72 to 267.43) | 0.71 (0.66 to 0.77) | 0.90 (0.85 to 0.95) | 0.24 (-0.74 to 1.23) | 0.51 (0.34 to 0.67) |
| Lebanon | 24.44 (6.82 to 47.95) | 336.37 (99.45 to 595.03) | 6.95 (4.70 to 10.47) | 178.23 (102.60 to 285.73) | 0.02 (-0.02 to 0.07) | 0.02 (-0.03 to 0.08) | -0.27 (-0.40 to -0.14) | -0.61 (-0.71 to -0.52) |
| Lesotho | 39.02 (7.97 to 78.78) | 550.38 (118.41 to 977.25) | 6.97 (4.41 to 10.54) | 310.63 (143.84 to 504.42) | 1.21 (1.14 to 1.28) | 1.28 (1.22 to 1.34) | 0.53 (0.21 to 0.85) | 0.94 (0.81 to 1.07) |
| Liberia | 44.40 (8.97 to 92.44) | 569.99 (118.82 to 1067.23) | 12.08 (6.99 to 19.98) | 412.19 (214.66 to 673.52) | -0.07 (-0.27 to 0.13) | -0.25 (-0.45 to -0.04) | -0.20 (-0.40 to 0.00) | -0.40 (-0.60 to -0.21) |
| Libya | 24.03 (6.55 to 45.18) | 322.03 (96.76 to 548.81) | 2.68 (1.54 to 4.24) | 123.56 (57.09 to 215.12) | 0.01 (-0.04 to 0.06) | -0.22 (-0.28 to -0.16) | -1.60 (-1.87 to -1.32) | -1.09 (-1.22 to -0.95) |
| Lithuania | 16.83 (4.85 to 32.43) | 472.93 (151.43 to 771.62) | 2.99 (2.41 to 3.61) | 159.36 (75.87 to 297.82) | 0.85 (0.77 to 0.92) | 0.84 (0.72 to 0.96) | 0.27 (-0.52 to 1.07) | 0.48 (0.13 to 0.83) |
| Luxembourg | 63.48 (17.97 to 118.25) | 831.82 (264.98 to 1367.43) | 8.24 (6.56 to 9.90) | 290.22 (155.35 to 509.93) | 0.80 (0.77 to 0.84) | 0.63 (0.59 to 0.67) | 1.61 (0.91 to 2.31) | 0.63 (0.33 to 0.94) |
| Madagascar | 40.73 (9.59 to 83.65) | 494.63 (124.60 to 953.38) | 25.91 (16.52 to 38.62) | 591.57 (371.23 to 867.33) | 0.13 (0.10 to 0.16) | -0.02 (-0.06 to 0.01) | -1.01 (-1.15 to -0.86) | -0.84 (-0.95 to -0.74) |
| Malawi | 41.30 (9.00 to 85.08) | 479.94 (109.91 to 909.58) | 37.71 (25.45 to 55.20) | 794.60 (544.01 to 1139.52) | 0.26 (0.12 to 0.41) | 0.06 (-0.05 to 0.18) | -1.10 (-1.18 to -1.02) | -0.79 (-0.87 to -0.72) |
| Malaysia | 41.75 (10.71 to 82.22) | 625.75 (169.99 to 1081.95) | 3.33 (2.33 to 4.69) | 222.25 (88.84 to 411.21) | 0.72 (0.68 to 0.75) | 0.67 (0.62 to 0.72) | -0.73 (-1.00 to -0.46) | -0.21 (-0.35 to -0.07) |
| Maldives | 52.76 (13.95 to 104.04) | 675.52 (202.90 to 1217.54) | 3.91 (2.32 to 5.99) | 232.87 (95.60 to 455.74) | 1.19 (1.08 to 1.31) | 0.56 (0.47 to 0.65) | -2.69 (-3.52 to -1.86) | -1.13 (-1.36 to -0.91) |
| Mali | 36.73 (7.67 to 79.79) | 443.89 (99.88 to 892.95) | 15.72 (9.36 to 25.13) | 463.45 (262.01 to 730.65) | 0.64 (0.41 to 0.87) | 0.97 (0.79 to 1.15) | 0.37 (0.22 to 0.52) | 0.54 (0.45 to 0.63) |
| Malta | 34.56 (9.17 to 64.03) | 443.57 (128.88 to 750.05) | 3.14 (2.43 to 3.80) | 135.27 (66.18 to 263.11) | 1.07 (0.90 to 1.24) | 0.91 (0.75 to 1.06) | 1.38 (1.03 to 1.74) | 0.65 (0.36 to 0.94) |
| Marshall Islands | 23.01 (5.21 to 47.49) | 357.68 (82.33 to 673.65) | 1.64 (1.01 to 2.56) | 142.18 (47.02 to 271.42) | 0.17 (0.14 to 0.19) | 0.00 (-0.04 to 0.04) | -0.47 (-0.63 to -0.3) | -0.27 (-0.34 to -0.20) |
| Mauritania | 54.29 (13.28 to 110.48) | 688.68 (186.57 to 1232.24) | 10.99 (6.67 to 16.72) | 420.83 (221.18 to 675.34) | 0.54 (0.50 to 0.57) | 0.39 (0.35 to 0.42) | -0.24 (-0.49 to 0.00) | -0.22 (-0.34 to -0.10) |
| Mauritius | 51.03 (14.67 to 97.92) | 855.22 (264.90 to 1426.98) | 11.98 (9.84 to 14.34) | 415.14 (239.93 to 657.89) | 0.95 (0.90 to 1.01) | 0.91 (0.86 to 0.95) | 0.30 (-1.69 to 2.33) | 0.41 (-0.39 to 1.21) |
| Mexico | 53.22 (28.26 to 86.88) | 875.68 (587.60 to 1237.43) | 4.77 (4.14 to 5.43) | 311.35 (205.15 to 455.25) | 0.09 (0.06 to 0.11) | 0.08 (0.05 to 0.12) | -0.95 (-1.81 to -0.07) | -0.51 (-0.72 to -0.30) |
| Micronesia (Federated States of) | 23.56 (4.34 to 45.72) | 365.66 (71.57 to 647.16) | 1.81 (1.10 to 2.86) | 145.72 (47.05 to 275.86) | -0.07 (-0.12 to -0.03) | -0.21 (-0.28 to -0.15) | -1.06 (-1.12 to -1.01) | -0.59 (-0.64 to -0.54) |
| Monaco | 45.06 (13.21 to 83.06) | 608.43 (200.29 to 989.73) | 1.75 (1.08 to 2.66) | 148.04 (52.33 to 317.18) | 0.62 (0.53 to 0.71) | 0.49 (0.41 to 0.57) | 1.80 (1.55 to 2.04) | 0.37 (0.31 to 0.44) |
| Mongolia | 17.23 (3.82 to 35.63) | 449.92 (101.52 to 773.42) | 2.17 (1.45 to 2.98) | 168.89 (59.95 to 319.88) | 0.73 (0.69 to 0.76) | 0.95 (0.88 to 1.03) | -0.56 (-0.72 to -0.4) | 0.05 (-0.09 to 0.19) |
| Montenegro | 14.89 (3.40 to 28.52) | 365.30 (95.98 to 608.68) | 1.62 (1.09 to 2.34) | 105.69 (40.14 to 206.01) | 0.20 (0.13 to 0.27) | -0.05 (-0.10 to -0.01) | 0.01 (-0.44 to 0.47) | -0.32 (-0.46 to -0.18) |
| Morocco | 26.65 (6.45 to 54.10) | 358.79 (89.72 to 654.93) | 6.97 (4.23 to 10.52) | 206.86 (114.14 to 345.43) | 0.63 (0.58 to 0.69) | 0.61 (0.53 to 0.69) | -1.21 (-1.62 to -0.8) | -0.56 (-0.83 to -0.29) |
| Mozambique | 49.08 (9.22 to 106.48) | 614.52 (117.54 to 1242.86) | 38.79 (25.67 to 56.30) | 869.05 (567.08 to 1235.09) | 0.60 (0.55 to 0.65) | 0.85 (0.79 to 0.91) | -0.89 (-0.98 to -0.80) | -0.42 (-0.5 to -0.35) |
| Myanmar | 24.55 (6.03 to 49.32) | 390.72 (100.28 to 714.76) | 2.41 (1.50 to 3.62) | 166.09 (66.78 to 308.19) | 1.05 (0.91 to 1.19) | 1.04 (0.92 to 1.17) | -1.37 (-1.42 to -1.32) | -0.08 (-0.14 to -0.02) |
| Namibia | 48.40 (12.30 to 96.42) | 697.68 (193.66 to 1233.13) | 6.03 (4.11 to 8.51) | 316.49 (148.14 to 569.06) | 0.7 (0.59 to 0.81) | 0.6 (0.57 to 0.64) | -0.36 (-0.42 to -0.3) | -0.05 (-0.1 to 0) |
| Nauru | 21.59 (5.61 to 42.97) | 394.41 (103.06 to 683.90) | 4.50 (2.14 to 8.28) | 192.55 (77.45 to 340.33) | -1.23 (-1.32 to -1.14) | -1.21 (-1.42 to -0.99) | 1.79 (1.71 to 1.88) | -0.61 (-0.71 to -0.51) |
| Nepal | 37.21 (7.83 to 75.34) | 576.17 (118.87 to 1061.02) | 32.99 (10.86 to 55.40) | 640.07 (285.45 to 1012.33) | 1.09 (1.02 to 1.15) | 0.85 (0.78 to 0.92) | -0.34 (-0.42 to -0.26) | -0.33 (-0.41 to -0.25) |
| Netherlands | 46.41 (13.18 to 84.25) | 586.77 (173.46 to 976.01) | 6.20 (5.06 to 7.32) | 205.29 (115.14 to 377.80) | 0.44 (0.4 to 0.48) | -0.08 (-0.13 to -0.04) | 1.51 (0.72 to 2.32) | 0.24 (-0.04 to 0.53) |
| New Zealand | 28.14 (8.33 to 53.46) | 394.57 (125.70 to 649.33) | 2.17 (1.79 to 2.55) | 110.99 (50.03 to 215.02) | 0.1 (0.03 to 0.17) | -0.06 (-0.08 to -0.05) | -0.58 (-1.15 to 0) | -0.59 (-0.79 to -0.39) |
| Nicaragua | 33.64 (7.47 to 68.31) | 522.50 (124.95 to 979.30) | 3.19 (2.24 to 4.41) | 191.96 (82.76 to 367.24) | -0.17 (-0.23 to -0.10) | -0.10 (-0.15 to -0.04) | -0.66 (-0.92 to -0.40) | -0.69 (-0.76 to -0.61) |
| Niger | 32.74 (5.02 to 75.98) | 404.24 (67.97 to 864.10) | 16.21 (8.98 to 29.30) | 459.02 (249.68 to 796.46) | -0.10 (-0.15 to -0.05) | -0.14 (-0.21 to -0.07) | 0.35 (0.16 to 0.53) | 0.07 (-0.10 to 0.24) |
| Nigeria | 55.03 (29.37 to 89.15) | 701.20 (473.36 to 999.30) | 10.27 (6.41 to 13.56) | 401.08 (277.00 to 550.06) | 0.83 (0.78 to 0.89) | 0.72 (0.66 to 0.77) | -0.18 (-0.32 to -0.04) | 0.01 (-0.08 to 0.09) |
| Niue | 31.33 (9.22 to 59.29) | 486.28 (153.61 to 823.24) | 1.13 (0.71 to 1.74) | 155.31 (57.63 to 299.28) | 0.29 (0.23 to 0.35) | 0.18 (0.15 to 0.22) | -1.05 (-1.16 to -0.94) | -0.28 (-0.34 to -0.22) |
| North Macedonia | 19.47 (5.26 to 37.43) | 435.58 (124.91 to 736.85) | 3.60 (2.02 to 5.56) | 160.93 (78.90 to 284.80) | 0.53 (0.49 to 0.58) | 0.14 (0.07 to 0.21) | 0.48 (0.11 to 0.85) | -0.14 (-0.29 to 0.02) |
| Northern Mariana Islands | 33.65 (7.33 to 62.12) | 533.49 (138.53 to 876.71) | 0.55 (0.34 to 0.83) | 152.89 (41.96 to 307.56) | -0.51 (-0.58 to -0.45) | -0.64 (-0.75 to -0.53) | 0.28 (-0.35 to 0.92) | -0.84 (-1.03 to -0.64) |
| Norway | 48.16 (23.56 to 81.02) | 678.63 (390.68 to 996.68) | 5.32 (4.56 to 5.90) | 215.34 (136.51 to 338.16) | 0.91 (0.64 to 1.18) | 0.52 (0.37 to 0.67) | 0.65 (-0.05 to 1.36) | 0.23 (-0.19 to 0.65) |
| Oman | 37.45 (9.79 to 69.76) | 442.85 (121.36 to 738.59) | 1.43 (0.89 to 2.08) | 129.31 (46.99 to 251.15) | 2.07 (1.85 to 2.29) | 1.38 (1.30 to 1.46) | -0.77 (-1.68 to 0.16) | 0.30 (0.12 to 0.48) |
| Pakistan | 25.65 (10.84 to 45.93) | 402.73 (196.49 to 641.11) | 18.84 (10.71 to 29.15) | 417.28 (273.13 to 600.53) | 0.23 (0.20 to 0.26) | -0.03 (-0.07 to 0.01) | 0.38 (0.34 to 0.43) | 0.02 (-0.06 to 0.09) |
| Palau | 32.57 (8.25 to 61.86) | 534.63 (146.13 to 883.22) | 1.59 (0.99 to 2.48) | 175.68 (59.27 to 327.98) | 0.06 (0.01 to 0.12) | -0.08 (-0.11 to -0.05) | -0.79 (-1.10 to -0.48) | -0.45 (-0.5 to -0.40) |
| Palestine | 26.12 (7.18 to 50.01) | 320.79 (93.11 to 557.81) | 6.33 (4.30 to 8.80) | 175.62 (100.92 to 278.97) | 0.81 (0.71 to 0.91) | 0.55 (0.47 to 0.63) | -1.54 (-1.73 to -1.35) | -0.94 (-1.02 to -0.87) |
| Panama | 53.53 (14.70 to 100.43) | 851.58 (246.64 to 1405.09) | 3.90 (2.92 to 4.92) | 277.02 (115.87 to 515.87) | 0.93 (0.91 to 0.96) | 1.02 (0.99 to 1.04) | -0.28 (-1.02 to 0.46) | 0.26 (0.06 to 0.47) |
| Papua New Guinea | 18.10 (3.91 to 37.50) | 286.50 (67.18 to 538.88) | 1.92 (0.95 to 3.45) | 126.06 (43.67 to 238.76) | -0.08 (-0.13 to -0.03) | -0.06 (-0.10 to -0.02) | 0.49 (0.41 to 0.58) | -0.02 (-0.06 to 0.02) |
| Paraguay | 34.58 (9.67 to 68.19) | 559.82 (177.00 to 967.03) | 3.81 (2.26 to 5.67) | 215.03 (99.26 to 386.59) | 0.59 (0.53 to 0.65) | 0.52 (0.47 to 0.56) | 0.52 (0.26 to 0.79) | 0.15 (0.00 to 0.30) |
| Peru | 40.44 (10.45 to 77.90) | 693.45 (199.54 to 1198.03) | 1.62 (1.06 to 2.32) | 203.24 (67.86 to 417.37) | 0.44 (0.39 to 0.49) | 0.16 (0.04 to 0.27) | -2.42 (-2.97 to -1.86) | -1.06 (-1.15 to -0.97) |
| Philippines | 28.42 (14.97 to 45.23) | 384.42 (259.00 to 545.34) | 1.26 (0.93 to 1.60) | 138.37 (88.72 to 205.59) | 0.38 (0.27 to 0.49) | 0.34 (0.30 to 0.37) | -0.22 (-0.53 to 0.09) | 0.15 (0.08 to 0.22) |
| Poland | 13.75 (6.79 to 22.41) | 399.91 (256.38 to 551.19) | 5.15 (4.30 to 5.84) | 178.08 (131.57 to 241.54) | 0.58 (0.53 to 0.62) | 0.84 (0.80 to 0.88) | 4.22 (3.88 to 4.57) | 1.55 (1.24 to 1.87) |
| Portugal | 42.91 (11.53 to 79.57) | 504.86 (143.16 to 839.03) | 7.66 (6.09 to 9.10) | 211.52 (127.92 to 364.76) | 1.69 (1.55 to 1.82) | 1.50 (1.36 to 1.63) | 2.84 (2.10 to 3.59) | 1.62 (1.29 to 1.95) |
| Puerto Rico | 41.64 (10.79 to 79.34) | 676.93 (192.00 to 1136.37) | 3.32 (2.59 to 4.09) | 206.70 (85.85 to 399.03) | -0.32 (-0.38 to -0.26) | -0.55 (-0.63 to -0.47) | -1.97 (-2.71 to -1.23) | -1.40 (-1.81 to -0.98) |
| Qatar | 37.82 (10.70 to 71.56) | 533.40 (166.30 to 886.26) | 2.85 (1.49 to 4.32) | 159.28 (70.12 to 305.50) | 0.40 (0.28 to 0.52) | 0.19 (0.08 to 0.31) | -2.99 (-3.73 to -2.25) | -1.47 (-1.79 to -1.14) |
| Republic of Korea | 47.71 (12.05 to 90.01) | 602.80 (153.49 to 997.26) | 3.09 (1.67 to 4.29) | 166.75 (65.74 to 339.83) | 1.14 (1.03 to 1.25) | 0.89 (0.79 to 1.00) | -0.33 (-0.54 to -0.12) | -0.21 (-0.29 to -0.13) |
| Republic of Moldova | 11.94 (3.28 to 23.62) | 263.77 (82.35 to 454.56) | 2.19 (1.70 to 2.71) | 102.67 (53.09 to 184.44) | 0.30 (0.28 to 0.32) | -0.14 (-0.17 to -0.12) | -1.23 (-1.96 to -0.49) | -0.76 (-1.00 to -0.52) |
| Romania | 15.19 (4.39 to 28.87) | 429.99 (129.29 to 713.44) | 3.32 (2.58 to 4.04) | 156.63 (78.02 to 280.16) | 0.58 (0.47 to 0.70) | 0.44 (0.33 to 0.55) | 1.32 (0.31 to 2.34) | 0.31 (-0.09 to 0.72) |
| Russian Federation | 17.79 (9.15 to 28.92) | 323.48 (212.74 to 464.64) | 1.70 (1.47 to 1.92) | 96.66 (61.00 to 150.98) | 0.66 (0.56 to 0.76) | 0.20 (0.10 to 0.29) | 1.64 (0.30 to 3.01) | -0.02 (-0.25 to 0.20) |
| Rwanda | 45.37 (9.76 to 91.61) | 546.42 (120.38 to 1029.98) | 31.37 (20.23 to 47.49) | 693.68 (438.99 to 1026.51) | 0.50 (0.37 to 0.64) | 0.19 (0.10 to 0.29) | -0.94 (-1.07 to -0.80) | -0.79 (-0.94 to -0.64) |
| Saint Kitts and Nevis | 51.41 (14.15 to 103.07) | 876.10 (257.81 to 1464.17) | 7.36 (5.48 to 9.28) | 356.10 (174.01 to 632.86) | 0.13 (0.09 to 0.17) | -0.12 (-0.21 to -0.03) | -1.53 (-2.66 to -0.38) | -1.05 (-1.61 to -0.49) |
| Saint Lucia | 39.92 (9.66 to 77.81) | 648.93 (179.15 to 1139.93) | 7.12 (5.53 to 8.88) | 285.26 (148.17 to 493.10) | -0.23 (-0.30 to -0.17) | -0.33 (-0.40 to -0.25) | -1.46 (-2.65 to -0.25) | -1.05 (-1.28 to -0.82) |
| Saint Vincent and the Grenadines | 39.00 (9.88 to 76.80) | 627.32 (162.78 to 1099.64) | 7.24 (5.99 to 8.67) | 292.87 (152.68 to 477.09) | 0.45 (0.40 to 0.50) | 0.40 (0.38 to 0.43) | -0.68 (-1.82 to 0.46) | -0.37 (-0.58 to -0.17) |
| Samoa | 24.44 (6.73 to 47.67) | 386.93 (111.69 to 654.78) | 1.49 (0.89 to 2.25) | 140.20 (49.05 to 267.06) | -0.05 (-0.16 to 0.05) | -0.08 (-0.11 to -0.05) | -0.55 (-0.60 to -0.49) | -0.35 (-0.45 to -0.26) |
| San Marino | 25.80 (7.07 to 46.72) | 367.14 (109.04 to 606.52) | 0.46 (0.26 to 0.72) | 79.34 (21.72 to 183.15) | -0.26 (-0.34 to -0.17) | -0.28 (-0.39 to -0.17) | -2.12 (-2.47 to -1.77) | -0.69 (-0.87 to -0.51) |
| Sao Tome and Principe | 53.42 (14.09 to 102.99) | 705.62 (196.35 to 1236.39) | 10.36 (6.54 to 15.31) | 403.76 (216.96 to 650.70) | 0.60 (0.56 to 0.64) | 0.86 (0.79 to 0.93) | 0.02 (-0.14 to 0.19) | 0.23 (0.13 to 0.34) |
| Saudi Arabia | 47.11 (12.01 to 90.88) | 714.78 (200.71 to 1160.29) | 4.51 (3.08 to 6.25) | 242.93 (106.72 to 444.11) | 0.24 (0.20 to 0.28) | 0.42 (0.38 to 0.46) | -1.50 (-1.60 to -1.40) | -0.78 (-0.83 to -0.72) |
| Senegal | 51.87 (11.12 to 109.94) | 678.25 (156.42 to 1310.95) | 13.67 (8.39 to 21.17) | 485.47 (260.70 to 787.28) | 0.65 (0.58 to 0.72) | 0.72 (0.63 to 0.81) | 0.22 (-0.08 to 0.53) | 0.35 (0.27 to 0.43) |
| Serbia | 26.17 (7.17 to 48.95) | 563.26 (165.82 to 932.32) | 3.86 (2.00 to 5.60) | 190.79 (81.23 to 358.86) | 0.88 (0.77 to 0.99) | 0.50 (0.45 to 0.55) | -0.87 (-1.38 to -0.36) | -0.32 (-0.49 to -0.14) |
| Seychelles | 32.96 (9.00 to 65.38) | 499.87 (142.40 to 871.73) | 3.46 (2.18 to 5.21) | 195.74 (85.74 to 353.73) | 0.59 (0.42 to 0.76) | 0.46 (0.26 to 0.65) | -0.91 (-1.08 to -0.74) | -0.34 (-0.63 to -0.04) |
| Sierra Leone | 40.71 (8.05 to 83.20) | 518.71 (108.61 to 999.01) | 12.33 (7.36 to 19.32) | 414.26 (219.95 to 667.13) | 0.04 (-0.14 to 0.22) | -0.01 (-0.19 to 0.18) | 0.10 (0.00 to 0.2) | -0.09 (-0.22 to 0.04) |
| Singapore | 30.70 (8.00 to 58.12) | 435.59 (126.92 to 727.94) | 1.46 (1.14 to 1.78) | 109.23 (40.24 to 245.29) | 0.81 (0.76 to 0.85) | 0.69 (0.67 to 0.71) | 1.23 (0.43 to 2.04) | 0.13 (-0.06 to 0.32) |
| Slovakia | 18.61 (4.57 to 36.60) | 545.68 (149.63 to 905.90) | 3.48 (2.38 to 4.79) | 181.27 (84.73 to 330.44) | 0.65 (0.58 to 0.71) | 0.50 (0.45 to 0.54) | -0.21 (-0.38 to -0.03) | -0.07 (-0.15 to 0.02) |
| Slovenia | 20.19 (5.54 to 37.83) | 498.74 (146.23 to 821.25) | 4.23 (3.07 to 5.45) | 171.20 (90.42 to 305.14) | 0.10 (0.06 to 0.14) | -0.51 (-0.56 to -0.45) | 1.99 (1.42 to 2.57) | -0.21 (-0.53 to 0.12) |
| Solomon Islands | 18.59 (4.12 to 39.03) | 300.16 (69.05 to 561.95) | 2.49 (1.28 to 4.36) | 137.50 (50.42 to 265.69) | 0.17 (-0.06 to 0.40) | -0.01 (-0.15 to 0.12) | -0.09 (-0.19 to 0.01) | -0.29 (-0.35 to -0.23) |
| Somalia | 26.46 (3.96 to 62.34) | 286.80 (42.26 to 630.74) | 40.47 (23.19 to 68.55) | 800.92 (468.34 to 1294.89) | -0.21 (-0.24 to -0.18) | -0.47 (-0.5 to -0.44) | -0.51 (-0.60 to -0.42) | -0.35 (-0.42 to -0.27) |
| South Africa | 51.36 (25.47 to 84.49) | 680.88 (416.00 to 1008.94) | 3.87 (2.89 to 4.68) | 257.62 (164.66 to 384.28) | 0.65 (0.59 to 0.71) | 0.39 (0.28 to 0.50) | -0.28 (-0.53 to -0.03) | -0.04 (-0.13 to 0.06) |
| South Sudan | 44.04 (8.96 to 93.64) | 535.94 (117.87 to 1054.65) | 40.69 (24.46 to 62.89) | 825.10 (510.60 to 1235.14) | -0.39 (-0.43 to -0.34) | -0.56 (-0.64 to -0.48) | -0.73 (-0.89 to -0.57) | -0.89 (-1.01 to -0.77) |
| Spain | 36.75 (10.07 to 69.13) | 457.99 (141.85 to 785.83) | 4.95 (3.92 to 5.91) | 160.49 (88.00 to 294.00) | 1.42 (1.34 to 1.50) | 1.06 (0.93 to 1.18) | 3.07 (2.4 to 3.74) | 1.35 (0.94 to 1.76) |
| Sri Lanka | 55.73 (12.99 to 110.24) | 816.90 (194.77 to 1427.82) | 6.32 (3.85 to 9.47) | 301.62 (131.28 to 540.65) | 0.66 (0.63 to 0.68) | 0.59 (0.57 to 0.61) | -2.46 (-2.82 to -2.10) | -1.08 (-1.23 to -0.92) |
| Sudan | 20.36 (5.11 to 40.34) | 263.23 (69.13 to 489.47) | 3.39 (2.09 to 5.54) | 132.68 (67.89 to 229.19) | 0.80 (0.73 to 0.87) | 0.72 (0.64 to 0.81) | -1.29 (-1.38 to -1.2) | -0.52 (-0.57 to -0.46) |
| Suriname | 40.22 (11.69 to 78.95) | 684.17 (220.29 to 1155.70) | 5.49 (3.53 to 8.13) | 293.01 (144.30 to 518.24) | 0.32 (0.17 to 0.48) | 0.27 (0.20 to 0.34) | -1.04 (-1.41 to -0.67) | -0.51 (-0.64 to -0.39) |
| Sweden | 24.43 (8.16 to 47.24) | 351.51 (140.29 to 569.59) | 2.36 (1.90 to 2.82) | 105.62 (55.39 to 195.11) | 0.66 (0.55 to 0.77) | 0.28 (0.21 to 0.35) | 0.62 (0.22 to 1.02) | 0.08 (-0.13 to 0.29) |
| Switzerland | 48.98 (12.66 to 91.07) | 598.91 (171.98 to 999.12) | 5.52 (4.26 to 6.73) | 197.75 (103.33 to 361.56) | 0.00 (-0.09 to 0.09) | -0.16 (-0.22 to -0.09) | 0.78 (0.08 to 1.48) | -0.11 (-0.36 to 0.13) |
| Syrian Arab Republic | 16.93 (4.12 to 32.40) | 227.02 (59.94 to 389.46) | 2.12 (1.40 to 3.07) | 90.01 (43.40 to 163.95) | 0.57 (0.41 to 0.73) | 0.32 (0.18 to 0.45) | -1.66 (-1.97 to -1.36) | -0.91 (-1.03 to -0.79) |
| Taiwan (Province of China) | 40.77 (10.21 to 71.29) | 605.67 (159.04 to 942.73) | 2.81 (2.22 to 3.36) | 179.88 (75.24 to 351.54) | 0.23 (0.04 to 0.41) | 0.23 (0.04 to 0.42) | 2.58 (1.32 to 3.86) | 0.10 (-0.32 to 0.52) |
| Tajikistan | 27.40 (6.91 to 54.80) | 553.61 (145.29 to 959.42) | 3.08 (2.04 to 4.54) | 226.63 (90.76 to 416.11) | 0.96 (0.89 to 1.03) | 0.26 (0.22 to 0.30) | -1.23 (-1.97 to -0.49) | -0.25 (-0.44 to -0.06) |
| Thailand | 38.67 (11.01 to 74.99) | 556.49 (169.23 to 950.25) | 3.68 (2.48 to 5.36) | 199.61 (89.85 to 366.02) | 1.72 (1.69 to 1.76) | 1.62 (1.58 to 1.66) | -0.86 (-1.13 to -0.58) | 0.34 (0.27 to 0.41) |
| Timor-Leste | 27.60 (7.06 to 55.23) | 397.18 (109.90 to 720.92) | 2.15 (1.07 to 4.61) | 163.30 (58.14 to 311.98) | 0.95 (0.74 to 1.15) | 0.91 (0.72 to 1.11) | -0.56 (-0.74 to -0.38) | 0.23 (0.17 to 0.28) |
| Togo | 48.17 (9.51 to 102.01) | 616.41 (129.34 to 1187.38) | 11.69 (6.99 to 18.17) | 422.20 (220.80 to 690.87) | 0.17 (0.08 to 0.26) | -0.18 (-0.27 to -0.09) | 0.11 (-0.14 to 0.36) | -0.21 (-0.35 to -0.08) |
| Tokelau | 23.50 (5.40 to 46.02) | 361.61 (88.09 to 627.91) | 1.82 (1.07 to 2.89) | 132.96 (47.69 to 252.88) | 0.11 (0.00 to 0.22) | 0.01 (-0.16 to 0.18) | -0.18 (-0.71 to 0.36) | -0.35 (-0.59 to -0.12) |
| Tonga | 25.26 (7.01 to 47.86) | 376.28 (105.63 to 646.66) | 1.22 (0.76 to 1.88) | 130.35 (42.91 to 251.00) | 0.31 (0.25 to 0.37) | 0.19 (0.16 to 0.23) | -0.82 (-1.03 to -0.61) | -0.17 (-0.22 to -0.12) |
| Trinidad and Tobago | 49.93 (12.82 to 96.34) | 824.68 (223.51 to 1378.05) | 5.27 (3.93 to 6.80) | 311.96 (145.75 to 577.90) | 0.10 (0.02 to 0.19) | 0.21 (0.11 to 0.30) | -1.65 (-2.27 to -1.03) | -0.74 (-1.03 to -0.44) |
| Tunisia | 24.16 (6.41 to 46.69) | 308.14 (90.00 to 551.80) | 3.03 (1.74 to 5.53) | 117.80 (54.65 to 217.98) | 0.72 (0.65 to 0.79) | 0.71 (0.65 to 0.77) | -0.68 (-0.76 to -0.6) | -0.42 (-0.48 to -0.36) |
| Turkey | 42.67 (11.31 to 80.47) | 563.12 (163.41 to 958.62) | 6.25 (4.13 to 8.58) | 229.96 (124.51 to 392.70) | 1.73 (1.54 to 1.91) | 1.31 (1.14 to 1.47) | -0.28 (-0.94 to 0.39) | -0.04 (-0.44 to 0.37) |
| Turkmenistan | 18.68 (4.22 to 36.41) | 450.33 (106.75 to 757.34) | 2.24 (1.65 to 3.00) | 167.97 (68.31 to 304.71) | 0.51 (0.49 to 0.54) | 0.69 (0.66 to 0.72) | 0.92 (0.31 to 1.53) | 0.42 (0.25 to 0.60) |
| Tuvalu | 22.80 (6.00 to 43.19) | 348.54 (100.62 to 594.92) | 1.53 (0.89 to 2.45) | 132.86 (49.97 to 249.47) | 0.21 (0.13 to 0.30) | 0.07 (0 to 0.15) | -0.70 (-0.76 to -0.63) | -0.42 (-0.48 to -0.36) |
| Uganda | 50.40 (10.17 to 101.29) | 606.57 (126.36 to 1151.33) | 26.14 (17.14 to 39.10) | 628.38 (396.49 to 922.56) | 0.73 (0.63 to 0.83) | 0.70 (0.61 to 0.79) | -0.93 (-1.08 to -0.79) | -0.53 (-0.62 to -0.45) |
| Ukraine | 12.66 (3.48 to 24.48) | 254.87 (81.58 to 438.39) | 0.29 (0.21 to 0.39) | 67.77 (21.45 to 138.73) | 0.25 (0.17 to 0.32) | -0.11 (-0.15 to -0.06) | -1.09 (-2.53 to 0.36) | -0.4 (-0.62 to -0.18) |
| United Arab Emirates | 52.62 (14.93 to 101.92) | 690.44 (213.39 to 1156.24) | 3.87 (2.38 to 5.78) | 243.15 (108.17 to 450.09) | 0.29 (0.18 to 0.40) | -0.37 (-0.49 to -0.25) | -1.46 (-3.64 to 0.77) | -1.00 (-1.8 to -0.2) |
| United Kingdom | 40.78 (21.37 to 65.67) | 548.04 (347.58 to 778.91) | 3.02 (2.62 to 3.28) | 157.56 (99.64 to 250.66) | -0.01 (-0.09 to 0.08) | 0.15 (0.02 to 0.27) | -0.65 (-1.53 to 0.24) | -0.36 (-0.64 to -0.07) |
| United Republic of Tanzania | 50.43 (12.08 to 104.02) | 622.46 (154.74 to 1178.52) | 31.56 (20.24 to 46.91) | 719.21 (454.61 to 1062.95) | 0.58 (0.27 to 0.89) | 0.43 (0.03 to 0.83) | -1.31 (-1.39 to -1.23) | -0.87 (-1.00 to -0.73) |
| United States of America | 37.49 (18.70 to 61.68) | 545.02 (330.56 to 786.99) | 2.51 (2.18 to 2.74) | 150.05 (91.86 to 239.49) | 0.55 (0.44 to 0.66) | 1.67 (1.45 to 1.89) | 1.65 (1.21 to 2.08) | 1.44 (1.18 to 1.71) |
| United States Virgin Islands | 45.36 (12.62 to 87.00) | 761.53 (224.57 to 1237.18) | 1.74 (1.13 to 2.55) | 221.11 (69.98 to 429.68) | 1.77 (1.65 to 1.90) | 0.49 (0.40 to 0.58) | -2.99 (-3.52 to -2.45) | -0.62 (-0.80 to -0.44) |
| Uruguay | 34.92 (7.94 to 66.91) | 546.93 (132.07 to 933.75) | 4.60 (3.89 to 5.35) | 204.35 (98.82 to 370.90) | 1.13 (1.06 to 1.19) | 1.31 (1.22 to 1.40) | 0.92 (-0.06 to 1.91) | 0.80 (0.43 to 1.17) |
| Uzbekistan | 22.43 (6.14 to 43.66) | 557.89 (157.04 to 942.80) | 3.03 (2.31 to 3.83) | 212.46 (89.75 to 391.83) | 1.47 (1.40 to 1.55) | 1.44 (1.33 to 1.56) | 1.16 (-0.06 to 2.38) | 1.13 (0.75 to 1.52) |
| Vanuatu | 20.51 (4.07 to 40.86) | 316.49 (67.45 to 566.62) | 1.35 (0.77 to 2.13) | 127.23 (37.32 to 248.73) | 0.03 (0.01 to 0.05) | -0.11 (-0.14 to -0.08) | -0.42 (-0.51 to -0.33) | -0.28 (-0.32 to -0.23) |
| Venezuela (Bolivarian Republic of) | 39.25 (10.22 to 80.45) | 683.14 (191.02 to 1227.94) | 4.58 (3.38 to 5.92) | 261.87 (112.73 to 492.03) | -0.22 (-0.29 to -0.14) | -0.31 (-0.39 to -0.24) | 0.00 (-0.86 to 0.86) | -0.63 (-0.82 to -0.43) |
| Viet Nam | 26.48 (5.52 to 52.64) | 390.18 (85.51 to 685.77) | 0.25 (0.04 to 1.07) | 110.84 (24.23 to 232.76) | 1.43 (1.36 to 1.50) | 1.49 (1.42 to 1.57) | -0.77 (-0.93 to -0.60) | 0.82 (0.74 to 0.90) |
| Yemen | 17.20 (3.02 to 34.77) | 220.23 (37.79 to 405.69) | 3.87 (2.32 to 6.53) | 132.03 (65.30 to 222.40) | 0.37 (0.33 to 0.40) | 0.14 (0.10 to 0.19) | -0.83 (-0.97 to -0.68) | -0.60 (-0.67 to -0.53) |
| Zambia | 60.02 (14.35 to 118.70) | 800.82 (216.71 to 1438.28) | 46.76 (30.15 to 67.90) | 1059.23 (693.98 to 1516.42) | 0.39 (0.36 to 0.43) | 0.36 (0.31 to 0.41) | 0.12 (-0.12 to 0.37) | 0.29 (0.12 to 0.47) |
| Zimbabwe | 47.73 (11.19 to 98.68) | 664.76 (164.73 to 1219.15) | 12.21 (7.07 to 19.09) | 438.85 (219.82 to 719.74) | -0.03 (-0.21 to 0.15) | -0.21 (-0.29 to -0.12) | -0.27 (-0.40 to -0.14) | -0.21 (-0.27 to -0.16) |
