# Supplementary material for: Global, regional, and national burdens of late-onset epilepsy in adults aged 65 years and older from 1990 to 2021: A population-based study
Source: PLoS One. 2025 Nov 19;20(11):e0336588. doi: 10.1371/journal.pone.0336588 (PMC12629476; doi:10.1371/journal.pone.0336588)
Supplement: S1 Fig — Abbreviations: AAPC, average annual percentage change. (PDF) [file pone.0336588.s006.pdf]

$$\text{Age standardised rate} = \frac{\sum_{i=1}^{A} a_i w_i}{\sum_{i=1}^{A} w_i}$$

where a_i is the age specific rate and w_i is the weight in the same age subgroup of the chosen reference standard population (in which i denotes the ith age class) and A is the upper age limit

$$\text{AAPC} = \left\{\exp\left(\frac{\sum w_i b_i}{\sum w_i}\right) - 1\right\} \times 100$$

b_i is the slope coefficient for the ith segment with i indexing the segments in the desired range of years, and w_i is the length of each segment in the range of years
