# Supplementary figures and images for: Global, regional, and national burdens of late-onset epilepsy in adults aged 65 years and older from 1990 to 2021: A population-based study
Source: PLoS One. 2025 Nov 19;20(11):e0336588. doi: 10.1371/journal.pone.0336588 (PMC12629476; doi:10.1371/journal.pone.0336588)

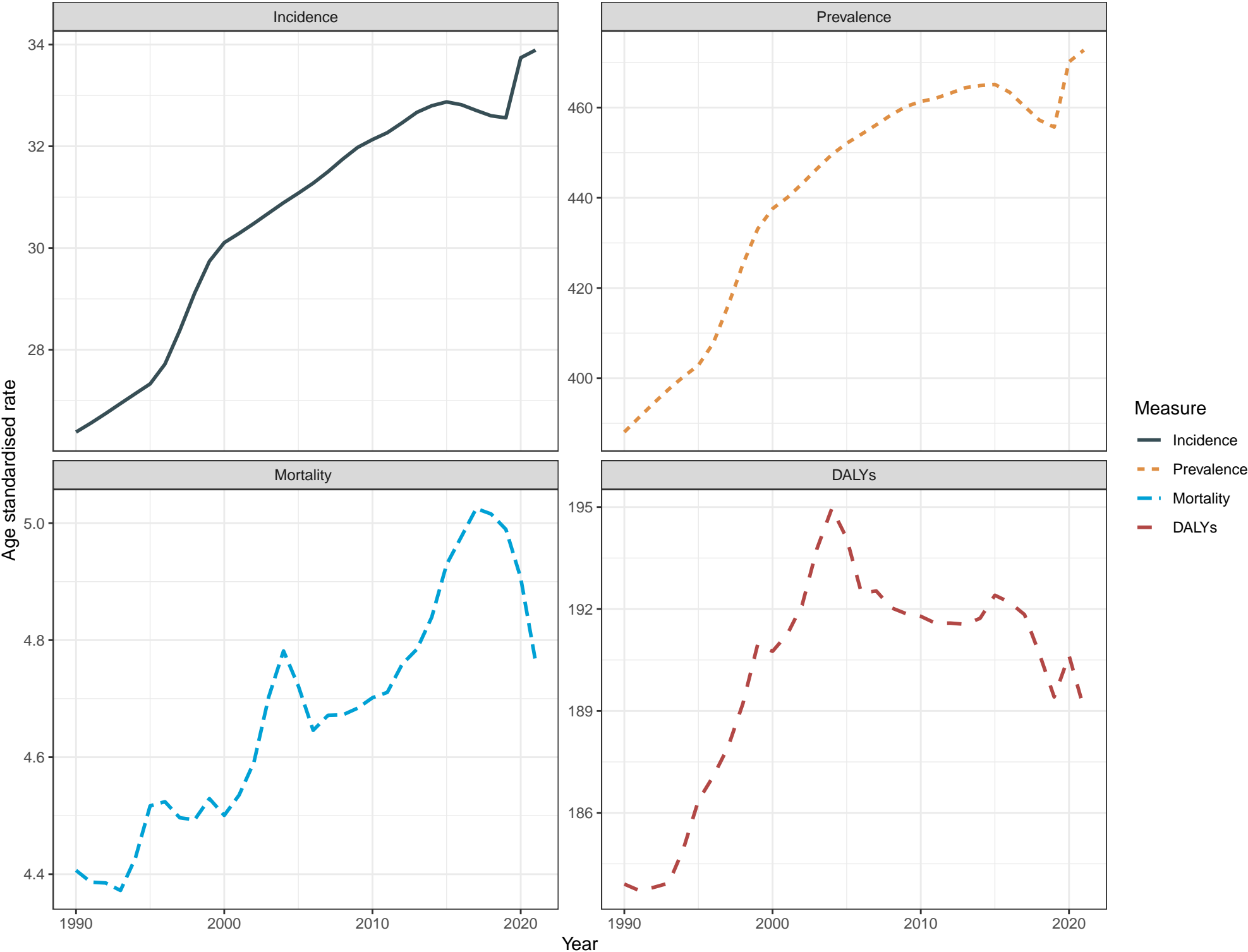

Supplement: S2 Fig — Abbreviations: ASIR, age-standardized incidence rate; ASPR, age-standardized prevalence rate; ASMR, age-standardized mortality rate; DALYs, disability-adjusted life-years; LOE, late-onset epilepsy. (PDF) [file pone.0336588.s007.pdf]

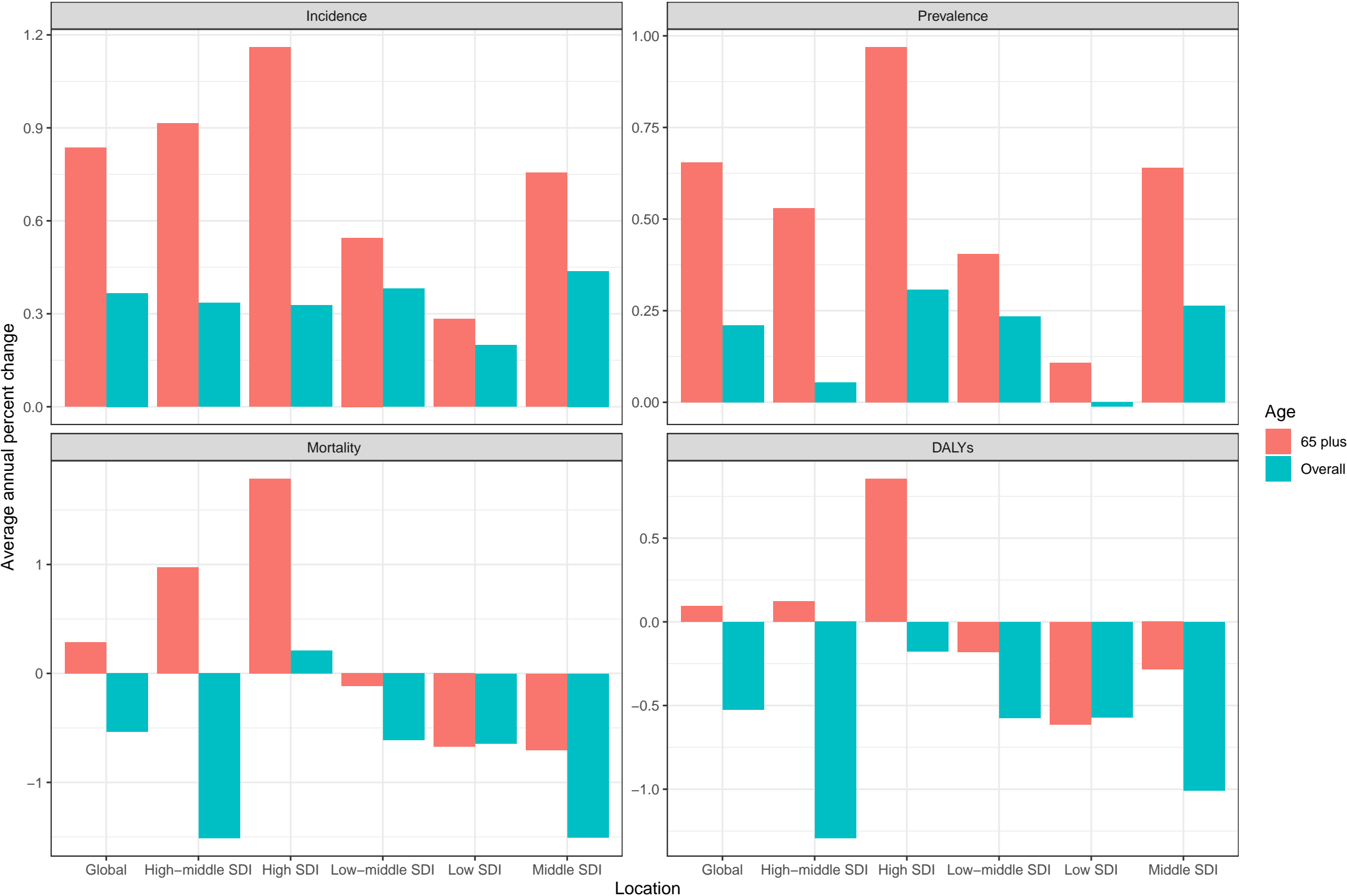

Supplement: S3 Fig — Abbreviations: ASIR, age-standardized incidence rate; ASPR, age-standardized prevalence rate; ASMR, age-standardized mortality rate; DALYs, disability-adjusted life-years; SDI, sociodemographic index; LOE, late-onset epilepsy. (PDF) [file pone.0336588.s008.pdf]

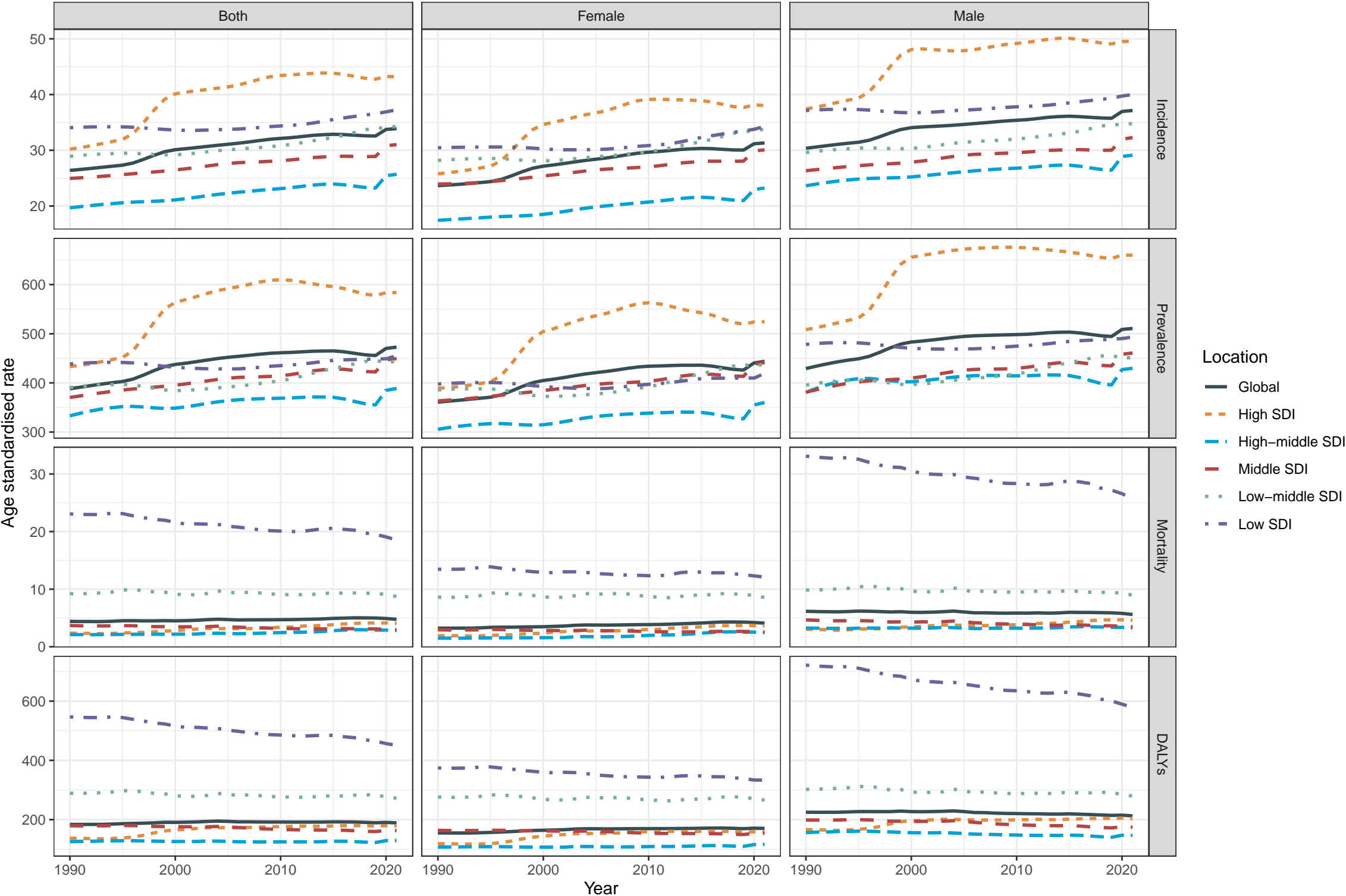

Supplement: S4 Fig — Abbreviations: ASIR, age-standardized incidence rate; ASPR, age-standardized prevalence rate; ASMR, age-standardized mortality rate; DALYs, disability-adjusted life-years; SDI, sociodemographic index; LOE, late-onset epilepsy. (PDF) [file pone.0336588.s009.pdf]

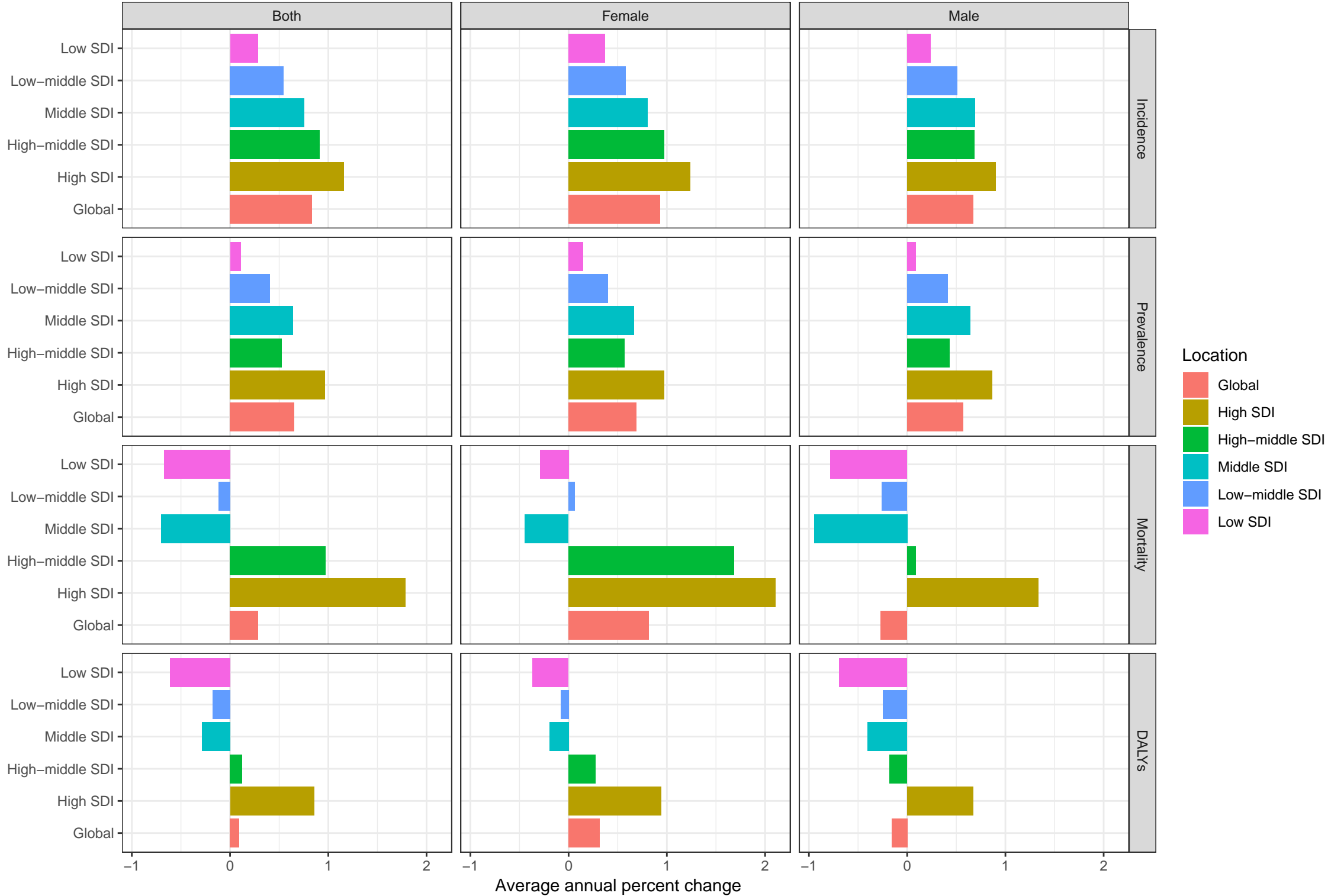

Supplement: S5 Fig — Abbreviations: ASIR, age-standardized incidence rate; ASPR, age-standardized prevalence rate; ASMR, age-standardized mortality rate; DALYs, disability-adjusted life-years; SDI, sociodemographic index; LOE, late-onset epilepsy. (PDF) [file pone.0336588.s010.pdf]

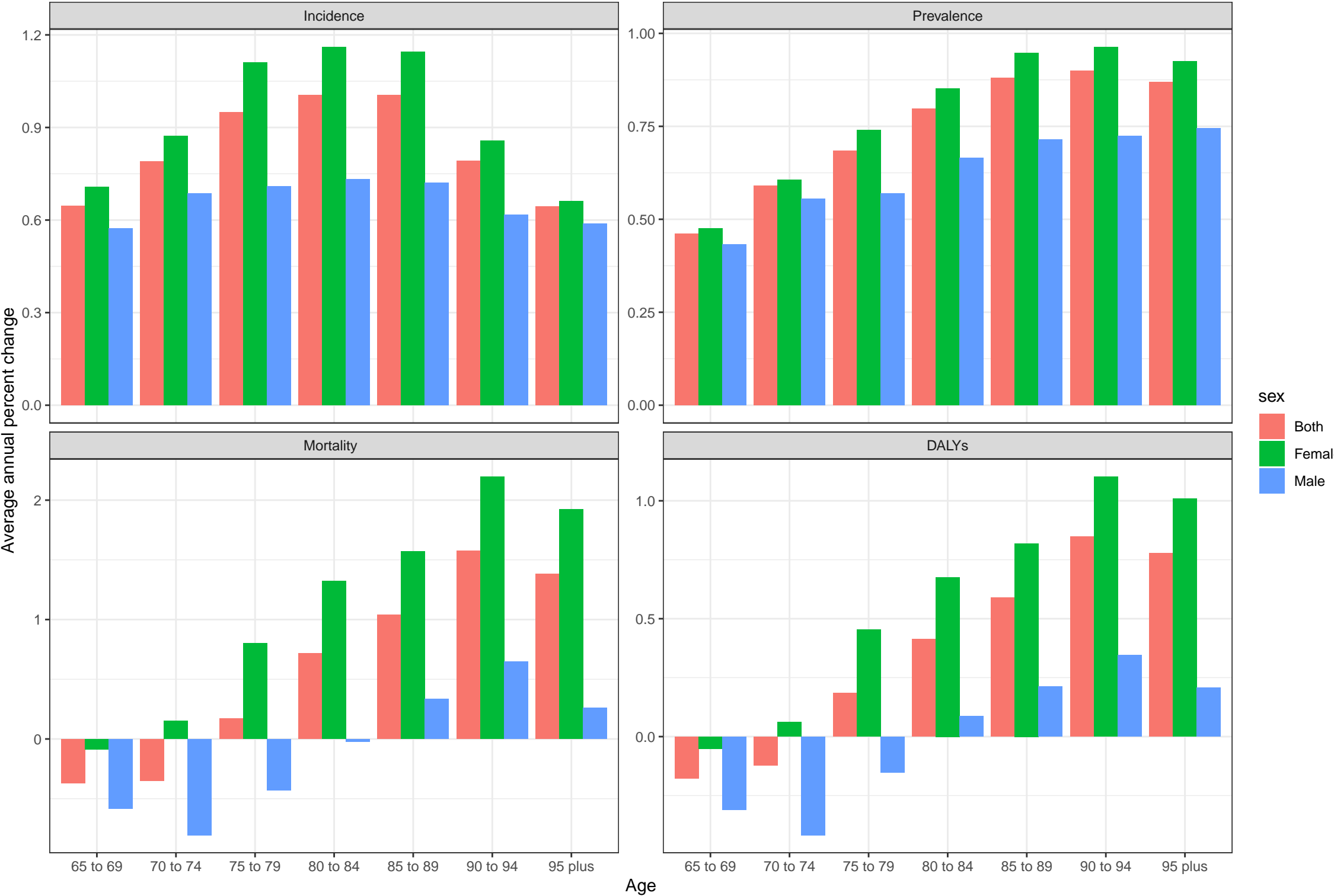

Supplement: S6 Fig — Abbreviations: ASIR, age-standardized incidence rate; ASPR, age-standardized prevalence rate; ASMR, age-standardized mortality rate; DALYs, disability-adjusted life-years; LOE, late-onset epilepsy. (PDF) [file pone.0336588.s011.pdf]

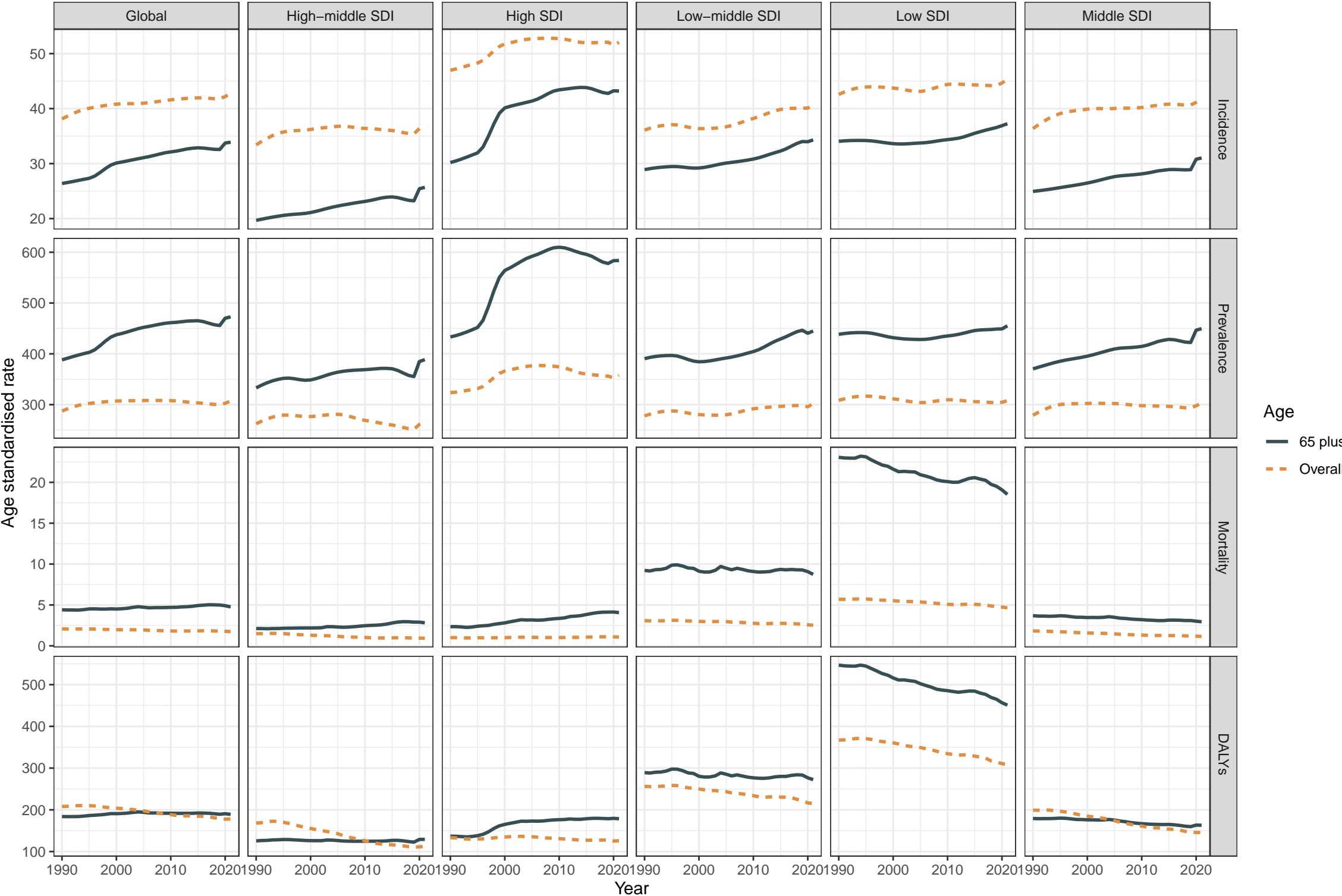

Supplement: S7 Fig — Abbreviations: ASIR, age-standardized incidence rate; ASPR, age-standardized prevalence rate; ASMR, age-standardized mortality rate; DALYs, disability-adjusted life-years; SDI, sociodemographic index; LOE, late-onset epilepsy. (PDF) [file pone.0336588.s012.pdf]

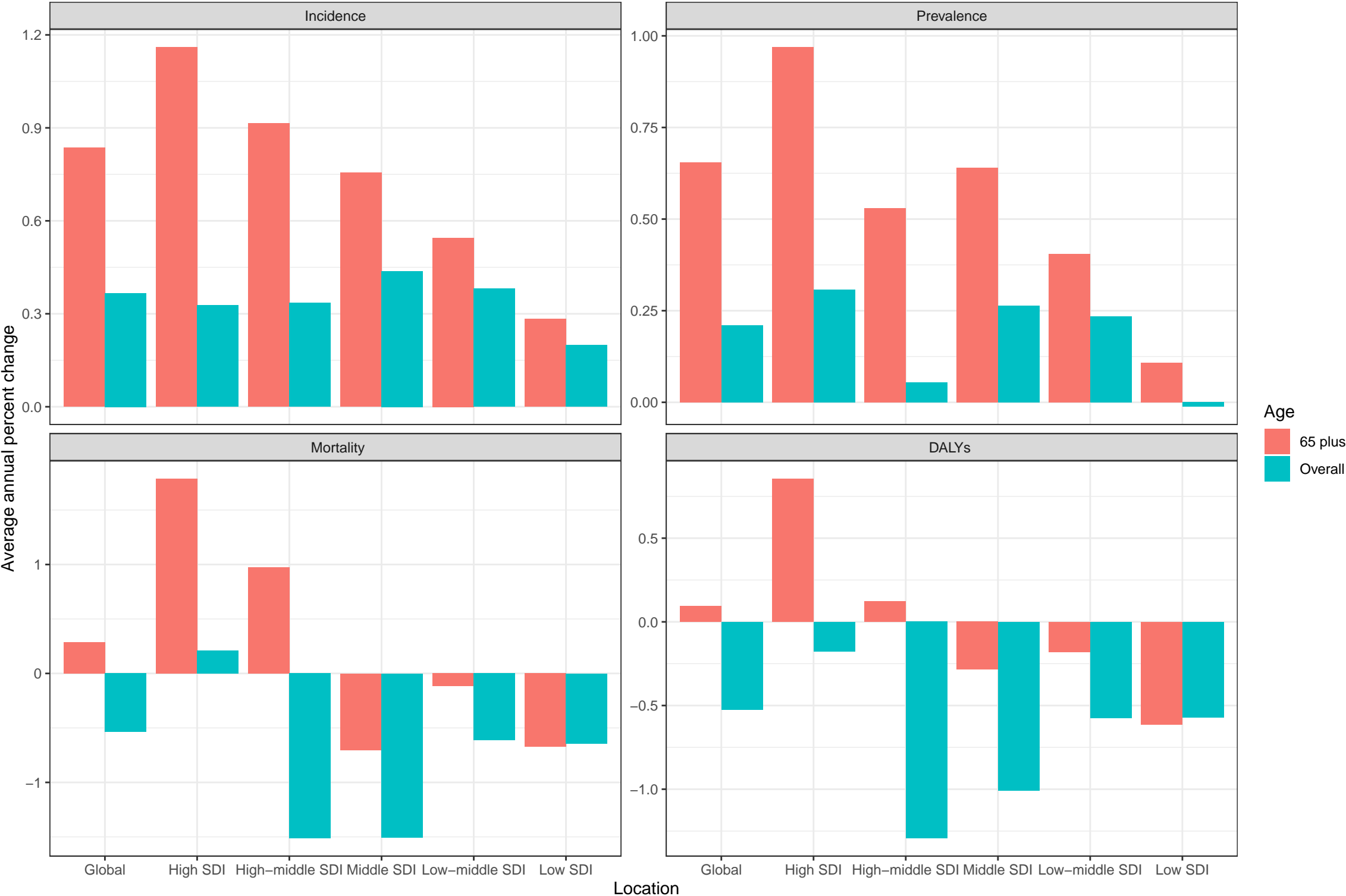

Supplement: S8 Fig — Abbreviations: ASIR, age-standardized incidence rate; ASPR, age-standardized prevalence rate; ASMR, age-standardized mortality rate; DALYs, disability-adjusted life-years; SDI, sociodemographic index; LOE, late-onset epilepsy. (PDF) [file pone.0336588.s013.pdf]

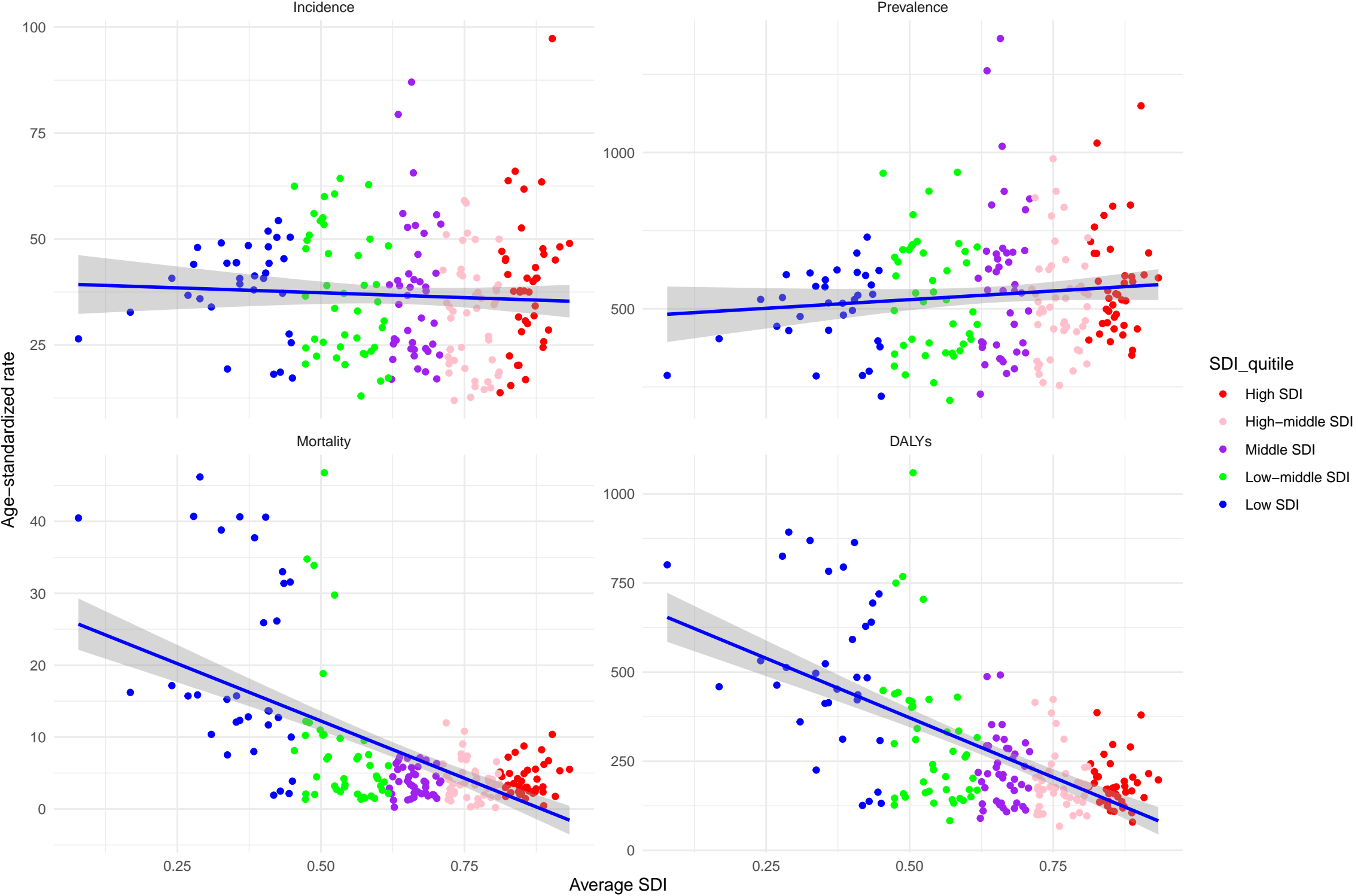

Supplement: S9 Fig — Abbreviations: ASIR, age-standardized incidence rate; ASPR, age-standardized prevalence rate; ASMR, age-standardized mortality rate; DALYs, disability-adjusted life-years; SDI, sociodemographic index; LOE, late-onset epilepsy. (PDF) [file pone.0336588.s014.pdf]

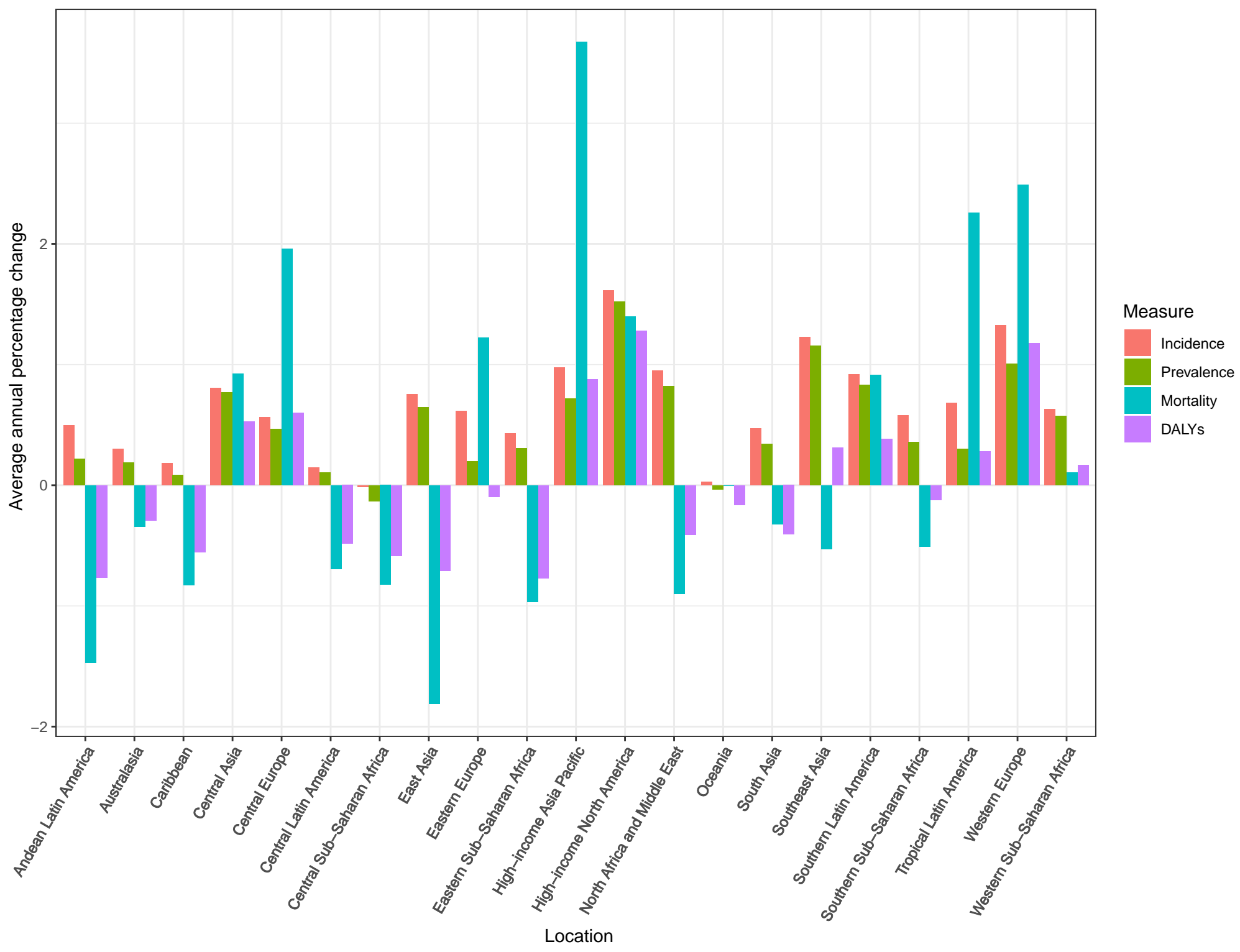

Supplement: S10 Fig — Abbreviations: ASIR, age-standardized incidence rate; ASPR, age-standardized prevalence rate; ASMR, age-standardized mortality rate; DALYs, disability-adjusted life-years; LOE, late-onset epilepsy. (PDF) [file pone.0336588.s015.pdf]

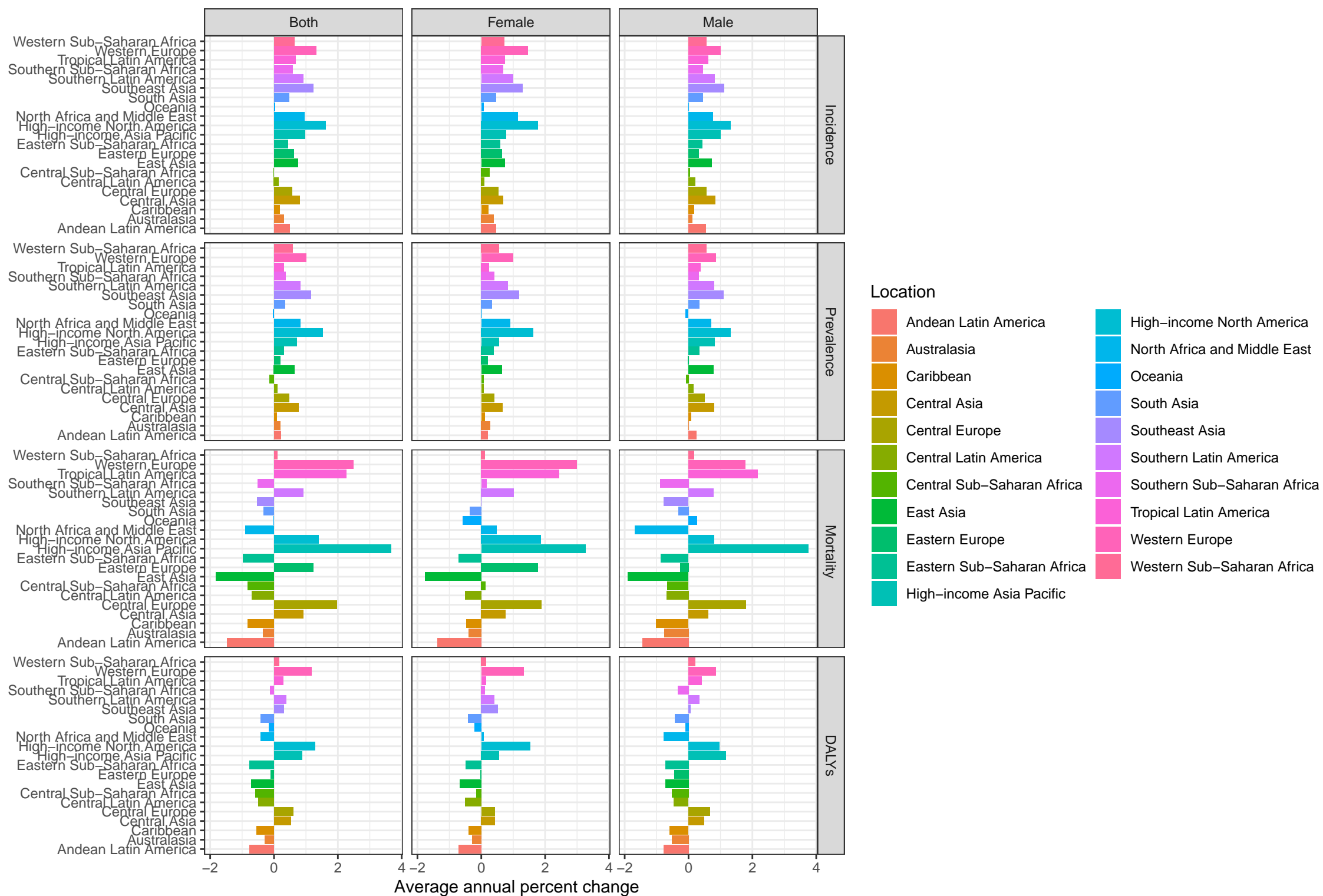

Supplement: S11 Fig — Abbreviations: ASIR, age-standardized incidence rate; ASPR, age-standardized prevalence rate; ASMR, age-standardized mortality rate; DALYs, disability-adjusted life-years; LOE, late-onset epilepsy. (PDF) [file pone.0336588.s016.pdf]

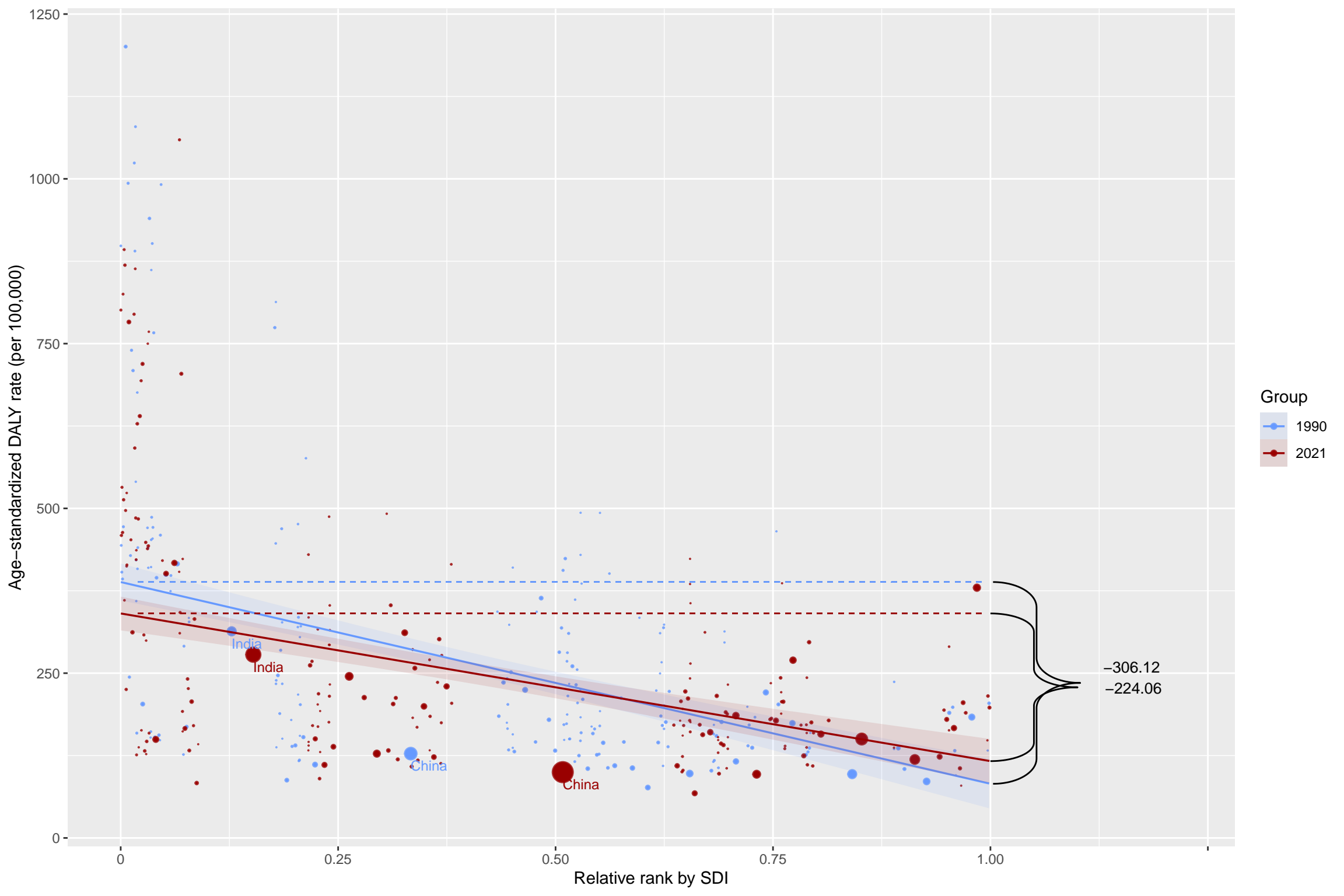

Supplement: S12 Fig — Abbreviations: SDI, sociodemographic index; DALYs, disability-adjusted life-years; LOE, late-onset epilepsy. (PDF) [file pone.0336588.s017.pdf]

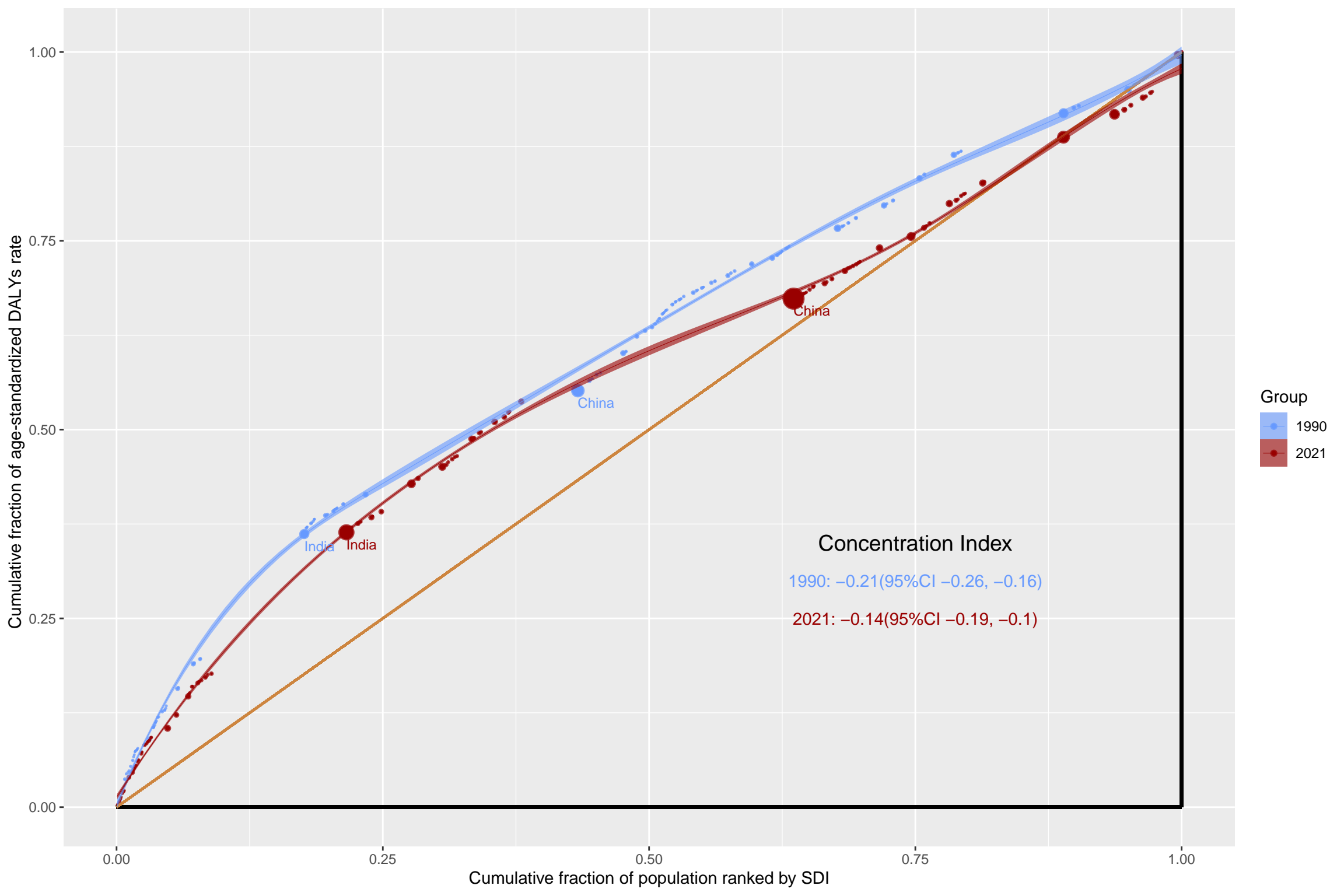

Supplement: S13 Fig — Abbreviations: SDI, sociodemographic index; DALYs, disability-adjusted life-years; LOE, late-onset epilepsy. (PDF) [file pone.0336588.s018.pdf]
